# Unraveling the Morphological and Functional Maturation Mechanisms Underlying Human Neural Development Using iPSCs‐Derived Neuronal Model

**DOI:** 10.1002/advs.202512891

**Published:** 2026-01-11

**Authors:** Yue Tian, Yi‐Chun Ou, Zi‐Xian Zhang, Jie Cai, Si‐Qing Cai, Guo‐Gang Xing

**Affiliations:** ^1^ Department of Neurobiology School of Basic Medical Sciences Peking University Health Science Center and Neuroscience Research Institute Peking University Beijing China; ^2^ Key Laboratory for Neuroscience Ministry of Education of China & National Health Commission of China Beijing China

**Keywords:** human induced pluripotent stem cells (hiPSCs), human neural development, hiPSCs‐derived neuron, voltage‐gated calcium channels, voltage‐gated sodium ion channels, endothelin converting enzyme‐like 1 (ECEL1), calmodulin 3 (CALM3)

## Abstract

Emerging human induced pluripotent stem cells (hiPSCs)‐based neuronal models are useful for studying human neural development. However, existing protocols for differentiating neurons from hiPSCs generally require extended timeframes, making it difficult to capture the rapid, early stages of neuronal morphogenesis and functional maturation. This study presents an in vitro human neuronal model derived from hiPSCs with rapid morphological and functional maturity, by using the combined small molecules and proteins (SMP) protocol. This SMP‐induced, hiPSC‐derived neuronal model recapitulates core aspects of human neuronal development, providing a temporally compressed system for studying early neuronal development. On the basis of this model, this study demonstrates that both Cav1.2 and Cav1.3, the two subtypes of L‐type voltage‐gated calcium channels that mediate calcium ion influx, are essential for early morphogenesis of human neuronal development. Moreover, *ECEL1* (endothelin converting enzyme‐like 1) is identified as a key regulator of human neuronal functional developmental maturation in the early stage of SMP‐induced hiPSCs differentiation. ECEL1 acts through calmodulin 3 (CALM3) to regulate functional assembly and expression of multiple ion channels (e.g., voltage‐gated sodium ion channels) in neuronal functional development and maturation. These findings illuminate novel mechanisms underlying the morphogenesis and functional maturation of human neurons that are involved in human brain development.

## Graphical abstract

Using human induced pluripotent stem cells (hiPSCs)‐derived neuronal model, Tian and colleagues reveal that voltage‐gated calcium channels Cav1.2 and Cav1.3, and their mediated calcium ion influx, are essential for early morphogenesis of human neuronal development, while ECEL1 underlies human neuronal functional developmental maturation through CALM3‐mediated ion channels assembly in neuronal functional development.

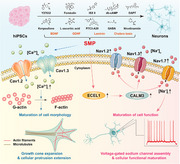

## 1 Introduction

The pace of human brain development is significantly different from that of most other species [1, 2, 3]. Understanding the mechanisms underlying the human‐specific features of neural development is crucial for uncovering the basis of human intellectual abilities and the etiology of neurological and psychiatric disorders [2, 4, 5, 6, 7]. Due to the lack of human neural tissue samples and ethical issues, various animal models, including C. elegans [8], drosophila [9], zebrafish [10], and rodents [11, 12], have been used for neural development research. However, basic differences between humans and these models raise questions about the usefulness of information obtained mainly from animal models, especially the commonly used rodents [12]. Actually, there are many features unique to human brain development that cannot be recapitulated in rodent systems [5, 13].

Primates, especially non‐human primates like macaques and chimpanzees, share a high degree of genetic similarity with humans [14, 15]. This makes them the closest models for studying human brain development. However, using primates increases the cost and complexity of research compared to smaller model organisms like rodents. Moreover, studies have shown that the brain development mechanisms of primates are not entirely similar to those of humans, making it difficult to fully model human neural development [16, 17]. In addition, the failure of clinical trials that have been based on animal studies further highlights the limitations of animal‐model‐focused human disease research [18]. Therefore, to study human brain development, alternative experimental systems that closely mimic the human brain and are both accessible and ethically acceptable should be used.

The emergence of human‐induced pluripotent stem cells (iPSCs) has provided an invaluable tool for creating human‐based culture systems, enabling the modeling and analysis of human neural development in vitro [19, 20]. Human iPSCs (hiPSCs) can be generated from somatic cells by the introduction of reprogramming factors [21, 22]. This technique revolutionizes the direct reprogramming of mature somatic cells into pluripotent stem cells in vitro, eliminating the requirement for embryos [23]. These hiPSC‐based differentiation cultures often generate multiple targeted cell types, making the system more suitable for studying brain development mechanisms, disease modeling, and large‐scale compound screening [24, 25, 26, 27, 28, 29, 30]. Furthermore, hiPSC‐derived neurons can model the morphological changes, electrophysiological functions, gene expression profiles of neurons, and the structural changes of neural networks during human brain development [31, 32, 33, 34]. However, existing differentiation protocols for generating neurons from hiPSCs, such as dual small mothers against decapentaplegic (SMAD) inhibition (SMADi) [35, 36] or forced expression of neurogenic transcription factors [37, 38, 39], usually require a prolonged period, where the cell typically undergo commitment to neural progenitor cells (NPCs) over 2–3 weeks, followed by differentiation into neurons over 4–12 weeks, thereby failing to capture the rapid, early events of human neuronal morphogenesis and functional maturation [17, 40, 41]. These limitations highlight the necessary to develop a new, extended differentiation protocol that can drive hiPSCs toward a functional neuronal fate rapidly and synchronously, particularly for modeling early neural developmental processes.

Moreover, the mechanisms underlying the early morphological and functional development of human neurons constitute a long‐standing topic of interest in neuroscience. Using neuronal models from other animals, some researchers have discovered that key signaling pathways, such as ROCK and calcium signaling, may be involved in early neuronal morphological development and promote neurite outgrowth [42, 43, 44, 45]. Additionally, the outgrowth of neuronal protrusion involves the complex regulation of the polymerization and assembly of various cytoskeletal proteins, including actin, tubulin, and neurofilaments [46, 47, 48], which have not yet been fully explored. With respect to the functional development of human neurons, there have been no effective tools to explore the functional changes and regulatory mechanisms during neuronal development. In recent years, researchers have increasingly utilized neurons differentiated from human iPSCs, combined with techniques such as gene editing, electrophysiology, and single‐cell sequencing, to elucidate key regulatory mechanisms underlying the functional development of human neurons [32, 41, 49, 50]. However, detailed investigations into how key genes, such as ion channel genes, are regulated, expressed, and assembled during the functional maturation of human neurons are still lacking.

In the present study, we aimed to develop an in vitro human neuronal model derived from hiPSCs with relatively mature morphology and functionality, by using a new, extended differentiation protocol, a small molecules and proteins (SMP) protocol, to investigate human early neurodevelopmental mechanisms. This model provides a unique platform to capture the dynamic processes and underlying mechanisms governing morphological and functional development during the early stages of human neuronal maturation. We revealed that voltage‐gated calcium channels Cav1.2 and Cav1.3, and their mediated calcium ion influx, are essential for early morphogenesis of human neuronal development. Moreover, we identified a novel gene, *ECEL1* (endothelin converting enzyme‐like 1), which regulates human neuronal functional developmental maturation, through calmodulin 3 (CALM3)‐mediated multiple ion channels assembly (e.g., voltage‐gated sodium ion channels) in neuronal functional development maturation. Our findings illuminate novel mechanisms underlying the morphological and functional maturation of human neurons that are involved in human brain development.

## 2 Results

### 2.1 Development of An In Vitro Human Neuronal Model Derived from Human Induced Pluripotent Stem Cells (hiPSCs) Using a Combined Small Molecules and Proteins (SMP) Protocol

The study of neurons is fundamental to unraveling the complexities of the nervous system in particular the neural development. Primary neuronal cultures from rodents have long been a cornerstone of experimental studies, yet limitations related to their non‐human nature and ethical concerns have prompted the development of alternatives [51, 52]. In recent years, the derivation of neurons from human induced pluripotent stem cells (hiPSCs) has emerged as a powerful option, offering a scalable source of cells for diverse applications [22, 23, 53, 54]. Human neural cell models derived from hiPSCs have been widely accepted to model various neurodevelopmental and neurodegenerative diseases in vitro [29, 55, 56, 57]. To develop an in vitro human neuronal model derived from hiPSCs for human neural development studies, we first generated urine‐derived hiPSCs by reprogramming epithelial cells collected from the fresh urine of healthy donors [58, 59]. Urine‐derived epithelial cells were isolated from urine samples and cultured according to the procedure described by Zhou et al. [59], resulting in the acquisition of more than 1.0 × 10^7^ cells (Figure S1A,B). The renal epithelial origin of the isolated and expanded cells at passage 2 or 3 was validated using immunofluorescent staining. The results showed that approximately 94.0 ± 1.3% and 91.9 ± 2.4% of the cells were positively expressed renal tubular epithelial cells markers, CD13 and mineralocorticoid receptor (MCR), respectively, while almost 100% of the cells were expressed the tight junction marker zonula occludes‐protein 1 (ZO‐1) (Figure S1C,D), indicating a high isolation and proliferation rate of urine‐derived epithelial cells in vitro. Human iPSCs were generated from 2–4 passages urine‐derived epithelial cells using electroporation of episomal plasmids (pCXLE‐hOCT3/4‐shp53‐F, pCXLE‐hSK, pCXLE‐hUL, and pCXWB‐EBNA1) expressing four reprogramming factors, including octamer‐binding transcription factor 4 (OCT4) gene, sex determining region Y (SRY)‐box 2 (SOX2) gene, Kruppel‐like factor 4 (KLF4) gene, and Myc family genes member L‐Myc [60, 61, 62]. The transfected cells were cultured on Matrigel‐coated and serum‐free reprogramming medium for in vitro cellular reprogramming (Figure S1E). The hiPSCs colonies displayed a typical human pluripotent stem cell morphology (e.g., compact colonies, high nucleus‐to‐cytoplasm ratios, and prominent nucleoli) [22, 63] at approximately 20 days after electroporation (Figure S1F), and expressed alkaline phosphatase (ALP), a phenotypic marker of undifferentiated human pluripotent stem cells (hPSCs) (Figure S1G). Reverse transcription polymerase chain reaction (RT‐PCR) analysis showed that the hiPSCs expressed many undifferentiated embryonic stem (ES) cell‐marker genes, such as *OCT3/4*, *SOX2*, Nanog homeobox (*NANOG*), reduced expression 1 (*REX1*), developmental pluripotency‐associated 5 (*DPPA5*), and DNA methyltransferase 3 beta (*DNMT3B*), at levels equivalent to those in the human ES cell (hESC) lines H1 and H9 (Figure S1H). The results from immunofluorescence staining revealed that most hiPSCs expressed specific markers of human pluripotent stem cells (PSCs), including the proteins of OCT3/4, NANOG, SOX2, as well as the cell surface markers such as stage‐specific embryonic antigen (SSEA)‐4, tumor‐related antigens (TRA)‐1‐60, and TRA‐1‐81 (Figure S1I). Bisulfite genomic sequencing analysis was performed to assess the methylation statuses of cytosine‐guanine dinucleotides (CpG) in the promoter regions of pluripotent‐associated genes, such as *OCT3/4* and *NANOG*. The results revealed that these genes promotor was highly unmethylated in urine‐derived hiPSCs, whereas the CpG dinucleotides of the regions were highly methylated in parental urine‐derived epithelial cells (U‐Cells) (Figure S1J). These data indicate that these pluripotent‐associated genes promoter are active in hiPSCs. In addition, results from chromosomal G‐band analysis of the hiPSCs (>15 passages) demonstrated that these urine‐derived hiPSCs also exhibited normal diploid 46, XY karyotype (Figure S1K).

Then, we examined the differentiation ability of the urine‐derived hiPSCs by using a floating culture method to form embryoid bodies (EBs) in vitro [21]. After 8 days in suspension culture, the hiPSCs began to form ball‐shaped structures, and then they were transferred to gelatin‐coated plates for another 8‐day adherent culture (Figure S1L). The results from RT‐PCR analysis revealed that these differentiated cells expressed the ectoderm markers, including microtubule‐associated protein 2 (*MAP2*), neuronal differentiation 1 (*NEUROD1*), and glial fibrillary acidic protein (*GFAP*), as well as the mesoderm markers, such as Msh homeobox 1 (*MSX1*), T‐Box transcription factor T (*BRACHYURY*), and the endoderm markers, eg., cytokeratin 8 (*CK8*), SRY‐box containing gene 17 (*SOX17*), and alpha fetoprotein (*AFP*). In contrast, the expression of multiple pluripotency markers, such as *OCT3/4*, *SOX2*, and *NANOG* was markedly decreased (Figure S1M). Also, the results from immunofluorescence staining verified that these differentiated cells were positive for expressing the ectoderm markers, including NESTIN, GFAP, and tubulin beta III (TUJ1), as well as the mesoderm marker CD31 and the endoderm marker SOX17 (Figure S1N). Similar to human ES cells, when injected into immunocompromised mice, the urine‐derived hiPSCs could form teratomas that consists of differentiated derivatives of all three primary germ layers in vivo, such as neural tissue (ectoderm), muscle and adipose tissues (mesoderm), and epithelium tissue (endoderm) (Figure S1O). These results demonstrate that the urine‐derived hiPSCs could differentiate into three germ layers both in vitro and in vivo. Taken together, these findings suggest that the iPSCs generated from urinary‐derived epithelial cells exhibit the fundamental characteristics of human pluripotent stem cells, thereby generating a noninvasive, virus‐free, urine‐derived hiPSCs from human urine‐derived epithelial cells under feeder‐free conditions.

**FIGURE 1:**
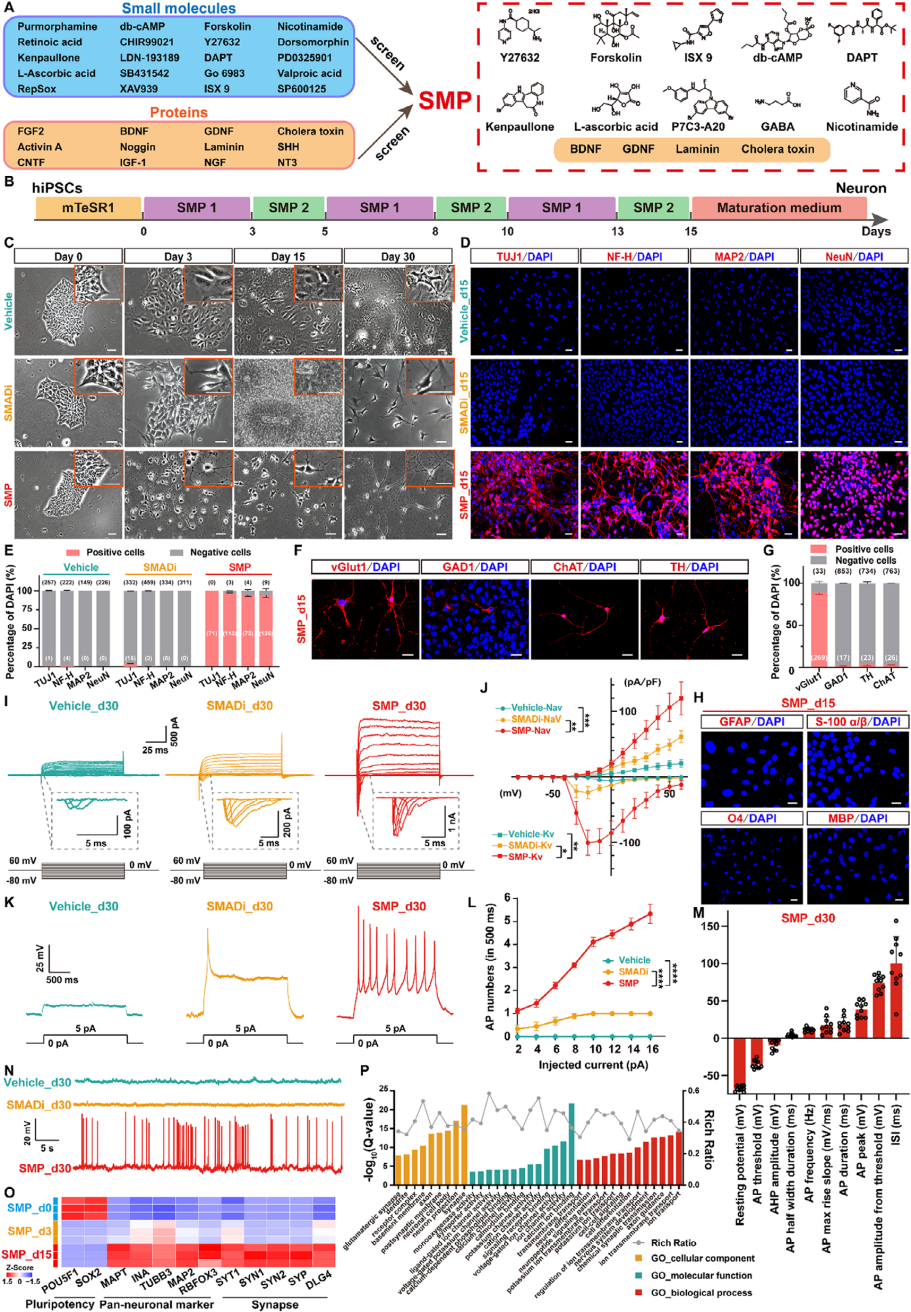
Development of an in vitro human neuronal model derived from human iPSCs (hiPSCs) using a combined small molecules and proteins (SMP) protocol. (A) Diagram illustrating the experimental procedure for screening small molecule compounds and proteins in the SMP combination. Left: candidate small molecule compounds (upper) and recombinant proteins (bottom) are listed. Right: 10 small molecule compounds and 4 recombinant proteins that composed the SMP combination are shown. (B) Schematic diagram depicting the time schedule for differentiating iPSCs into neurons using the SMP protocol. (C) Representative phase‐contrast images showing morphological changes of hiPSCs between the SMP‐, SMADi (dual SMAD inhibition)‐, and vehicle (neurobasal/B‐27 media containing 0.1% DMSO)‐treated groups (n = 3 biological replicates per group). Scale bar = 50 µm. Insets in the upper right corner showing higher magnification images, scale bar = 50 µm. (D) Representative images showing the immunofluorescence staining with neural lineage‐specific markers (TUJ1, MAP2, NF‐H, and NeuN) in hiPSCs‐differentiated cells at 15 days in vitro (DIV) culture in the SMP‐, SMADi‐, or vehicle‐treated groups. TUJ1, MAP2 and NF‐H are markers of the neuronal cytoskeleton. NeuN is a neuron‐specific nuclear protein. Cell nuclei were counterstained with DAPI (blue). Scale bar = 20 µm. (E) Bar graph showing the percentage of neuronal marker^+^ cells in DAPI^+^ cells (n = 3 biological replicates per group). (F) Representative images showing the immunofluorescence staining with neuronal subtype markers (vGlut1, GAD1, ChAT, and TH) in hiPSCs‐differentiated cells at 15 DIV culture using the SMP protocol. vGlut1 is a specific marker for glutamatergic neurons. GAD1 is a specific marker for GABAergic neurons. ChAT is a specific marker for cholinergic neurons. Tyrosine hydroxylase (TH) is a specific marker for dopaminergic neurons. Cell nuclei were counterstained with DAPI (blue). Scale bar = 20 µm. (G) Bar graph showing the percentage of neuronal subtype marker^+^ cells in DAPI^+^ cells (n = 3 biological replicates per group). (H) Representative images showing the immunofluorescence staining with glia‐specific markers (GFAP, S100α/β, O4, and MBP) in hiPSCs‐differentiated cells at 30 DIV culture using the SMP protocol (n = 3 biological replicates per group). GFAP and S100α/β are specific markers for astrocytes. O4 and MBP are specific markers for oligodendrocytes. Cell nuclei were counterstained with DAPI (blue). Scale bar = 20 µm. (I) Representative traces showing the voltage‐gated sodium currents and potassium currents recorded from hiPSCs‐differentiated cells at 30 DIV culture between the SMP‐, SMADi‐, and vehicle‐treated groups. (J) Current‐voltage (I‐V) relationships of voltage‐gated sodium currents (inward currents, circles) and potassium currents (outward currents, squares) recorded from hiPSCs‐differentiated cells at 30 DIV culture in the SMP‐ (red traces), SMADi‐ (yellow traces), and vehicle (cyan traces)‐treated groups. Currents were evoked by 100 ms depolarizing steps from a holding potential of ‐80 mV to test potentials ranging from ‐80 to +60 mV (n = 8 cells per group). (K) Representative traces showing the evoked action potentials with the injection of a 5‐pA depolarized current pulse in current‐clamp mode, recorded from a hiPSCs‐differentiated cells at 30 DIV culture in the SMP‐ (red traces), SMADi‐ (yellow traces), and vehicle (cyan traces)‐treated groups. Scale bar = 50 mV, 500 ms. (L) Input‐output relationship of the hiPSCs‐differentiated cells in response to a series of depolarizing current pulses from 2 pA to 16 pA in 2 pA increments at 30 DIV culture in the SMP‐ (red traces), SMADi‐ (yellow traces), and vehicle (cyan traces)‐treated groups (n = 9 cells per group). (M) Bar graph showing the intrinsic electrogenic properties of the SMP‐induced, hiPSCs‐differentiated neurons (n = 8 cells), inferred by resting membrane potential (RMP), action potential (AP) threshold, after‐hyperpolarization (AHP) amplitude, AP half‐width duration, AP frequency, AP duration, AP maximum rise slope, AP peak, AP amplitude from threshold, and AP inter‐spike interval (ISI). (N) Representative traces showing the spontaneous action potentials recorded from a hiPSCs‐differentiated neuron at 30 DIV culture in the SMP‐ (red traces), SMADi‐ (yellow traces), and vehicle (cyan traces)‐treated groups. Scale bar = 20 mV, 5 s. (O) Heatmap showing the normalized differentially expressed genes (DEGs) levels of pluripotency and neural‐lineage genes across the SMP_d0, SMP_d3, and SMP_d15 groups by RNA‐seq analysis (n = 3 biological replicates per group). Z‐score normalization was performed along the columns. (P) Histogram showing the Gene Ontology (GO) enrichment derived from the upregulated DEGs between the SMP_d15 group and the SMP_d0 group by RNA‐seq analysis (n = 3 biological replicates per group). The horizontal axis shows the GO annotation classification, while the left vertical axis displays the ‐log_10_(Q‐value), a measure of statistical significance, and the right vertical axis shows the rich ratio, which indicates the proportion of genes in the sample that are annotated with a specific GO term. Data are presented as mean ± SEM. ^*^ *p*<0.05; ^**^ *p*<0.01; ^***^ *p*< 0.001; ^****^ *p*< 0.0001. Repeated‐measures two‐way ANOVA with Sidak's *post‐hoc* test for (J) and (L). See also Figures S1–S3.

To overcome the extended timeline of conventional approaches and better access the initial phases of neuronal development [38, 41, 64], we developed a rapid induction strategy by using a new, extended differentiation protocol, a small molecules and proteins (SMP) protocol, to differentiate hiPSCs into neurons in vitro [65]. First, we designed a compound library consisting of multiple candidate small molecules from relevant literature, which drive neuronal differentiation and maturation [66, 67, 68, 69, 70, 71, 72, 73, 74, 75]. These small molecules included agonists and inhibitors of several signaling pathways associated with neuronal development and maturation [76, 77, 78, 79]. In addition, soluble proteins play a crucial role in neuronal differentiation by interacting with cell receptors, interfering with signaling pathways, influencing cell metabolism, promoting the differentiation of pluripotent stem cells into neural lineages, and nourishing neurons [80, 81, 82, 83, 84, 85, 86]. Consequently, several recombinant proteins were included in the protein screening library. Starting from the aforementioned candidate library of small molecules and recombinant proteins with reported roles in neuronal development (Figure 1A), different combination strategies were respectively tested in hiPSCs cultures. The primary screening criterion was the ability to induce neuronal differentiation within a short 3‐day timeframe. Each condition was evaluated based on morphological progression (formation of neurites and interconnected neural networks) and electrophysiological competence (ability to generate action potentials upon depolarization pulse) observed at this early time point. Combinations that consistently produced robust neurite outgrowth and generated cells capable of firing action potentials within a 3‐day period were prioritized, whereas formulations leading to poor morphology or failure to elicit action potentials were excluded. Following over 50 rounds of screening, a small‐molecule cocktail containing Y27632 [a selective inhibitor of p160‐Rho‐associated coiled‐coil kinase (ROCK)], forskolin (an adenylate cyclase activator), isoxazole‐9 (ISX‐9, an isoxazole small molecule possessing neuronal‐differentiating properties), db‐cAMP [dibutyryl cyclic adenosine monophosphate (cAMP), a membrane‐permeable protein kinase A (PKA) activator that acts by mimicking endogenous cAMP], *N*‐[*N*‐(3,5‐difluorophenacetyl)‐L‐alanyl]‐*S*‐phenylglycine t‐butyl ester (DAPT, a γ‐secretase inhibitor, forces cell cycle exit and promotes functional maturation of iPSC‐derived neurons), kenpaullone (an inhibitor of cyclin‐dependent kinases 1 and glycogen synthase kinase 3β), L‐ascorbic acid (vitamin C, an antioxidant possessing neuronal‐differentiating properties), P7C3‐A20 (a neuroprotective aminopropyl carbazole compound), γ‐aminobutyric acid (GABA, a neurotransmitter that promotes neuronal maturation), and nicotinamide (vitamin B_3_, a morphogen in the differentiation of stem cells to mature neuron), together with 4 proteins, including brain‐derived neurotrophic factor (BDNF), glial cell line‐derived neurotrophic factor (GDNF), laminin, and cholera toxin (a cAMP stimulator), which were named as the *Small Molecule Compounds* and *Protein Combinations* (SMP) (Figure 1A), were identified capable of promoting the rapid differentiation of hiPSCs into neurons with high efficiency.

Although the SMP treatment resulted in a high differentiation efficiency of hiPSCs into neurons, severe cell death was observed with continuous exposure of long‐term culture for cells as reported previously [87]. After several rounds of trials, we modified the treatment strategy by splitting the SMP protocol into two distinct sets as follows: a SMP1 set, which contained all components of the aforementioned SMP formulation, and a SMP2 set, which comprised a part of the SMP formulation including db‐cAMP, DAPT, L‐ascorbic acid, GABA, BDNF, GDNF, and laminin. We found that using the modified protocol by alternately adding the SMP1 medium for 3 days culture and then adding the SMP2 medium for another 2 days culture, repeated 3 cycles from 1 day in vitro (DIV) to 15 DIV, the differentiated cells exhibited both high conversion efficiency and improved survival rate (Figure 1B,C). After 15 days culture in vitro, the induction medium was switched to a maturation medium that lacking small molecules, for promoting the neuronal survival and maturation (Figure 1B). During dynamic monitoring of the whole conversion process, significant neuron‐like morphological changes were observed at 3 DIV, compared to the vehicle (neurobasal/B‐27 media containing 0.1% DMSO) group and the SMADi‐treated group (using the widely‐used dual SMAD inhibition protocol for neuronal differentiation [35, 36]) (Figure 1C). These cells interconnected throughout the conversion processes, forming a neural network‐like structure. With the extension of culture time from 3 DIV to 30 DIV, the conversion processes continued and the differentiated cells began to extend, branch, and thicken, resulting in a typical neuronal morphology at 30 DIV (Figure 1C). In addition, results from immunostaining with a series of identified neuronal markers, including TUJ1, neurofilament heavy chain (NF‐H), MAP2, and neuronal nuclei antigen (NeuN), revealed that, all of these neuronal markers were highly expressed in the SMP‐induced hiPSCs‐differentiated cells at 15 DIV, compared to the vehicle group and the SMADi‐treated group, with positive percentage of 100%, 97.4 ± 0.18%, 94.6 ±1.7%, and 94.2% ± 2.9%, respectively (Figure 1D,E), thereby validating the neuronal properties of the differentiated cells by morphological characteristics. Notably, the SMP‐induced, hiPSCs‐differentiated cells achieved a significantly higher level of neuronal differentiation as early as 15 DIV, with extensive neurite networks formed. At the same time point, however, the SMADi‐treated group still exhibited neural rosette‐like structures, and displayed minimal neuronal marker expression (TUJ1: 4.3 ± 0.06%; with more lower levels for NF‐H, MAP2, and NeuN) (Figure 1C‐E). Moreover, immunostaining with glutamatergic neuronal marker vesicular glutamate transporter 1 (vGlut1), GABAergic neuronal marker glutamate decarboxylase 1 (GAD 1), cholinergic neuronal marker choline acetyltransferase (ChAT), dopaminergic neuronal marker tyrosine hydroxylase (TH), as well as astrocyte markers, GFAP and S100 α/β, and oligodendrocyte markers, O4 and myelin basic protein (MBP), revealed that, approximately 88.7 ± 2.0% of the differentiated cells were positively expressed vGlut1, whereas the positive expression of GAD1, ChAT, and TH was 2.0 ± 0.3%, 3.3 ± 0.4%, and 3.7 ± 1.4%, respectively (Figure 1F,G). With respect to the expression of astrocyte markers (GFAP and S100 α/β) and oligodendrocyte markers (O4 and MBP), almost no positive immunostaining signals were detected in the differentiated cells (Figure 1H). These data suggest that the majority of the differentiated cells were glutamatergic neurons, therefore, this SMP‐induced differentiation strategy mainly induces the conversion of hiPSCs toward an excitatory neuronal fate.

To determine whether the hiPSCs differentiated‐neuron‐like cells are electrophysiological mature in addition to the possession of morphological and immunocytochemical neuronal characteristics, we examined the electrophysiological properties of the differentiated cells, by using whole‐cell patch clamp recording of the neuron‐like cells at 30 DIV as described previously [87, 88, 89]. The results showed that, both rapidly inactivating inward voltage‐gated sodium (Na^+^) currents and persistent outward voltage‐gated potassium (K^+^) currents, were recorded in the SMP‐induced, hiPSCs‐differentiated neuron‐like cells in response to 100 ms‐depolarizing voltage‐clamp steps (Figure 1I), and were significantly greater than those in both vehicle‐treated cells (SMP‐Nav vs. vehicle‐Nav: *p* = 0.0006; SMP‐Kv vs. vehicle‐Kv: *p* = 0.0016, Figure 1I,J) and SMADi‐treated cells (SMP‐Nav vs. SMADi‐Nav: *p* = 0.0025; SMP‐Kv vs. SMADi‐Kv: *p* = 0.0384; Figure 1I,J). The current‐voltage (I‐V) relationship revealed that, the voltage‐gated sodium channels in SMP‐induced neuron‐like cells began to activate at a holding voltage of ‐40 mV, with the average channel current peaking (‐100.4±19.3 pA/pF) at ‐20 mV before gradually declining (Figure 1J). In parallel, the current of the voltage‐gated potassium channels increased steadily with the holding voltage depolarized from ‐20 mV to +60 mV, reaching an average current peaking of 119.8±25.1 pA/pF at the holding voltage of +60 mV (Figure 1J). These results indicate that the SMP‐induced neuron‐like cells expressed both voltage‐gated sodium channels and potassium channels, which are essential for generating action potentials (APs).

In addition, results from whole‐cell current‐clamp recording showed that, the SMP‐induced neuron‐like cells were able to fire repetitive action potentials with prominent spike adaptation upon depolarization, while the vehicle‐treated cells failed to generate action potentials and the SMADi‐treated cells exhibited only a single action potential upon depolarization (Figure 1K). The SMP‐induced neuron‐like cells also exhibited normal input‐output relationship in response to 2‐pA depolarizing current‐clamp steps, as compared with both vehicle group and SMADi‐treated group, reflecting the functional matured passive membrane properties (SMP vs. vehicle: *p*<0.0001; SMP vs. SMADi: *p*<0.0001, Figure 1L). Moreover, analyses of other intrinsic electrogenic properties of the differentiated cells showed that, the SMP‐induced neuron‐like cells exhibited intrinsic electrogenic properties similar to those of mature neurons [32, 89], including RMP (‐67.8±1.1 mV), action potential (AP) threshold (‐35.4±1.7 mV), after‐hyperpolarization (AHP) amplitude (‐9.4±2.2 mV), AP half‐width duration (3.9±0.9 ms), AP frequency (11.3±0.9 Hz), AP duration (18.2±3.1 ms), AP maximum rise slope (17.1±3.6 mV/ms), AP peak (38.5±3.2 mV), AP amplitude from threshold (73.9±3.4 mV), and AP inter‐spike interval (ISI) (100.1±11.4 ms) (Figure 1M). Notably, ∼10.4% (5/48, n = 48) of the SMP‐induced neurons exhibited more mature electrophysiological properties at 30 DIV, characterized by the spontaneous firing of trains of action potentials without current injection, whereas the vehicle‐ and SMADi‐treated cells at 30 DIV did not exhibit spontaneous action potentials (Figure 1N). Collectively, these results demonstrate that the SMP‐induced, hiPSCs‐differentiated neurons exhibit electrophysiological properties characteristic of mature neurons.

Furthermore, we examined transcriptome profiles of the SMP‐induced neuron‐like cells derived from hiPSCs by using RNA sequencing (RNA‐seq) technique, performed for hiPSCs (SMP_d0 group), and differentiated cells at 3 DIV (SMP_d3 group) and 15 DIV (SMP_d15 group), respectively (Figure S2A). Clustering heatmap of Pearson's correlation coefficient revealed that, the transcriptional profiles of differentiated cells presented high similarity within each group, but exhibited significant differences across the different groups (Figure S2B). Principal component analysis (PCA) showed clear transcriptomic differences among the three groups, illustrating a pathway of hiPSCs differentiation from the SMP_d0 group, pass through the SMP_d3 group, to the SMP_d15 group (Figure S2C). Differentially expressed genes (DEGs) analysis (|log_2_(fold change)| ≥ 2) showed that 1854 DEGs (SMP_d3 vs. SMP_d0), 3742 DEGs (SMP_d15 vs. SMP_d0), and 1157 DEGs (SMP_d15 vs. SMP_d3) were respectively identified across three groups (Figure S2D). Additionally, Heatmap analysis of the DEGs revealed that, in the process of hiPSCs differentiation from SMP_d0 to SMP_d3, and then to SMP_d15, a cluster of genes enriched in hiPSCs (cluster I, supposed pluripotent‐associated genes) were significantly downregulated, whereas another cluster of neuronal differentiation‐related genes (cluster II) were prominently upregulated; among those, most of the upregulated genes were further increased in the SMP_d15 group compared to the SMP_d3 group (Figure S2E). Moreover, Venn diagram analysis revealed that, a significant overlap was found in the DEGs among pairwise comparisons of the different groups, including SMP_d3 vs. SMP_d0, SMP_d15 vs. SMP_d0, and SMP_d15 vs. SMP_d3, with 351 DEGs were identified across all of the comparisons between the different groups (Figure S2F), indicating that these DEGs probably play essential roles in the conversion of hiPSCs into neurons throughout the process of the SMP‐induced differentiation. The volcano plots clearly showed the upregulated genes and the downregulated genes in the process of hiPSCs differentiation, as compared between SMP_d3 vs. SMP_d0, SMP_d15 vs. SMP_d0, and SMP_d15 vs. SMP_d3, respectively (Figure S2G‐I), suggesting that in the process of iPSCs differentiation, multiple genes are involved in different stages of iPSCs differentiation, in which different genes may play their respective roles in the conversion of iPSCs into neurons.

With respect to neuronal differentiation‐associated genes, Heatmap for the normalized gene expression levels of pluripotency and neural lineage genes showed that, in the process of SMP‐induced hiPSCs differentiation (from SMP_d0, pass through SMP_d3, to SMP_d15), the pluripotency‐associated genes were prominently downregulated, whereas the neural differentiation‐associated genes, in particular those genes related to neuronal differentiation and functional activity, such as receptor assembly, synapse formation and synaptic transmission, were significantly upregulated (Figure S3A, and Figure 1O). Moreover, in the process of SMP‐induced hiPSCs differentiation (from SMP_d0, pass through SMP_d3, to SMP_d15), multiple genes related to cellular cytoskeleton, cytoskeleton‐associated proteins, and ion channels assembly also were also upregulated dramatically (Figure S3B,C). Besides, for the upregulated DEGs between the SMP_d15 group and the SMP_d0 group, gene ontology (GO) enrichment analysis revealed that, most upregulated genes are related to the GO annotations about nervous system development, including both morphological aspects (e.g., neuronal cytoskeleton formation) and functional aspects (e.g., ion channel assembly) (Figure 1P). Taken together, these data suggest that multiple genes related to neuronal differentiation, such as neuronal cytoskeleton formation and ion channel assembly, are involved in the process of SMP‐induced, hiPSCs differentiation into neurons. Therefore, the SMP‐induced, hiPSCs‐differentiated neurons are capable of be used as an in vitro human neuronal model for the study of human early neural development.

### 2.2 Identification of the Morphological and Functional Developmental Maturation of hiPSCs‐Derived Neurons in the Process of SMP‐Induced hiPSCs Differentiation

**FIGURE 2:**
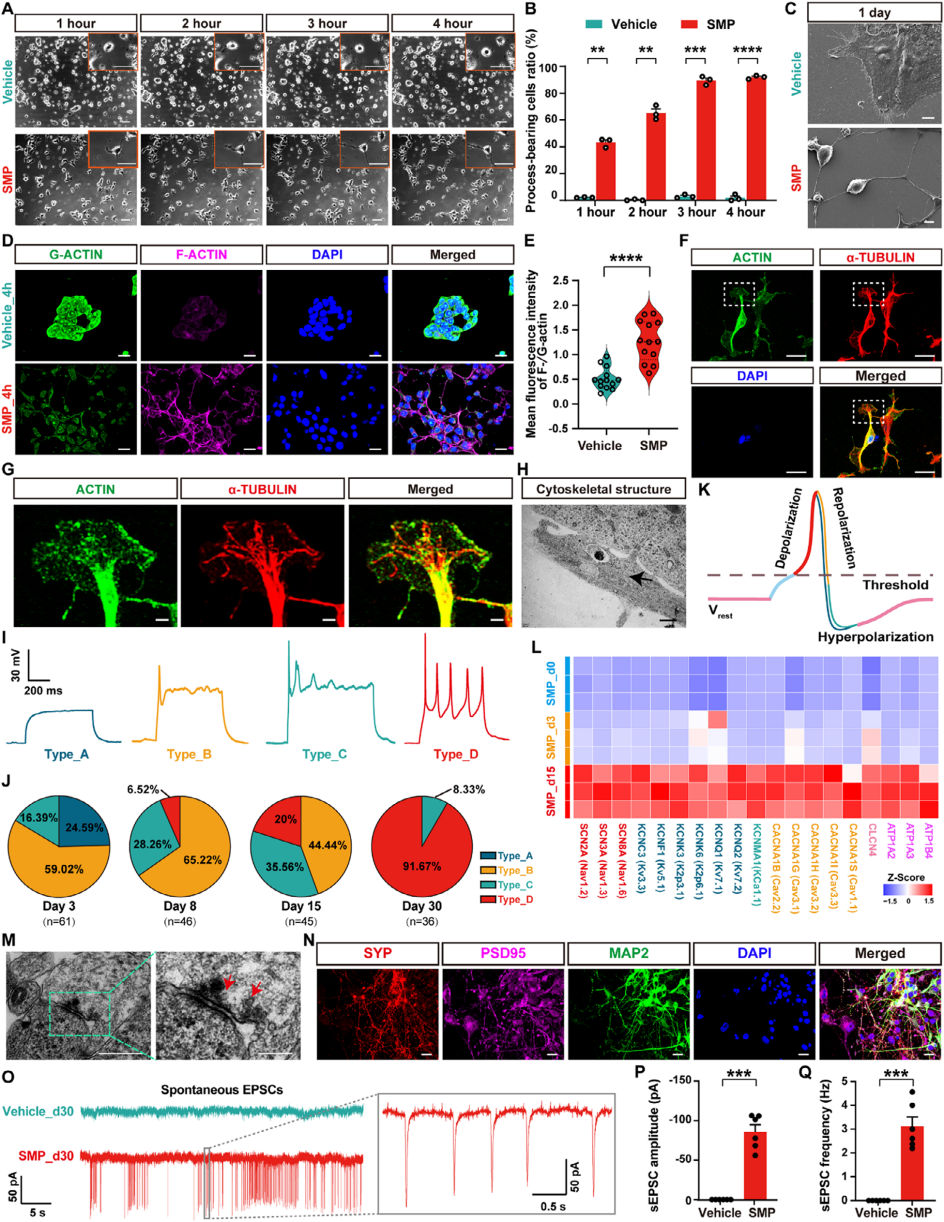
Identification of the morphological and functional developmental maturation of hiPSCs‐derived neurons in the process of SMP‐induced hiPSCs differentiation. (A) Representative phase‐contrast images showing changes of cells in the early stage (e.g., 1, 2, 3 and 4 h) of SMP‐induced hiPSCs differentiation. Control cells were treated with neurobasal/B‐27 media containing 0.1% DMSO (vehicle) for various time points. Scale bar = 50 µm. Insets in the upper right corner showing higher magnification images, scale bar = 50 µm. (B) Bar graph showing the percentage of differentiated process‐bearing cells from hiPSCs at the time points of 1, 2, 3 and 4 h after the SMP induction compared to vehicle (n = 3 biological replicates per group). (C) Representative scanning electron micrograph (SEM) showing the morphological structure of hiPSCs‐differentiated cells cultured with the SMP or vehicle at 1 day in vitro (DIV). Scale bar = 5 µm. (D) Representative images showing the immunofluorescence staining with G‐actin (green) and F‐actin (purple), respectively, in hiPSCs‐differentiated cells cultured with the SMP or vehicle for 4 h. Cell nuclei were counterstained with DAPI (blue). Scale bar = 20 µm. (E) Violin plots showing the F‐/G‐actin ratio between the SMP_4h and the Vehicle_4h groups (n = 13 cells per group). (F) Representative images showing the immunofluorescence staining with actin (green) and tubulin (red), respectively, in hiPSCs‐differentiated cells cultured with the SMP for 4 h (n = 3 biological replicates per group). Cell nuclei were counterstained with DAPI (blue). The white dashed box in the merged image showing the growth cone structure. Scale bar = 20 µm. (G) The higher magnification of neuronal growth cone structure within the white dashed box in (F) are shown. Scale bar = 2 µm. (H) Representative transmission electron micrograph (TEM) showing the cytoskeletal structure of hiPSCs‐differentiated cells cultured with the SMP for 4 h (n = 3 biological replicates per group). Arrow indicates the microfilament structure, and arrowhead indicates the microtubule structure. Scale bars = 500 nm. (I) Representative traces showing the four distinct action potential (AP) patterns recorded from hiPSCs‐differentiated cells from day 3 to day 30 in the process of SMP‐induced hiPSCs differentiation. The types of cells are categorized by their AP patterns as follows: type_A (no action potential), type_B (a single action potential), type_C (immature multiple action potentials), and type_D (mature multiple action potentials). (J) Pie graphs showing the proportion of cell types recorded from hiPSCs‐differentiated cells from day 3 to day 30 in the process of SMP‐induced hiPSCs differentiation (n = 36‐61 cells per group). (K) Schematic diagram shows the distinct phases of a matured action potential, including resting membrane potential (V_rest_) (pink), depolarization (red), repolarization (light blue/orange), and hyperpolarization (light blue/blue‐green). (L) Heatmap showing the normalized gene expression levels of selected transcripts associated with ion channels related to action potentials across the SMP_d0, SMP_d3, and SMP_d15 groups by RNA‐seq analysis (n = 3 biological replicates per group). Z‐score normalization was performed along the columns. (M) Representative TEM showing the structure of cell–cell junctions of hiPSCs‐differentiated cells cultured with the SMP for 4 h (n = 3 biological replicates per group). Left: image shows a formed cell–cell junction between the neurite terminal of one cell and the cell body of another cell. Scale bars = 500 nm. Right: the higher magnification of image from the cyan dashed box in the left shows a synaptic junction‐like structure between the neurite terminal and the cell body indicated by an arrow. Multiple vesicle‐like structures are also observed. Scale bars = 200 nm. (N) Representative images showing the immunofluorescence staining with SYP (red), PSD95 (purple), and MAP2 (green), respectively, in hiPSCs‐differentiated cells at 30 DIV culture using the SMP protocol (n = 3 biological replicates per group). Cell nuclei were counterstained with DAPI (blue). Scale bars = 20 µm. (O) Representative traces show the spontaneous excitatory postsynaptic currents (sEPSCs) recorded from hiPSCs‐differentiated cells at 30 DIV cultured with the SMP or vehicle (n = 6 cells per group). (P) Bar graph showing the statistical analysis of sEPSCs amplitude recorded from hiPSCs‐differentiated cells at 30 DIV cultured with the SMP or vehicle (n = 6 cells per group). (Q) Bar graph showing the statistical analysis of sEPSCs frequency recorded from hiPSCs‐differentiated cells at 30 DIV cultured with the SMP or vehicle (n = 6 cells per group). Data are presented as mean ± SEM. ^**^ *p*<0.01; ^***^ *p*< 0.001; ^****^ *p*< 0.0001. Repeated‐measures two‐way ANOVA with Sidak's *post‐hoc* test for (B); Two‐tailed unpaired *t* test with Welch's correction for (E), (P) and (Q). See also Figure S4.

To attest whether the SMP‐induced, hiPSCs‐differentiated neurons could be used as an in vitro human neuronal model for the study of human early neural development, we examined the morphological and functional developmental characteristics of hiPSCs‐derived neurons in the process of SMP‐induced hiPSCs differentiation. In view of the neuronal morphological development, neurites are the most important morphological features of neurons, with neurite initiation marking the initial stage of neuronal morphogenesis [46, 90]. Filopodial and lamellipodial structures form nascent growth cones that protrude from the cell body to extend neurites. These neurites differentiate into the axons and dendrites of mature neurons, establishing the intricate circuitry of the entire nervous system [48, 90, 91]. To investigate the morphological development of hiPSCs‐derived neurons in the process of SMP‐induced hiPSCs differentiation, we monitored the differentiated cells dynamically using an automated microscopic imaging system to continuously capture images of these cells after plating under the vehicle (Video S1‐1) or SMP condition (Video S1‐2). Surprisingly, the initial neurite extension from the cell body emerged rapidly, and was observable as early as 30 to 60 min after plating under the SMP medium culture. Within the first 4 h after plating, the proportion of differentiated process‐bearing cells to total hiPSCs increased steadily in the SMP group, as compared with the vehicle group (SMP_1h 43.2±2.0% vs. vehicle_1h 2.1±0.2%, *p* = 0.0083; SMP_2h 65.4±2.9% vs. vehicle_2h 0.3±0.3%, *p* = 0.0079; SMP_3h 89.5±1.8% vs. vehicle_3h 3.0±0.7%, *p* = 0.0003; SMP_4h 91.9±0.8% vs. vehicle_4h 1.8±1.4%, *p*<0.0001; Figure 2A,B). Images of scanning electron microscope (SEM) showed that, the SMP‐induced process‐bearing cells exhibited typical neuronal morphology, including long and branched cellular protrusion that interconnected with other cells, at 1 day in vitro (DIV), while the vehicle‐treated cells maintained a flattened epithelial‐like morphology (Figure 2C).

The growth cone is the starting point for neurite outgrowth, where actin filaments continuously polymerize at the leading edge of the growth cone, transitioning from monomeric G‐actin to filamentous F‐actin, and triggering the extension of filopodia and lamellipodia [46, 92]. At the tips of these filopodia, actin filaments undergo depolymerization and provide a pathway for microtubules to extend and elongate [93, 94], transitioning from a polymerized to a stabilized state, to form the newly generated axonal shaft [94, 95]. To determine the dynamic changes of actin in hiPSCs‐derived neurons in the process of SMP‐induced hiPSCs differentiation, we examined F‐/G‐actin ratio of the differentiated cells, that were cultured in the SMP medium (SMP_4h group) or vehicle (vehicle_4h group) at 4 h after plating. Immunostaining with deoxyribonuclease I (DNase I, for labelling G‐actin) and phalloidin (for labelling F‐actin) [96, 97] revealed that, the F‐/G‐actin ratio was increased significantly, from 0.51±0.06 in the vehicle_4h group to 1.28±0.11 in the SMP_4h group (*p*<0.0001, vs. vehicle_4h group, Figure 2D,E), indicating an elevated transition from G‐actin to F‐actin in the early stage of the SMP‐induced hiPSCs differentiation. Similarly, Western blotting of the differentiated cells also verified the increased F‐/G‐actin ratio in the SMP_4h group (0.22±0.04) in contrast to the vehicle_4h group (0.03±0.01) (*p* = 0.0104, vs. vehicle_4h group, Figure S4A,B). These findings indicate that the monomeric G‐actin begins to polymerize into filamentous F‐actin in the differentiated cells at the early stage of hiPSCs differentiation, which is probably responsible for initiating the early extension of cell neurites in the process of SMP‐induced hiPSCs differentiation.

To further investigate the morphological developmental features of hiPSCs‐derived neurons in the early stage of SMP‐induced hiPSCs differentiation, we examined the growth cone structures and the neurite outgrowth of hiPSCs‐differentiated cells, at 4h SMP‐induction (SMP_4h). Images of high‐magnification confocal microscopy revealed that, the actin was distributed within the cell body and at the tips of cellular protrusion, whereas the α‐tubulin was mainly localized along the main backbone of the cellular protrusion (Figure 2F). Magnified images of growth cone structures showed that, the distal ends of the cellular protrusion enriched actin, while the central structure of the growth cone contained microtubules (Figure 2G). In fact, images of transmission electron microscopy (TEM) further revealed that, the microtubule structures exhibited in the trunk of cellular protrusion (Figure 2H). These findings suggest that the morphological features of hiPSCs‐differentiated cells, are closely resembled with the neuronal neurite outgrowth in the early stage of neuronal development, thereby raising the possibility of hiPSCs‐derived neurons may serve as a valuable human neuronal model for the study of human neuronal cytoskeleton development.

To further characterize the neuronal properties of SMP‐induced, hiPSCs‐differentiated cells, the formation of the axon initial segment (AIS) was examined in the differentiated cells at 3 DIV [50], using immunofluorescence staining with ankyrin‐G (the AIS master scaffolding protein) and the AIS‐specific channel proteins, SCN8A (Nav1.6 sodium channel protein) and KCNQ2 (Kv7 potassium channel protein). The results revealed obvious ankyrin G‐positive segments exhibiting a characteristic tapered morphology in the proximal axon (Figure S4C,D). Importantly, these AIS structures showed clear colocalization with both SCN8A and KCNQ2, validating the proper assembly of this critical subdomain, and the recruitment of key voltage‐gated ion channels, which are essential for action potential (AP) initiation and regulation. Furthermore, we examined action potential patterns to determine the functional maturity of hiPSCs‐derived neurons in the process of SMP‐induced hiPSCs differentiation. Robustly, four types of action potential patterns were observed in the process of SMP‐induced hiPSCs differentiation, including type_A (no action potential), type_B (a single action potential), type_C (immature multiple action potentials), and type_D (mature multiple action potentials) (Figure 2I). These AP patterns represent a spectrum of neuronal electrophysiological maturity, ranging from low to high. Notably, type_B cells emerged as early as the third day of the SMP‐induced differentiation, while type_D mature neurons appeared at the eighth day (Figure 2J). By day 30, the proportion of type_D mature neurons increased significantly to ∼91.67% (n = 36 cells) of total recorded cells, indicating that the majority of the hiPSCs‐differentiated cells were functional mature neurons.

A neuronal action potential mainly involves three phases, including depolarization, repolarization, and hyperpolarization (Figure 2K). Human neurons exhibit a diverse array of ion channels, such as sodium channels, potassium channels, calcium channels, etc. [98, 99] The assembly of these ion channels is closely linked to the functional maturation of neuronal development. In fact, results from the aforementioned RNA‐seq analysis revealed a significant upregulation of various ion channel genes in the process of SMP‐induced hiPSCs differentiation (Figure S3C, and Figure 2L), in which the upregulated genes included those encoding voltage‐gated sodium channels (involving in AP depolarization), potassium channels (involving in AP repolarization and hyperpolarization), calcium channels, as well as chloride channels and Na^+^/K^+^‐transporting ATPase, etc. These results indicate that the functional characteristics of hiPSCs‐differentiated cells also closely resemble the functional maturity of human neurons, further validating that the SMP‐induced, hiPSCs‐differentiated neurons may serve as a valuable human neuronal model for the study of human neuronal functional mature development.

Besides, we examined the formation of synapse‐like structures and neural networks of hiPSCs‐derived neurons in the process of SMP‐induced hiPSCs differentiation. Images of TEM showed the physical contacts between neuronal protrusions and cell bodies, with multiple vesicles in the gap (magnification of the image), thereby validating the synapse‐like connections between the two cells (Figure 2M). Also, Immunofluorescence staining revealed that, nearly all hiPSCs‐derived neurons formed complex neural networks, and expressed the presynaptic marker synaptophysin (SYP), the postsynaptic marker PSD95, and the mature neurons marker MAP2, at day 30 of SMP‐induced hiPSCs differentiation (Figure 2N). Consistently, a significant increase of both *SYP* gene (encoding SYP protein, increased ∼12‐fold, *p*<0.0069, vs. SMP_d0), and *DLG4* gene (encoding PSD95 protein, increased ∼4.7‐fold, *p* = 0.0007, vs. SMP_d0), was found in hiPSCs‐derived neurons at day 3 of SMP‐induced differentiation, by using real‐time quantitative PCR (RT‐qPCR) analysis (Figure S4E,F). In addition, spontaneous excitatory postsynaptic currents (sEPSCs) were detected in ∼16.7% of the hiPSCs‐derived neurons (n = 36 cells) at day 30 of SMP‐induced differentiation, by using whole‐cell patch‐clamp recording (Figure 2O), further validating the formation of functional synaptic connections between the two different cells. Quantitative analysis revealed sEPSCs in SMP‐induced, hiPSCs‐differentiated neurons with an average amplitude of ‐85.8 ± 8.8 pA (*p* = 0.0002, vs. vehicle, Figure 2P), and frequency of 3.1 ± 0.4 Hz (*p* = 0.0005, vs. vehicle, Figure 2Q), whereas no sEPSCs were detected in vehicle‐treated cells (Figure 2O–Q). In contrast to the excitatory synaptic activity, no spontaneous inhibitory postsynaptic currents (sIPSCs) were detected in either SMP‐ or vehicle‐treated cells at 30 DIV (n = 15 cells per group, Figure S4G). This absence of inhibitory synaptic transmission is consistent with the predominantly glutamatergic composition of the differentiated cells (Figure 1F,G), which limits the formation of functional inhibitory circuits in the present model. Moreover, using Fluo‐4 fluorescence probes to detect the intracellular calcium influx, we found that compared to the cells cultured with vehicle medium at 30 DIV (vehicle_d30, Video S2‐1), the SMP‐induced, hiPSCs‐derived neurons at 30 DIV (SMP_d30) exhibited spontaneous calcium spikes with synchronized neural network activity (Figure S4H–K, Video S2‐2). The peak calcium fluorescence intensity (in ΔF/F0) and calcium transient frequency (in Hz) of recorded cells in the SMP_d30 group, were significantly higher than those in the vehicle_d30 group (peak: 15.3±2.6 vs. 2.2±1.2, *p* = 0.0018; frequency: 0.039±0.006 vs. 0.003±0.002, *p* = 0.0005, SMP_d30 group vs. vehicle_d30 group) (Figure S4L,M), verifying the formation of functional neural network at the late stage of the SMP‐induced hiPSCs differentiation. To further assess functional maturation at the neural network level, microelectrode array (MEA) recordings were performed on the hiPSCs‐derived neurons at 60 DIV of SMP‐induced differentiation. The SMP‐induced, hiPSCs‐differentiated neurons exhibited substantially stronger neural network activity compared to the vehicle control (Figure S4N,O), with a significantly higher average firing rate (4.5±0.7 vs. 0±0, *p* = 0.0017, SMP_d60 group vs. vehicle_d60 group, Figure S4P). Spectral analysis of local field potentials (LFPs) revealed neural oscillatory activity in the SMP group across multiple frequency bands, including theta (4–8 Hz), alpha (9–12 Hz), beta (13–30 Hz), and gamma (31–100 Hz) oscillatory rhythms, which was largely absent in vehicle‐treated cultures (Figure S4Q‐T), attesting the formation of functional neural networks in the late stage of the SMP‐induced hiPSCs differentiation.

Altogether, these findings suggest that the SMP‐induced, hiPSCs‐differentiated neurons can be used as an in vitro human neuronal model for the study of human neural development, as inferred by neuronal cytoskeleton development, electrophysiological functional maturation, and neural network formation.

### 2.3 Exploring the Potential Mechanisms Underlying Human Neuronal Morphological Development Using the SMP‐Induced, hiPSCs‐Differentiated Neuronal Model

**FIGURE 3:**
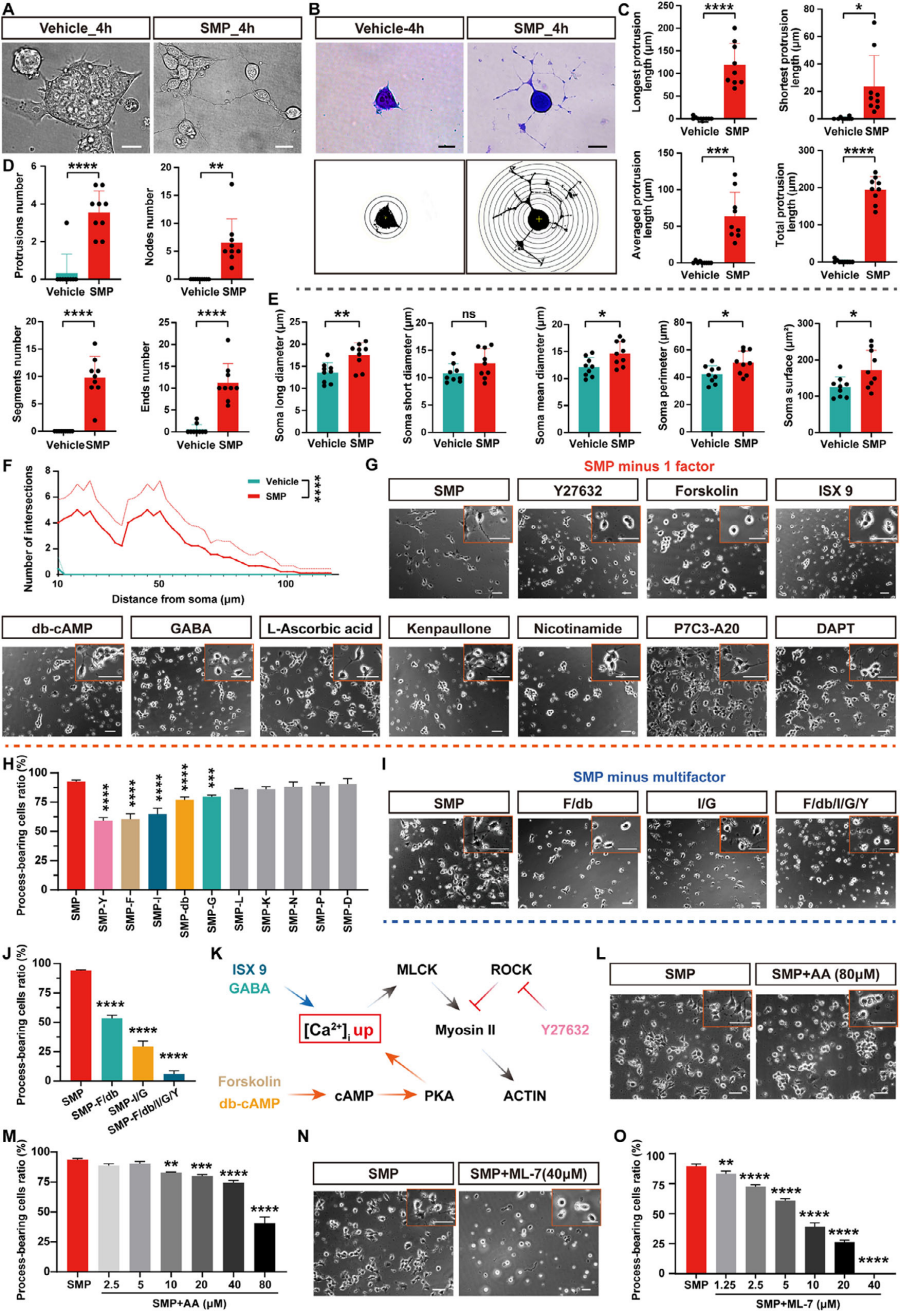
Exploring the potential mechanisms underlying human neuronal morphological development using the SMP‐induced, hiPSCs‐differentiated neuronal model. (A) Representative high‐power phase‐contrast images show alterations of cell morphology in hiPSCs‐differentiated cells cultured with the SMP or vehicle for 4 h. Scale bars = 20 µm. (B) Representative images showing the Coomassie blue staining of hiPSCs‐differentiated cells cultured with the SMP or vehicle for 4 h. Cell outlines processed by using ImageJ software are shown in the bottom (n = 9 cells per group). Scale bars = 10 µm. (C) Bar graphs show the assessment of the cellular protrusion length, including the longest, the shortest, the averaged, and the total protrusion length, for hiPSCs‐differentiated cells in the SMP_4h and Vehicle_4h groups (n = 9 cells per group). (D) Bar graphs show the assessment of the cellular protrusion complexity, including the number of protrusions, nodes, segments, and the end numbers of the cellular protrusions, for hiPSCs‐differentiated cells in the SMP_4h and Vehicle_4h groups (n = 9 cells per group). (E) Bar graphs show the assessment of the cellular soma parameters, including long/short diameter, averaged diameter, perimeter, and area of the cellular soma, for hiPSCs‐differentiated cells in the SMP_4h and Vehicle_4h groups (n = 9 cells per group). (F) Line graphs show the Sholl analysis of hiPSCs‐differentiated cells in the SMP_4h and Vehicle_4h groups (n = 9 cells per group). Solid lines represent the mean values, and dashed lines indicate the standard error. (G) Representative phase‐contrast images showing changes of cell morphology in hiPSCs‐differentiated cells cultured with the “SMP minus‐1 factor” medium. Scale bar = 50 µm. Insets in the upper right corner showing higher magnification images, scale bar = 50 µm. (H) Bar graph showing the percentage of process‐bearing cells after 4 h differentiating from hiPSCs with the “SMP minus‐1 factor” medium (n = 3 biological replicates per group). (I) Representative phase‐contrast images showing changes of cell morphology in hiPSCs‐differentiated cells cultured with the “SMP minus multifactor” medium. Scale bar = 50 µm. Insets in the upper right corner showing higher magnification images, scale bar = 50 µm. (J) Bar graph shows the percentage of process‐bearing cells after 4 h differentiating from hiPSCs with the “SMP minus multifactor” medium (n = 3 biological replicates per group). (K) Schematic diagram illustrating the proposed small molecule compounds and the potential mechanism underlying rapid neuronal cytoskeleton development in the process of SMP‐induced hiPSCs differentiation. (L) Representative phase‐contrast images showing changes of cell morphology in hiPSCs‐differentiated cells cultured with the SMP medium containing arachidonic acid (80 µM) for 4 h. Scale bar = 50 µm. Insets in the upper right corner showing higher magnification images, scale bar = 50 µm. (M) Bar graph showing the percentage of process‐bearing cells after 4 h differentiating from hiPSCs with the SMP medium containing various concentrations of arachidonic acid (n = 3 biological replicates per group). (N) Representative phase‐contrast images showing changes of cell morphology in hiPSCs‐differentiated cells cultured with the SMP medium containing ML‐7 (40 µM) for 4 h. Scale bar = 50 µm. Insets in the upper right corner showing higher magnification images, scale bar = 50 µm. (O) Bar graph showing the percentage of process‐bearing cells after 4 h differentiating from hiPSCs with the SMP medium containing various concentrations of ML‐7 (n = 3 biological replicates per group). Data are presented as mean ± SEM. ^*^ *p*<0.05; ^**^ *p*<0.01; ^***^ *p*< 0.001; ^****^ *p*< 0.0001; ns., not significant. Two‐tailed unpaired *t* test for (D, upper left), and (E); two‐tailed unpaired *t* test with Welch's correction for (C) and (D, others); repeated‐measures two‐way ANOVA with Sidak's *post‐hoc* test for (F); one‐way ANOVA with Dunnett's *post‐hoc* test for (H), (J), (M), and (O).

Based on the development of the in vitro human neuronal model derived from the SMP‐induced, hiPSCs‐differentiated neurons, we began to explored the potential mechanisms underlying human neuronal morphological development. First, we investigated the neuronal cytoskeleton development by examining the cellular protrusion morphology in the process of SMP‐induced hiPSCs differentiation. We found that after 4 h SMP inducing, the hiPSCs‐differentiated cells exhibited typical process‑bearing cellular morphology, with long cellular protrusions as compared to the controls (Figure 3A). Using both Coomassie brilliant blue staining for visualizing cellular cytoskeleton and single‐cell morphological analysis (Figure 3B), we found that the cellular protrusion length, including the longest protrusion length, the shortest protrusion length, the averaged protrusion length, and the total protrusion length, were significantly increased in the SMP_4h‐induced cells relative to the vehicle controls (Figure 3C). Also, the cellular protrusion complexity, inferred by the protrusion numbers, the node numbers, the segment numbers, and the end numbers of the cellular protrusions, were substantially increased in the SMP_4h‐induced cells in contrast to the vehicle controls (Figure 3D). Except for the short diameter of cellular soma, other parameters of cellular soma size, including long diameter, averaged diameter, perimeter, and area of the cellular soma, exhibited slight increases in the SMP_4h‐induced cells compared to the vehicle controls (Figure 3E). Furthermore, Sholl analysis also identified the complexity of the cellular protrusions in the SMP_4h‐induced cells (Figure 3F). Collectively, these findings indicate that the SMP‐induced, hiPSCs‐differentiated cells exhibited rapid cytoskeleton development, as inferred by the increases of the cellular protrusion length, the complexity of cellular protrusion arborization, and the size of cellular soma, in the early stage (4 h) of the of SMP‐induced hiPSCs differentiation.

To further identify the key factors involved in the rapid cellular cytoskeleton development, we examined which component(s) in the SMP combination is (are) required for neuronal conversion and cellular protrusion growth in the process of SMP‐induced hiPSCs differentiation. A strategy of removing one factor per trial from the SMP combination was performed, to investigate the effect of “SMP minus‐1 factor” on the differentiation of process‐bearing cells from hiPSCs (Figure 3G). The results showed that compared to the SMP control, the proportions of differentiated process‐bearing cells were significantly reduced, with the removal of Y27632 (59.2±1.5%, *p*<0.0001), forskolin (60.6±2.7%, *p*<0.0001), ISX‐9 (65.0±2.8%, *p*<0.0001), db‐cAMP (77.0±1.5%, *p*<0.0001), and GABA (79.6±0.9%, *p* = 0.0005), but not L‐ascorbic acid (86.1±0.4%, *p* = 0.1262), kenpaullone (86.3±1.2%, *p* = 0.1450), nicotinamide (88.3±2.2%, *p* = 0.5190), P7C3‐A20 (89.2±1.4%, *p* = 0.7512), and DAPT (91.2±2.7%, *P* = 0.9973), respectively from the SMP combination (Figure 3H), indicating that the five candidate small molecules, including Y27632, forskolin, ISX‐9, db‐cAMP, and GABA in the SMP combination, were likely involved in the rapid cellular cytoskeleton development. Furthermore, based on the functional similarities of the small molecules in the SMP combination, we grouped these five candidate small molecules into the following three combinations: combination F/db (forskolin and db‐cAMP; both acting through cAMP signaling pathway) [100, 101], combination I/G (ISX‐9 and GABA; both elevating intracellular calcium levels) [102, 103], and combination F/db/I/G/Y (all five candidate small molecules). By using a “SMP minus multifactor” strategy, that is a strategy of removing multifactor per trial from the SMP combination (Figure 3I), we found that the proportion of differentiated process‐bearing cells were significant decreased in the SMP‐F/db group (53.5±1.5%, *p*<0.0001), the SMP‐I/G group (29.4±2.6% of control, *p*<0.0001), and in the SMP‐F/db/I/G/Y group (6.1±1.6% of control, *p*<0.0001), and also, the decreased process‐bearing cells ratio was much more significant in the SMP‐F/db/I/G/Y group than that in the SMP‐F/db group and the SMP‐I/G group, respectively (Figure 3J). These data indicate that all of the five small molecules, including Y27632, forskolin, ISX‐9, db‐cAMP, and GABA, are required for rapid cellular cytoskeleton development in the process of SMP‐induced hiPSCs differentiation.

Y27632, a selective ROCK inhibitor, is seen to play a crucial role in inducing the growth of neuron‐like protrusion by suppressing myosin II and affecting cytoskeletal assembly (Figure 3K) [42, 104]. Indeed, introduction of arachidonic acid (AA), a lipid that activates ROCKs [105], into the SMP combination resulted in a dose‐dependent reduction in the proportion of differentiated process‐bearing cells, validating the involvement of ROCK signaling pathway in neuronal cytoskeleton development (Figure 3L,M). Moreover, forskolin and db‐cAMP are capable of upregulating intracellular calcium ion concentration by activating intracellular cAMP signaling [100, 106, 107, 108], while ISX 9 and GABA have similar functions to elevate intracellular calcium levels [102, 103]. We speculated that these small molecules may also contribute to rapid neuronal cytoskeleton development by increasing intracellular calcium ion concentration, which in turn activates myosin light chain kinase (MLCK) and promotes myosin II‐mediated actin assembly (Figure 3K). In fact, by adding the MLCK inhibitor ML‐7 to the SMP combination, we indeed found that the proportion of differentiated process‐bearing cells was significantly decreased in a dose‐dependent manner, with almost a complete loss observed at a concentration of 40 µM (Figure 3N,O). These data indicate that both the ROCK signaling pathway and the calcium ion‐activated MLCK signaling pathway, are likely involved in rapid neuronal cytoskeleton development in the process of SMP‐induced hiPSCs differentiation.

### 2.4 Involvement of L‐Type Voltage‐Gated Calcium Channels in Early Neuronal Morphogenesis in the Process of SMP‐Induced hiPSCs Differentiation

**FIGURE 4:**
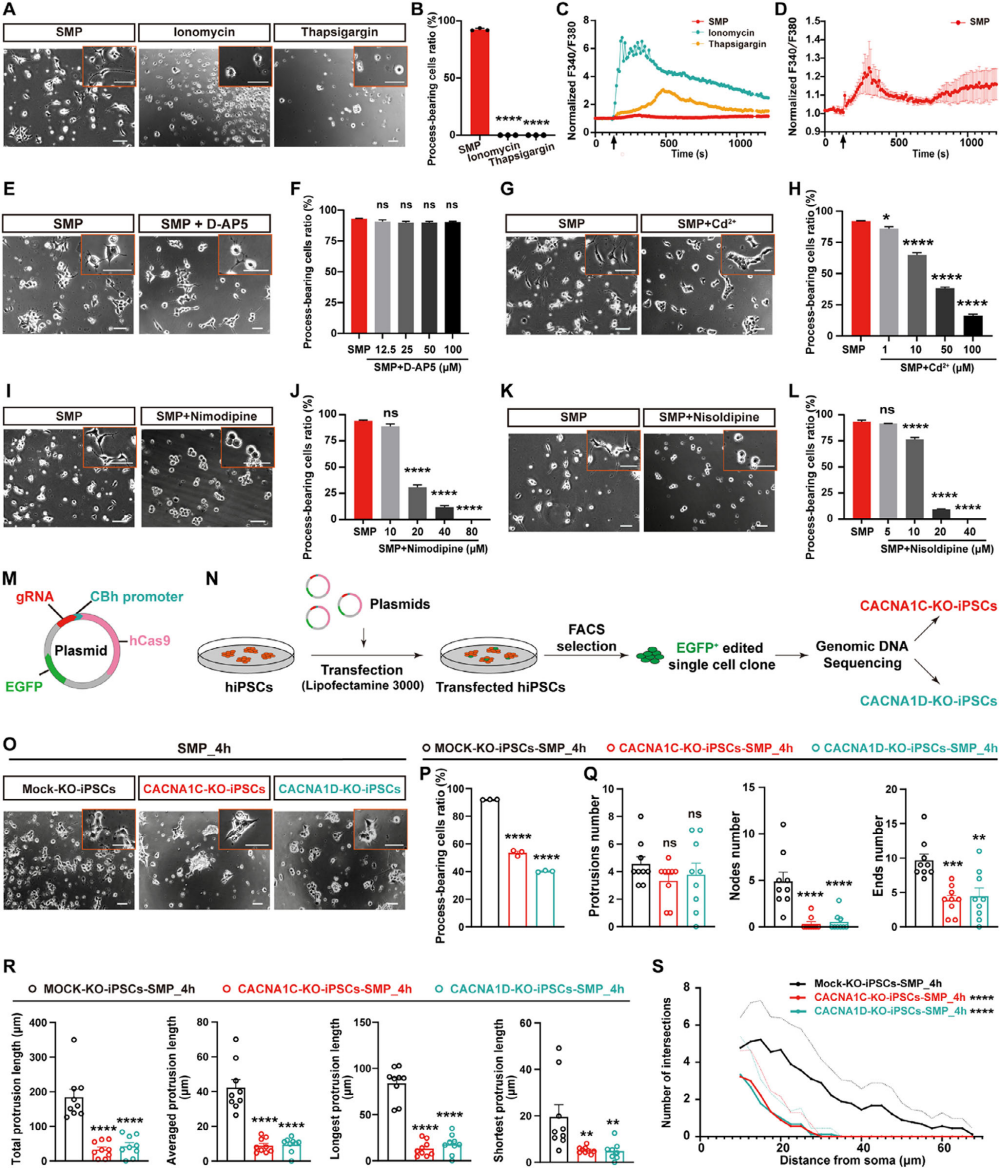
Involvement of L‐type voltage‐gated calcium channels in early neuronal morphogenesis in the process of SMP‐induced hiPSCs differentiation. (A) Representative phase‐contrast images showing changes of cell morphology in hiPSCs‐differentiated cells (for 4 h), cultured with ionomycin (1 µM), thapsigargin (1 µM), or SMP medium. Both ionomycin and thapsigargin are dissolved in neurobasal medium plus B‐27 supplement. Scale bar = 50 µm. Insets in the upper right corner showing higher magnification images, scale bar = 50 µm. (B) Bar graph showing the percentage of process‐bearing cells after 4 h differentiating from hiPSCs with ionomycin (1 µM), thapsigargin (1 µM), or SMP medium (n = 3 biological replicates per group). (C) Line graphs show the alterations of normalized intracellular calcium fluorescent signals (F340/F380) in cultured hiPSCs in response to ionomycin (1 µM), thapsigargin (1 µM), or SMP treatment (n = 10‐12 cells per group). Arrow shows the time (120 sec) of sample added. (D) Line graph showing the alterations of normalized intracellular calcium fluorescent signals (F340/F380), with enlarged Y‐axis, in cultured hiPSCs in response to SMP treatment (n = 11 cells per group). Arrow shows the time (120 sec) of sample added. (E) Representative phase‐contrast images showing changes of cell morphology in hiPSCs‐differentiated cells cultured with the SMP medium containing D‐AP5 (100 µM) for 4 h. Scale bar = 50 µm. Insets in the upper right corner showing higher magnification images, scale bar = 50 µm. (F) Bar graph showing the statistical analysis of the percentage of neuron‐like cells after 4 h differentiating from hiPSCs with the SMP medium containing various concentration of D‐AP5 (n = 3 biological replicates per group). (G) Representative phase‐contrast images showing changes of cell morphology in hiPSCs‐differentiated cells (for 4 h), cultured with the SMP medium containing Cd^2+^ (100 µM) (n = 3 biological replicates per group). Scale bars = 50 µm. Insets in the upper right corner showing higher magnification images, scale bar = 50 µm. (H) Bar graph showing the percentage of process‐bearing cells after 4 h differentiating from hiPSCs with the SMP medium containing various concentrations of Cd^2+^ (n = 3 biological replicates per group). (I) Representative phase‐contrast images showing changes of cell morphology in hiPSCs‐differentiated cells (for 4 h), cultured with the SMP medium containing nimodipine (80 µM) (n = 3 biological replicates per group). Scale bars = 50 µm. Insets in the upper right corner showing higher magnification images, scale bar = 50 µm. (J) Bar graph showing the percentage of process‐bearing cells after 4 h differentiating from hiPSCs with the SMP medium containing various concentrations of nimodipine (n = 3 biological replicates per group). (K) Representative phase‐contrast images showing changes of cell morphology in hiPSCs‐differentiated cells (for 4 h), cultured with the SMP medium containing nisoldipine (20 µM) (n = 3 biological replicates per group). Scale bars = 50 µm. Insets in the upper right corner showing higher magnification images, scale bar = 50 µm. (L) Bar graph showing the percentage of process‐bearing cells after 4 h differentiating from hiPSCs with the SMP medium containing various concentrations of nisoldipine (n = 3 biological replicates per group). (M) Schematic diagram illustrating the plasmid carrying the guide RNA (gRNA) and Cas9 protein sequences. (N) Schematic diagram shows the experimental procedure for generating monoclonal gene‐edited cell lines from plasmid‐transfected hiPSCs. (O) Representative phase‐contrast images showing changes of cell morphology in mock‐KO‐iPSCs, CACNA1C‐KO‐iPSCs and CACNA1D‐KO‐iPSCs, respectively, after 4 h culture with the SMP medium (n = 3 biological replicates per group). Scale bars = 50 µm. Insets in the upper right corner showing higher magnification images, scale bar = 50 µm. (P) Bar graph shows the percentage of process‐bearing cells after 4 h differentiating with the SMP medium from mock‐KO‐iPSCs, CACNA1C‐KO‐iPSCs, and CACNA1D‐KO‐iPSCs, respectively (n = 3 biological replicates per group). (Q) Bar graphs show the assessment of the cellular protrusion complexity, including protrusions number, nodes number, and the ends number of the cellular protrusions, for 4 h differentiated cells with the SMP medium from mock‐KO‐iPSCs, CACNA1C‐KO‐iPSCs, and CACNA1D‐KO‐iPSCs, respectively (n = 9 cells per group). (R) Bar graphs show the assessment of the cellular protrusion length, including the total protrusion length, the averaged, the longest, and the shortest protrusion length, for 4 h differentiated cells with the SMP medium from mock‐KO‐iPSCs, CACNA1C‐KO‐iPSCs, and CACNA1D‐KO‐iPSCs, respectively (n = 9 cells per group). (S) Line graphs show the Sholl analysis of hiPSCs‐differentiated cells from mock‐KO‐iPSCs, CACNA1C‐KO‐iPSCs, and CACNA1D‐KO‐iPSCs, respectively, after 4 h culture with the SMP medium (n = 9 cells per group). Solid lines represent the mean values, and dashed lines indicate the standard error. Data are presented as mean ± SEM. ^*^ *p*<0.05; ^**^ *p*<0.01; ^***^ *p*< 0.001; ^****^ *p*< 0.0001; ns., not significant. One‐way ANOVA with Dunnett's *post‐hoc* test for (B), (F), (H), (J), (L), and (P‐R); Repeated‐measures two‐way ANOVA with Sidak's *post‐hoc* test for (S). See also Figure S5 and S6.

To further determine whether the elevated intracellular calcium ion level underlies the rapid neuronal cytoskeleton development in the process of SMP‐induced hiPSCs differentiation, we first examined whether merely increased intracellular calcium ion level, by ionomycin (a calcium ionophore that facilitates calcium influx) or thapsigargin (a Ca^2^^+^‐ATPase inhibitor that reduces calcium uptake by the endoplasmic reticulum), could induce the conversion of hiPSCs into neurons. Surprisingly, both ionomycin (1 µM, dissolved in neurobasal medium plus B‐27 supplement) and thapsigargin (1 µM, dissolved in neurobasal medium plus B‐27 supplement) treatment (for 4 h) resulted in a vast of cell death but not the conversion of process‐bearing cells from hiPSCs (Figure 4A,B). Using ratiometric calcium dye Fura‐2 AM to image calcium ion fluorescence signals, we observed that both ionomycin‐ and thapsigargin‐treatment induced rapid increases in intracellular calcium concentration of cultured hiPSCs, whereas the cells by the SMP‐treated hiPSCs exhibited a very slower increase rate of intracellular calcium levels (Figure 4C,D). We reasoned that the cell death after the treatment of ionomycin or thapsigargin, may be due to the calcium overload resulted from rapid increase of intracellular calcium concentration by ionomycin or thapsigargin. Therefore, the slower increase of intracellular calcium levels by SMP is probably a result of a distinct mechanism, which likely underlies the rapid neuronal cytoskeleton development in the process of SMP‐induced hiPSCs differentiation.

Thus, we investigated whether other receptors or ion channels, such as N‐methyl‐D‐aspartate receptors (NMDARs, a member of the ionotropic glutamate receptor family) and voltage‐gated calcium channels (VGCCs), which mediate calcium influx of neurons, contribute to the slower increase of intracellular calcium levels by SMP, and participate in the rapid neuronal morphogenesis in the process of SMP‐induced hiPSCs differentiation. Using D‐AP5 (an NMDARs antagonist) or Cd^2+^ (cadmium ions, a broad‐spectrum VGCCs inhibitor) to inhibit the activity of NMDARs and VGCCs, respectively, we found that D‐AP5 had no significant effect on the proportion of differentiated process‐bearing cells by adding to the SMP combination (Figure 4E,F). In contrast, Cd^2+^ significantly reduced the proportion of differentiated process‐bearing cells in a dose‐dependent manner, with approximately 16.4±1.1% of the control (*p*<0.0001) at a concentration of 100 µM (Figure 4G,H). To exclude the possibility that the reduced proportion of process‑bearing cells was due to the loss of total cell numbers or cytotoxicity, we examined total cell number and multiple cell toxicity indicators in the presence of different antagonists in the SMP combination. In Cd^2+^‑treatment experiments, relative cell number, TUNEL (TdT‐mediated dUTP Nick‐End Labeling) staining, MTT (3‐(4,5‐dimethylthiazol‐2‐yl)‐2,5‐diphenyltetrazolium bromide) assays, and LDH (lactate dehydrogenase) release assays showed that, total cell numbers, apoptosis level, mitochondrial activity, and membrane integrity, were all comparable to those of the SMP control (no significant difference, SMP+Cd^2+^ vs. SMP, Figure S5A–E), indicating no significant loss of total cells or cytotoxicity occurrence, thereby the reduced proportion of process‑bearing cells upon Cd^2+^‑mediated blockade of VGCCs, likely resulting from a reduction of neuronal conversion efficiency. These data demonstrate that VGCCs play a key role in rapid neuronal morphological development in the process of SMP‐induced hiPSCs differentiation.

To further determine which subtypes of the VGCCs are involved in rapid neuronal morphological development in the process of SMP‐induced hiPSCs differentiation, we examined the effects of different subtypes of VGCCs antagonists on the conversion of process‐bearing cells during SMP‐induced hiPSCs differentiation. We found that using nimodipine or nisoldipine, the two antagonists of L‐type calcium channels to block the activity of the channels, significantly inhibited the conversion of process‐bearing cells in a dose‐dependent manner, e.g., the proportion of differentiated process‐bearing cells reduced to almost 0% in the presence of 80 µM nimodipine (*p*<0.0001, vs. SMP, Figure 4I,J) and 40 µM nisoldipine (*p*<0.0001, vs. SMP, Figure 4K,L), respectively. Similarly, in cultures treated with nimodipine or nisoldipine, assays of relative cell number, TUNEL staining, MTT assays, and LDH release showed that all parameters were comparable to those of the SMP control (no significant difference, SMP+nimodipine vs. SMP or SMP+nisoldipine vs. SMP, Figure S5F–J for nimodipine; Figure S5K–O for nisoldipine), indicating no significant loss of total cells or cytotoxicity occurrence, thereby the reduced proportion of process‑bearing cells upon L‑type VGCC blockade, likely resulting from a reduction of neuronal conversion efficiency. In contrast, no significant alteration was found in the proportion of differentiated process‐bearing cells, by adding either ω‐conotoxin MVIIC (an antagonist of P/Q and N‐type calcium channels, at 1, 2, and 4 µM concentration), SNX‐482 (a selective antagonist of R‐type calcium channels, at 0.5, 1, and 2 µM concentration), or ethosuximide (a selective antagonist of T‐type calcium channels, at 20, 40, and 80 µM concentration) to the SMP combination (Figure S5P–U). These results indicate that L‐type voltage‐gated calcium channels play a vital role in early neuronal morphogenesis, in the process of SMP‐induced hiPSCs differentiation.

Based on the aforementioned RNA‐seq data (Figure S3C), there are four subtypes of L‐type voltage‐gated calcium channels expressed in hiPSCs, including Ca_v_1.1 (encoded by *CACNA1S*), Ca_v_1.2 (encoded by *CACNA1C*), Ca_v_1.3 (encoded by *CACNA1D*), and Ca_v_1.4 (encoded by *CACNA1F*), of which, the expression of both *CACNA1C* (encoding Ca_v_1.2) mRNA level and *CACNA1D* (encoding Ca_v_1.3) mRNA level was relatively higher compared to the other subtypes (Figure S5V). It is reported that excitatory stimuli can directly act on neural progenitor cells (NPCs) to mediate neurogenesis through Ca_v_1.2/Ca_v_1.3 [43, 109, 110]. We thus speculated that Ca_v_1.2 and Ca_v_1.3 may play a critical role in early neuronal morphogenesis in the process of SMP‐induced hiPSCs differentiation. To test this idea, we first examined whether knockdown of *CACNA1C* (encoding Ca_v_1.2) or *CACNA1D* (encoding Ca_v_1.3) in hiPSCs could disrupt the neuronal morphogenesis in the process of SMP‐induced hiPSCs differentiation. Recombinant lentivirus expressing either *CACNA1C* short hairpin RNA (shRNA) linked with mCherry (LV‐shCACNA1C), or *CACNA1D* shRNA linked with gcGFP (LV‐shCACNA1D), was utilized to infect hiPSCs and knock down CACNA1C and CACNA1D expression in hiPSCs, respectively (Figure S6A‐E). Unexpectedly, no significant knockdown effects were observed on CACNA1C and CACNA1D expression in hiPSCs, either by LV‐shCACNA1C or LV‐shCACNA1D treatment (Figure S6F,G). Consistently, neither LV‐shCACNA1C nor LV‐shCACNA1D affected the neuronal morphogenesis in the early stage (4 h) of SMP‐induced hiPSCs differentiation (Figure S6H‐K). We speculated that the failure of knocking down CACNA1C and CACNA1D expression in hiPSCs, by either LV‐shCACNA1C or LV‐shCACNA1D, may be due to the lengthy mRNA sequences and low expression levels of *CACNA1C* and *CACNA1D* in hiPSCs.

Then, we employed another strategy to generate *CACNA1C* or *CACNA1D* knockout (KO) cells in hiPSCs by CRISPR‐Cas9 system (Figure 4M,N). Knockout of either *CACNA1C* or *CACNA1D* in hiPSCs was performed by Cas9 protein and guide RNA (gRNA) targeting the second exon regions of *CACNA1C* (Figure S6L) and *CACNA1D* (Figure S6M) genes, respectively. The clones of CACNA1C‐KO‐iPSCs and CACNA1D‐KO‐iPSCs that harbored mutations in exon 2 of either *CACNA1C* (Figure S6N) or *CACNA1D* (Figure s6O) genes, leading to the loss of respective protein expression in the targeted genes (Figure S6P,Q). We hence examined the effects of either *CACNA1C*‐KO or *CACNA1D*‐KO on neuronal morphogenesis in the early stage (4 h) of SMP‐induced hiPSCs differentiation. Expectedly, knockout of either *CACNA1C* gene or *CACNA1D* gene in hiPSCs, robustly impaired the neuronal morphogenesis in the process of SMP‐induced hiPSCs differentiation (Figure 4O). The proportion of differentiated process‐bearing cells reduced significantly from 92.0±0.07% in mock‐KO group, to 53.5±1.2% in CACNA1C‐KO group (*p*<0.0001, vs. mock‐KO) and 40.2±0.3% in CACNA1D‐KO group (*p*<0.0001, vs. mock‐KO), respectively, in the process of SMP_4h‐induced hiPSCs differentiation (Figure 4P). Moreover, the cellular protrusion length and complexity, inferred by the node numbers and the end numbers of the cellular protrusions, as well as by the total protrusion length, the averaged protrusion length, the longest protrusion length, and the shortest protrusion length, were significantly decreased in both CACNA1C‐KO group and CACNA1D‐KO group compared to mock‐KO group (Figure 4Q,R). Similarly, Sholl analysis further validated that the complexity of protrusion morphology was significantly lower in both CACNA1C‐KO group and CACNA1D‐KO group compared to the mock‐KO controls (Figure 4S). Together these data with the aforementioned findings, we suggest that both of Ca_v_1.2 (encoded by *CACNA1C*) and Ca_v_1.3 (encoded by *CACNA1D*), the two subtypes of L‐type voltage‐gated calcium channels, play essential roles in early morphogenesis of human neuronal development.

### 2.5 Exploring the Potential Mechanisms Underlying Human Neuronal Functional Developmental Maturation Using the SMP‐Induced, hiPSCs‐Differentiated Neuronal Model

**FIGURE 5:**
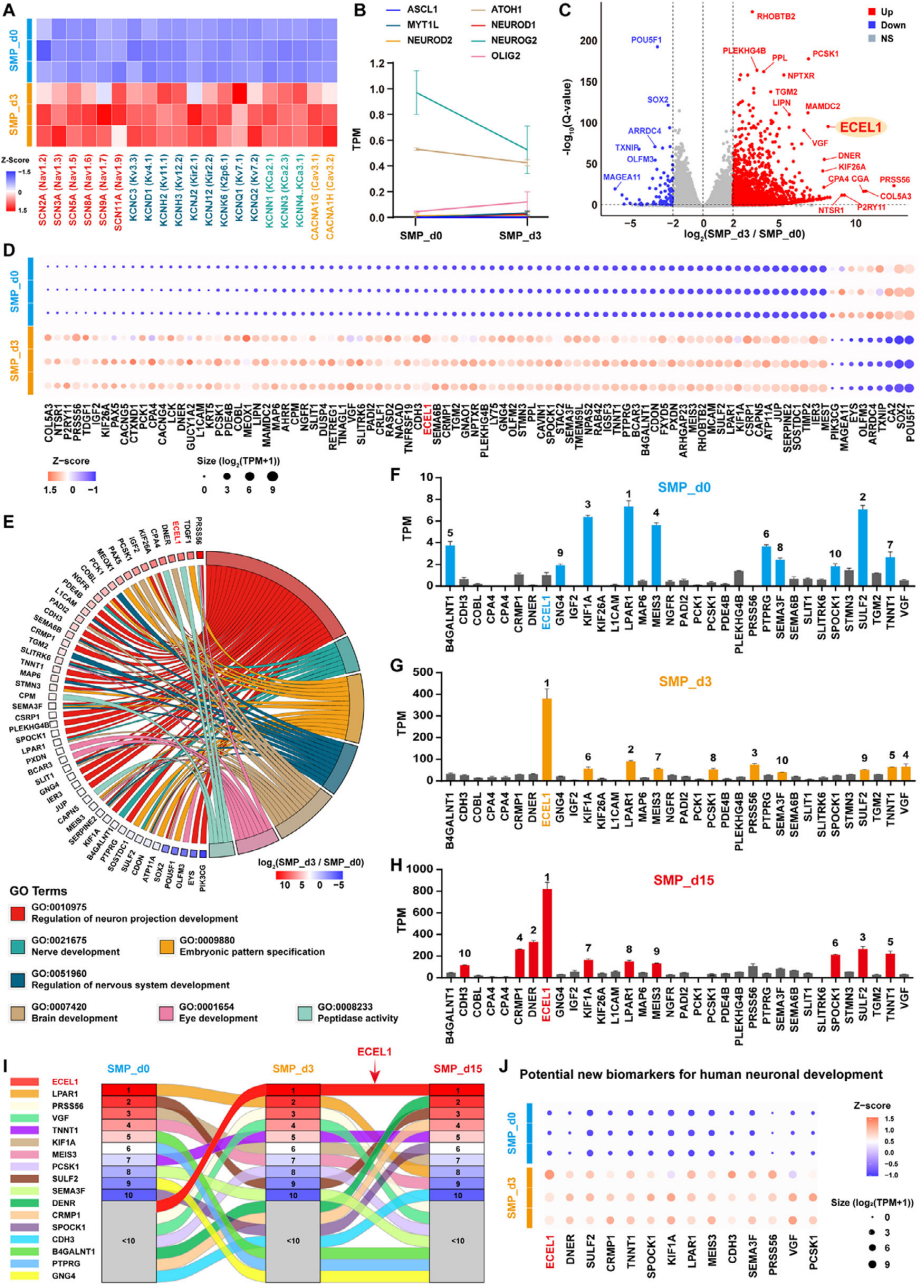
Identification of key regulators of human neuronal functional developmental maturation using RNA‐seq analysis. (A) Heatmap showing the normalized gene expression levels of multiple ion channel genes closely associated with neuronal functional maturation, by RNA‐seq analysis for hiPSCs‐differentiated cells at 0 day in vitro (DIV) and 3 DIV culture using the SMP protocol, respectively (n = 3 biological replicates per group). Z‐score normalization was performed along the columns. (B) Line graph showing changes in the expression levels of neural‐fate master genes in 3 days SMP‐induced, hiPSCs‐differentiated cells (the SMP_d3 group) compared to the SMP_d0 group (n = 3 biological replicates per group). TPM, transcripts per kilobase per million mapped reads. (C) Volcano plot showing the differentially expressed genes (DEGs) compared between the SMP_d3 group and the SMP_d0 group (n = 3 biological replicates per group). Significantly up‐regulated genes are shown as red dots, whereas down‐regulated genes are shown as blue dots, and no significant changed genes are indicated as gray. Vertical dotted lines (black) represent log_2_(fold change) = ±2, and the horizontal dotted line (black) represents Q = 0.05. (D) Bubble plot showing the DEGs compared between the SMP_d3 group and the SMP_d0 group (n = 3 biological replicates per group). Bubble size corresponds to Log_2_(TPM+1) values, and bubble color reflects Z‐score normalization performed across columns. (E) Chord diagram showing the gene ontology (GO) enrichment analysis of hiPSCs‐differentiated cells between the SMP_d3 and SMP_d0 groups (n = 3 biological replicates per group). Out of the 92 genes analyzed in (D), 51 DEGs are significantly enriched across 7 GO terms, ranked by log_2_(SMP_d3/SMP_d0) in expression. (F‐H) Bar graphs show the expression levels of the top 34 DEGs in the SMP_d0 (F), SMP_d3 (G), and SMP_d15 (H) groups (n = 3 biological replicates per group). Data are presented as mean ± SEM. (I) Sankey diagram depicting dynamic changes in gene expression. Genes ranking in the top ten for expression across three groups (SMP_d0, SMP_d3, and SMP_d15) are involved in dynamic sorting. (J) Bubble plot shows the gene expression levels for potential new biomarkers in human neuronal functional maturation development between the SMP_d3 and SMP_d0 groups (n = 3 biological replicates per group). Bubble size corresponds to Log_2_(TPM+1) values, and bubble color reflects Z‐score normalization performed across columns.

Based on the rapid functional mature of hiPSCs‐differentiated neurons, inferred by the emergence of type_B neurons at 3 DIV of SMP‐induced hiPSCs differentiation (Figure 2I,J), we then explored the potential mechanisms underlying human neuronal functional developmental maturation using the SMP‐induced, hiPSCs‐differentiated neuronal model. As aforementioned RNA‐seq analysis revealed that, the expression of multiple ion channel genes closely associated with neuronal functional maturation, was rapidly upregulated at 3 DIV of SMP‐induced hiPSCs differentiation (Figure S3C and Figure 5A), indicating the presence of potential mechanisms regulating the functional maturation of human neurons in the early stage of human neuronal development. Several neural‐fate master genes, such as *ASCL1*, *ATOH1*, *MYT1L*, *NEUROD1*, *NEUROD2*, *NEUROG2*, and *OLIG2*, have been reported to regulate the early transition of neuronal fate [66, 111, 112, 113, 114]. Surprisingly, the results from RNA‐seq analysis showed that, the expression levels of these neural‐fate master genes, presented as transcripts per kilobase per million mapped reads (TPM), did not exhibit significant alterations, and remained at low expression levels (TPM < 1) at day 3 after SMP‐induced hiPSCs differentiation (Figure 5B), indicating that other distinct mechanisms are probably involved in the early stage of human neuronal functional developmental maturation. Given that lots of hiPSCs‐differentiated neurons exhibited functional electrophysiological characteristics at 3 DIV of SMP‐induced hiPSCs differentiation (Figure 2I,J), we hence examined the differentially expressed genes (DEGs) between the SMP_d3 group and the SMP_d0 group, from the aforementioned RNA‐seq data, to identify the genes associated with human neuronal functional developmental maturation (Figure 5C). First, 92 DEGs were selected based on their expression levels and statistically significant differences. Among them, the expression levels of most DEGs were upregulated in the SMP_d3 group compared to the SMP_d0 group, while a few DEGs were downregulated (Figure 5D). Next, GO enrichment analysis was performed on the selected DEGs to identify gene clusters associated with neuronal development, and the chord diagram revealed that among the 92 DEGs, 51 DEGs were significantly enriched across 7 GO terms, with the terms ranked by their expression levels (Figure 5E). Then, the 34 most probable DEGs were selected for further analysis, based on their higher expression levels and corresponding GO terms for neuronal development (Figure 5F). Considering that the gene expression levels influence gene function, we ranked the expression levels of the 34 most probable DEGs in the SMP_d0 (Figure 5F), SMP_d3 (Figure 5G), and SMP_d15 (Figure 5H) groups. The intersection of the top 10 DEGs across the three groups identified a total of 17 DEGs, as illustrated by the Sankey diagram analysis (Figure 5I), which highlights the ranking changes of each DEG across the three groups. The results revealed that the ranks of 14 DEGs, including *ECEL1*, *DNER*, *SULF2*, *CRMP1*, *TNNT1*, *SPOCK1*, *KIF1A*, *LPAR1*, *MEIS3*, *CDH3*, *SEMA3F*, *PRSS56*, *VGF*, *and PCSK1* were consistently increased, from SMP_d0 to SMP_d3 and then to SMP_d15, in the process of SMP‐induced hiPSCs differentiation, in which the *ECEL1* gene showed the most notable rise and consistently held the top position in both of the SMP_d3 group and the SMP_d15 group (Figure 5I,J). Taken together, 14 DEGs, which exhibited significant upregulation at 3 DIV of SMP‐induced hiPSCs differentiation, were identified as potential new biomarkers for human neuronal development. Thus, these DEGs may serve as key regulators of human neuronal functional developmental maturation.

**FIGURE 6:**
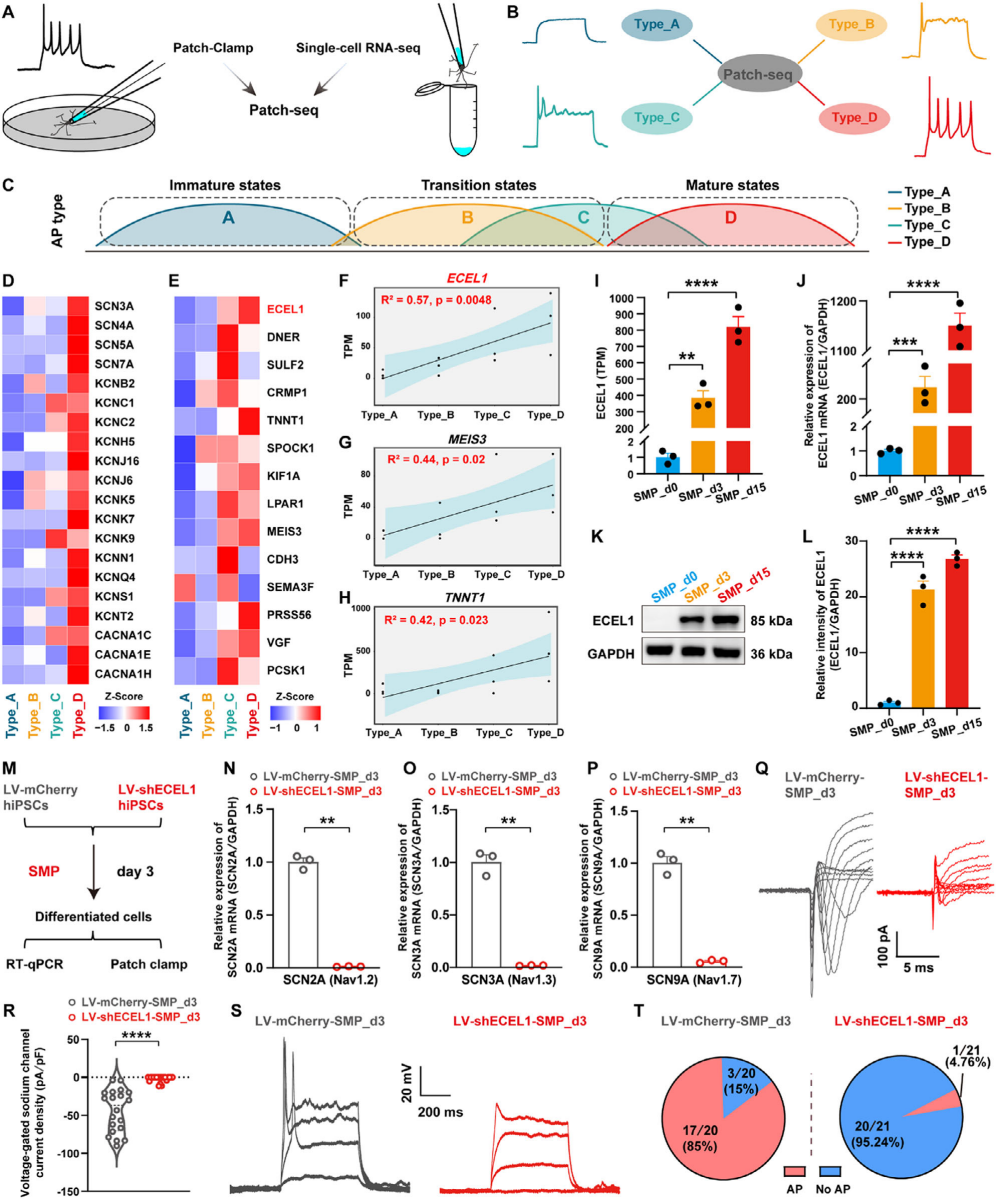
Identification of ECEL1 as a key regulator of human neuronal functional developmental maturation using Patch‐seq analysis. (A) Schematic diagram depicting the experimental procedure of Patch‐seq analysis. (B) Representative traces depicting the four types of action potential (AP) patterns (Type_A, Type_B, Type_C, and Type_D) using for Patch‐seq analysis. (C) Schematic diagram illustrating the AP patterns (Type_A, Type_B, Type_C, and Type_D) corresponding to the levels of neuronal functional maturity (immature states, transition states, and mature states). (D) Heatmap showing the normalized gene expression levels of ion channel‐associated genes across the four types of cells by Patch‐seq analysis (n = 3 biological replicates per group). Z‐score normalization was performed along the rows. (E) Heatmap showing the normalized gene expression levels of 14 potential new biomarker genes for human neuronal functional maturation development across the four types of cells by Patch‐seq analysis (n = 3 biological replicates per group). Z‐score normalization was performed along the rows. (F‐H) Correlation analysis between the gene expression level of *ECEL1* (F), *MEIS3* (G), and *TNNT1* (H) and the neuronal functional maturity (based on AP patterns) by Patch‐seq analysis (n = 3 biological replicates per group). Based on the scatter plot, a regression line was plotted, and the regression model was computed to extract R‐squared and p‐values. The regression line is colored black, and the confidence interval is filled with light blue. (I) Bar graph showing the expression level of ECEL1 gene across the SMP_d0, SMP_d3, and SMP_d15 groups by RNA‐seq analysis (n = 3 biological replicates per group). (J) Bar graph showing the expression level of ECEL1 mRNA across the SMP_d0, SMP_d3, and SMP_d15 groups by RT‐qPCR analysis (n = 3 biological replicates per group). (K‐L) Western blot analyses of ECEL1 abundance across the SMP_d0, SMP_d3, and SMP_d15 groups. (K) Representative Western blotting bands are shown. (L) Summary plot for the relative intensity of ECEL1 immunoblot (n = 3 biological replicates per group). (M) Schematic diagram illustrating the experimental procedure for investigating the effects of knocking down ECEL1 gene in hiPSCs on VGSCs genes expression (by RT‐qPCR) and electrophysiological changes (by patch clamp) of hiPSCs‐differentiated neurons at 3 days in vitro (DIV) culture using the SMP medium. (N‐P) Bar graphs showing the expression of SCN2A (N), SCN3A (O), and SCN9A (P) mRNA levels in SMP‐induced (at 3 DIV), hiPSCs‐differentiated neurons between the LV‐shECEL1 and LV‐mCherry groups (n = 3 biological replicates per group). (Q) Representative traces showing the VGSCs currents of SMP‐induced (at 3 DIV), hiPSCs‐differentiated neurons between the LV‐shECEL1 and LV‐mCherry groups. The VGSCs currents were recorded in the voltage‐clamp configuration by delivering voltage steps ranging from ‐80 mV to +60 mV for 100 ms, in 10 mV increments. Scale bar = 100 pA, 5 ms. (R) Violin plots showing the averaged current density (in pA/pF) of VGSCs in SMP‐induced (at 3 DIV), hiPSCs‐differentiated neurons between the LV‐shECEL1 and LV‐mCherry groups (n = 20‐21 cells per group). The VGSCs currents were measured at ‐20 mV holding voltage. (S) Representative traces showing the evoked action potentials of SMP‐induced (at 3 DIV), hiPSCs‐differentiated neurons between the LV‐shECEL1 and LV‐mCherry groups. Action potentials were evoked by a series of depolarizing current pulses from 0 pA to +15 pA for 600 ms, in 5 pA increments. (T) Pie chart showing the proportion of action potential firing cells between the LV‐shECEL1 and LV‐mCherry groups (n = 20‐21 cells per group). Data are presented as mean ± SEM. ^**^ *p*<0.01; ^***^ *p*<0.001; ^****^ *p*< 0.0001. One‐way ANOVA with Dunnett's *post‐hoc* test for (I), (J), (L); Two‐tailed unpaired *t* test with Welch's correction for (N), (O), (P), (R). See also Figure S7 and S8.

Furthermore, we investigated which DEGs among these 14 biomarkers play crucial roles in the functional developmental maturation of SMP‐induced, hiPSCs‐differentiated neurons. A strategy of Patch‐sequencing (Patch‐seq) analysis was employed to determine the key genes related to human neurons functional developmental maturation, in which single‐cell transcriptome sequencing was performed following patch‐clamp recording of individual cell for electrophysiological profiling analysis (Figure 6A). Four types of cells were collected based on the aforementioned action potential (AP) pattern (types A, B, C, and D) of each recorded cells (Figures 2I and 6B), and three samples of each type were pooled together for the Patch‐seq analysis of each type cells. We classified these four type cells into three states as immature states, transition states, and mature states on the basis of their action potential patterns (Figure 6C). After cell collection, single‐cell cDNA libraries were obtained through reverse transcription and amplification, and qualified samples were subjected to sequencing and bioinformatics analysis (Figure S7A). The boxplots revealed that the data dispersion of Patch‐seq was consistent across the 12 samples in terms of gene expression levels (Figure S7B), and the results of principal component analysis (PCA) showed distinct clustering of the four cell types with respect to functional maturation (Figure S7C). The expressions of both ion channel‐related genes associated with action potential firing, and the 14 potential biomarker DEGs associated with human neuronal functional developmental maturation (identified by the aforementioned RNA‐seq analysis, Figure 5J), were then examined across the four type cells in the process of SMP‐induced hiPSCs differentiation. The results showed that the expression of the majority of the ion channel‐related genes and the 14 potential biomarker DEGs, were gradually increased as the cellular electrophysiological maturity progressed (Figure 6D,E), further indicating the involvement of these 14 potential candidate DEGs in human neuronal functional maturity development. In fact, results from correlation analysis between the 14 potential DEGs and cell functional types revealed, that the expression of *ECEL1*, *MEIS3*, and *TNNT1* was significantly correlated with neuronal functional maturation (inferred by AP pattern‐related cell types), with *ECEL1* (R^2^ = 0.57, *p* = 0.0048) exhibiting the strongest correlation and the highest statistical significance (Figure 6F‐H and Figure S7D‐N). *ECEL1*, a gene encoding endothelin‐converting enzyme‐like 1 (ECEL1) protein, is also called damage‐induced neuronal endopeptidase (DINE) gene in the rodent genome [115, 116]. As a neuronal peptide endopeptidase, ECEL1 plays a pivotal role in nervous system development [117, 118], and promotes neuronal and axonal regeneration in response to neuronal damage or nerve injury [119, 120]. Indeed, the average gene expression level (in TPM) of *ECEL1* was increased from 1.0±0.2 in the SMP_d0 group to 383.9±44.9 in the SMP_d3 group (*p* = 0.0022, vs. SMP_d0 group), and reached 820.4±62.9 in the SMP_d15 group (*p*<0.0001, vs. SMP_d0 group) (Figure 6I). Likewise, the abundance of *ECEL1* mRNA level was increased, from 1.0±0.1 in the SMP_d0 group to 216.3±15.4 in the SMP_d3 group (*p* = 0.003, vs. SMP_d0 group), and reaching 1150.0±25.4 in the SMP_d15 group (*p*<0.0001, vs. SMP_d0 group) (Figure 6J). Similarly, Western blotting analyses verified the increased abundance of ECEL1 protein, from 1.0±0.2 in the SMP_d0 group to 21.3±1.5 in the SMP_d3 group (*p*<0.0001, vs. SMP_d0 group), and reaching 26.8±0.7 in the SMP_d15 group (*p*<0.0001, vs. SMP_d0 group) (Figure 6K,L). These results raise the possibility that ECEL1 may serve as a key regulator of human neuronal functional developmental maturation in the early stage of SMP‐induced hiPSCs differentiation.

The neuronal functional developmental maturation is closely linked to the expression of multiple ion channels, in particular the voltage‐gated sodium channels (VGSCs), which play a critical role in action potential depolarization and influence action potential characteristics (Figure S8A) [98, 121]. Of these VGSCs, Nav1.3, Nav1.7, and Nav1.9 amplify subthreshold stimuli; Nav1.6, Nav1.7, and Nav1.8 contribute to the rising phase of the action potential [121]; while Nav1.6 and Na_v_1.2 located in the proximal axon initial segment (AIS), promotes backpropagation of action potentials to the soma, and activates voltage‐gated potassium channels that aid in neuronal repolarization [122, 123]. Based on the aforementioned RNA‐seq data showed, that the expression of *SCN2A* (encoding Na_v_1.2), *SCN3A* (encoding Na_v_1.3), *SCN8A* (encoding Na_v_1.6), *SCN9A* (encoding Na_v_1.7), and *SCN11A* (encoding Na_v_1.9), was significantly upregulated in the SMP_d3 group compared to the SMP_d0 group (Figure S8B), we proposed that the elevated ECEL1 mediates human neuronal functional developmental maturation, through promoting rapid functional assembly and expression of VGSCs in hiPSCs‐derived neurons, in the early stage of SMP‐induced hiPSCs differentiation. To test this understanding, we first investigated whether knockdown (KD) of ECEL1 in hiPSCs could disrupt the neuronal functional maturation in the process of SMP‐induced hiPSCs differentiation. Recombinant lentivirus (LV) expressing three ECEL1 short hairpin RNAs (shRNAs) linked with mCherry, including LV‐shECEL1‐1#, LV‐shECEL1‐2#, and LV‐shECEL1‐3#, were applied to infect hiPSCs and knock down ECEL1 expression in these cells, respectively (Figure S8C,D). The knockdown efficiency of lentivirus‐expressed shRNAs was validated by RT‐qPCR analysis. The results showed that the LV‐shECEL1‐1# displayed the most significant knockdown efficiency, and reduced the ECEL1 mRNA level to 10.8±1.3% of the LV‐mCherry control (*p* = 0.0085, Figure S8E). Similar results were found in Western blotting analyses, showing that the abundance of ECEL1 protein was decreased to 25.3±4.6% of the LV‐mCherry control (*p* = 0.0018, Figure S8F,G). Thus, the LV‐shECEL1‐1# was selected for the followed ECEL1 knockdown experiments. As expected, knocking down ECEL1 in hiPSCs by LV‐shECEL1‐1#, significantly downregulated the expression of *SCN2A* (decreased to ∼1.2±0.2% of control, *p =* 0.0014), *SCN3A* (decreased to ∼1.8±0.1% of control, *p =* 0.0046), and *SCN9A* (decreased to ∼5.6±0.7% of control, *p =* 0.0035) mRNA levels in 3 DIV of the SMP‐induced, hiPSCs‐differentiated cells (Figure 6M–P). Consistently, knocking down ECEL1 in hiPSCs also reduced the inward VGSCs currents in 3 DIV of the SMP‐induced, hiPSCs‐differentiated cells (Figure 6Q,R). The average current density of VGSCs evoked at ‐20 mV was decreased from ‐44.0±6.3 pA/pF in the LV‐mCherry group to ‐9±0.8 pA/pF in the LV‐shECEL1 KD group (*p*<0.0001, Figure 6R). Also, knockdown of ECEL1 in hiPSCs robustly inhibited the action potential firing of hiPSCs‐differentiated cells, with the proportion of action potential firing cells decreased from ∼85% (17/20) of total recorded cells in the LV‐mCherry group (n = 20), to ∼4.76% (1/21) of total recorded cells in the LV‐shECEL1 KD group (n = 21) (Figure 6S,T). These results indicate that knockdown of ECEL1 in hiPSCs disrupts the neuronal functional developmental maturation, in the early stage of SMP‐induced hiPSCs differentiation, by inhibiting the functional assembly and expression of multiple ion channels such as VGSCs in hiPSCs‐derived neurons.

### 2.6 Involvement of ECEL1 in Human Neuronal Functional Developmental Maturation in the Early Stage of SMP‐Induced hiPSCs Differentiation

**FIGURE 7:**
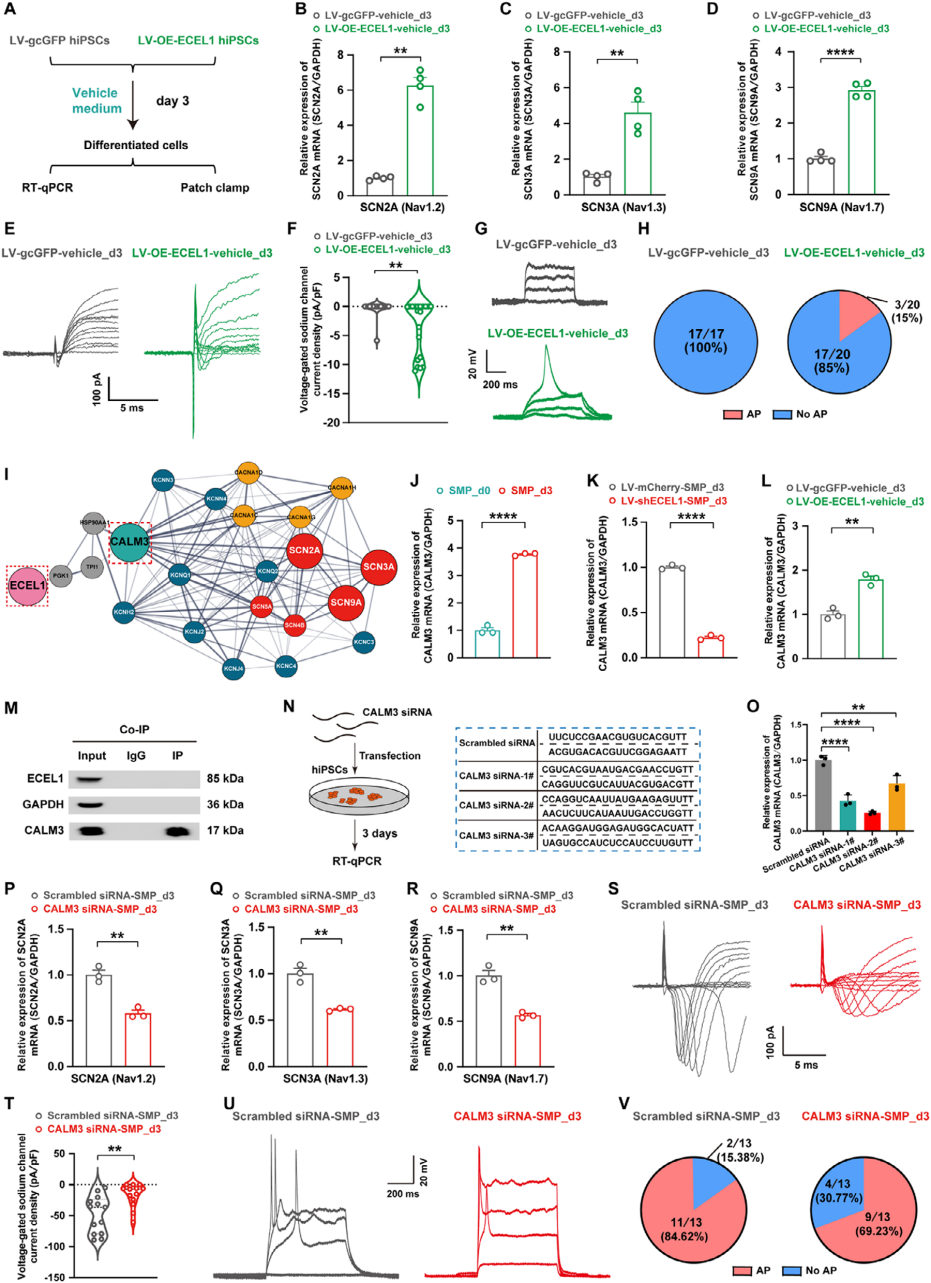
Involvement of ECEL1 in human neuronal functional developmental maturation in the early stage of SMP‐induced hiPSCs differentiation. (A) Schematic diagram illustrating the experimental procedure for investigating the effects of overexpressing ECEL1 gene in hiPSCs on VGSCs genes expression (by RT‐qPCR) and electrophysiological changes (by patch clamp) of hiPSCs‐differentiated neurons at 3 days in vitro (DIV) culture using the vehicle medium (neurobasal/B‐27 media containing 0.1% DMSO). (B‐D) Bar graphs showing the expression of SCN2A (B), SCN3A (C), and SCN9A (D) mRNA levels in hiPSCs‐differentiated neurons (3 DIV vehicle medium culture) between the LV‐OE‐ECEL1 and LV‐gcGFP groups (n = 4 biological replicates per group). (E) Representative traces showing the VGSCs currents of hiPSCs‐differentiated cells (3 DIV vehicle medium culture) between the LV‐OE‐ECEL1 and LV‐gcGFP groups. The VGSCs currents were recorded in the voltage‐clamp configuration by delivering voltage steps ranging from −80 to +60 mV for 100 ms, in 10 mV increments. Scale bar = 100 pA, 5 ms. (F) Violin plots showing the averaged current density (in pA/pF) of VGSCs in hiPSCs‐differentiated neurons (3 DIV vehicle medium culture) between the LV‐OE‐ECEL1 and LV‐gcGFP groups (n = 17–20 cells per group). The VGSCs currents were measured at ‐20 mV holding voltage. (G) Representative traces showing the evoked action potentials of hiPSCs‐differentiated neurons (3 DIV vehicle medium culture) between the LV‐OE‐ECEL1 and LV‐gcGFP groups. Action potentials were evoked by a series of depolarizing current pulses from 0 pA to +15 pA for 600 ms, in 5 pA increments. (H) Pie chart showing the proportion of action potential firing cells between the LV‐OE‐ECEL1 and LV‐gcGFP groups (n = 17‐20 cells per group). (I) Network diagram of the intersection composed of differentially expressed genes (DEGs) with ECEL1. (J) Bar graph showing the expression of CALM3 mRNA in hiPSCs‐differentiated cells at day 0 (SMP_d0) and day 3 (SMP_d3) culture with the SMP medium, respectively (n = 3 biological replicates per group). (K) Bar graph showing the expression of CALM3 mRNA between the LV‐shRNA (shRNA #1)‐ and the LV‐mCherry infected hiPSCs at day 3 (SMP_d3) culture with the SMP medium (n = 3 biological replicates per group). (L) Bar graph showing the expression of CALM3 mRNA between the LV‐OE‐ECEL1‐ and the LV‐gcGFP infected hiPSCs at day 3 culture with the vehicle medium (Vehicle_d3) (n = 3 biological replicates per group). (M) Co‐immunoprecipitation of ECEL1 and CALM3 from the total protein lysates of the SMP‐induced, hiPSCs‐differentiated cells, at day 3 followed by immunoblot analysis of the indicated proteins. Input, total lysate before immunoprecipitation; IgG: immunoprecipitation with IgG; IP, immunoprecipitation. (N) Schematic diagram illustrating the experimental procedure for knocking down CALM3 in hiPSCs using CALM3 siRNA and diagram of three small interfering RNA (siRNA) sequences designed for targeting the CALM3 gene. (O) Bar graph showing the expression of CALM3 mRNA in CALM3 siRNA (CALM3 siRNA‐1#, CALM3 siRNA‐2#, and CALM3 siRNA‐3#)‐ and scramble siRNA transfected hiPSCs at day 3 culture with the SMP medium (n = 3 biological replicates per group). (P‐R) Bar graphs showing the mRNA expression of SCN2A (P), SCN3A (Q), and SCN9A (R) between the siRNA (CALM3 siRNA‐2#)‐ and the scramble siRNA transfected hiPSCs‐differentiated cells at day 3 culture with the SMP medium (n = 3 biological replicates per group). (S) Representative traces showing the VGSCs currents of SMP‐induced (3 DIV), hiPSCs‐differentiated cells between the CALM3 siRNA group and the scramble siRNA group. The VGSCs currents were recorded in the voltage‐clamp configuration by delivering voltage steps ranging from −80 to +60 mV for 100 ms, in 10 mV increments. (T) Violin plots showing the averaged current density (pA/pF) of VGSCs of SMP‐induced (3 DIV), hiPSCs‐differentiated cells between the CALM3 siRNA group and the scramble siRNA group (n = 13 cells per group). The VGSCs currents were measured at ‐20 mV holding voltage. (U) Representative traces showing the evoked action potentials of SMP‐induced (3 DIV), hiPSCs‐differentiated cells between the CALM3 siRNA group and the scramble siRNA group. Action potentials were evoked by a series of depolarizing current pulses from 0 pA to +15 pA for 600 ms, in 5 pA increments. (V) Pie chart showing the proportion of action potential firing cells in the SMP‐induced (3 DIV), hiPSCs‐differentiated cells between the CALM3 siRNA group and the scramble siRNA group (n = 13 cells per group). Data are presented as mean ± SEM. ^**^ *p*<0.01; ^****^ *p*< 0.0001. Two‐tailed unpaired *t* test with Welch's correction for (B), (C), (F); Two‐tailed unpaired *t* test for (D), (J‐L), (P‐R), (T); One‐way ANOVA with Dunnett's *post‐hoc* test for (O). See also Figure S8.

Furthermore, we investigated whether overexpression (OE) of ECEL1 in hiPSCs could improve the neuronal functional maturation in the early stage of hiPSCs differentiation. Recombinant lentivirus expressing ECEL1 linked with gcGFP (LV‐OE‐ECEL1) was applied to infect hiPSCs, and overexpress ECEL1 in these cells (Figure S8H,I), and the OE efficiency was validated by the increased expression of ECEL1 mRNA level in the LV‐OE‐ECEL1 group compared to the LV‐gcGFP controls (∼47.7±9.7‐fold, *p* = 0.0407, Figure S8J). Similar results were found in Western blotting analyses, showing that the abundance of ECEL1 protein was increased to ∼11.9±0.4‐fold of the LV‐gcGFP control (*p*<0.0001, Figure S8K,L). Unsurprisingly, overexpression of ECEL1 in hiPSCs, significantly upregulated the expression of *SCN2A* (∼6.3±0.5‐fold, vs. LV‐gcGFP, *p* = 0.0012), *SCN3A* (∼4.6±0.6‐fold, vs. LV‐gcGFP, *p* = 0.0070), and *SCN9A* (∼2.9±0.1, vs. LV‐gcGFP, *p*<0.0001) mRNA levels, in hiPSCs‐differentiated cells at 3 DIV cultured with the vehicle medium (neurobasal/B‐27 media containing 0.1% DMSO) (Figure 7A‐D). Correspondingly, overexpression of ECEL1 in hiPSCs enhanced the inward VGSCs currents in hiPSCs‐differentiated cells at 3 DIV cultured with the vehicle medium (Figure 7E,F). The average current density of VGSCs evoked at ‐20 mV was increased, from ‐0.3±0.3 pA/pF in the LV‐gcGFP group to ‐3.6±1.0 pA/pF in the LV‐OE‐ECEL1 group (*p* = 0.0071, Figure 7F). Moreover, overexpression of ECEL1 in hiPSCs improved the action potential firing of hiPSCs‐differentiated cells, with the proportion of action potential firing cells increased from almost 0% of total recorded cells in the LV‐gcGFP group (n = 17), to ∼15% (3/20) of total recorded cells in the LV‐OE‐ECEL1 group (Figure 7G,H). These data suggest that overexpression of ECEL1 in hiPSCs promotes neuronal functional developmental maturation in the early stage of hiPSCs differentiation, by upregulating the functional assembly and expression of multiple ion channels such as VGSCs in hiPSCs‐derived neurons.

Transcriptomic profiling of the adult human and rodent brain reveals enriched ECEL1 expression in specific regions, including the telencephalon, diencephalon, midbrain, hindbrain, hippocampus, and spinal cord [124, 125, 126, 127]. To determine whether the SMP‐induced, hiPSCs‐differentiated neurons acquire regional identity corresponding to these ECEL1‐enriched domains, we performed RNA‐seq analysis to examine the alterations of these region‐specific gene markers with ECEL1 genes, and their correlation‐ship, in the process of hiPSCs‐differentiation from SMP_d0 to SMP_d15 (Figure S8M,N). Significant upregulation of these region‐specific markers, including telencephalon (*POU3F2*, *CUX1*, *CUX2*), diencephalon​ (*SIX3*, *RAX*), midbrain (*OTX2*, *LMX1B*, *NR4A2*), hindbrain (*EGR2*, *GBX2*), hippocampal (*PROX1*), and spinal cord (*HOXC9*, *NKX6‐1*) markers [128, 129, 130, 131, 132, 133, 134, 135, 136, 137, 138], with increased expression of ECEL1 gene in these regions, was observed at 15 DIV of the SMP‐induced, hiPSCs‐differentiation (Figure S8M). Moreover, correlation analysis validated a positive association between the expression levels of ECEL1 and these region‐specific markers (Figure S8N). These findings raise the possibility that, the regulatory mechanisms of ECEL1‐mediated human neuronal functional developmental maturation, are probably specific to this particular cell population. Of course, whether these ECEL1‐mediated regulatory mechanisms may represent a general human neuronal developmental process, need be further studied.

To further identify which component(s) in the SMP combination is(are) essential factor(s) involved in the upregulation of ECEL1 mRNA level, a strategy of adding one factor per trial to the vehicle medium was performed, to investigate the effect of “vehicle plus one factor” on the differentiation of hiPSCs (Figure S8O). The results from RT‐qPCR showed that compared to the vehicle control, the abundance of *ECEL1* mRNA level increased ∼18.2‐fold after 3 days of “vehicle plus db‐cAMP” culture (*p*<0.0001, vs. vehicle), and ∼6.5‐fold after 3 days of “vehicle plus forskolin” culture (*p* = 0.0016, vs. vehicle) (Figure S8O), indicating that the two cAMP‐signaling activators, including db‐cAMP and forskolin in the combination of SMP, are likely involved in regulating ECEL1 expression and further promoting functional maturation of human neurons.

Next, we investigated whether the two other candidate genes, *MEIS3* and *TNNT1*, which were identified in aforementioned correlation analysis (Figure 6G,H), were also involved in neuronal functional developmental maturation in the early stage of SMP‐induced hiPSCs differentiation. Using small interfering RNAs (siRNAs) targeting MEIS3 (MEIS3 siRNA‐1#, ‐2#, ‐3#) or TNNT1 (TNNT1 siRNA‐1#, ‐2#, ‐3#), to knock down MEIS3 and TNNT1 expression in hiPSCs, respectively (Figure S8P,Q). The knockdown efficiency of MEIS3 siRNAs and TNNT1 siRNAs was validated by RT‐qPCR analysis, and the results revealed that MEIS3 siRNA‐2# and TNNT1 siRNA‐3# exhibited the respective highest efficiency (Figure S8R,S). For MEIS3 siRNA‐2#, the MEIS3 mRNA level was reduced to ∼7.6±0.3% of the control (*p*<0.0001, vs. scrambled siRNA, Figure S8R); for TNNT1 siRNA‐3#, the TNNT1 mRNA level was decreased to ∼11.9±0.6% of the control (*p*<0.0001, vs. scrambled siRNA, Figure S8S), therefore, the MEIS3 siRNA‐#2 and TNNT1 siRNA‐3# were selected for subsequent functional experiments. Notably, knockdown of either MEIS3 or TNNT1 in hiPSCs, showed no significant effects on the inward VGSCs currents of the hiPSCs‐differentiated cells at 3 DIV of SMP induction (Figure S8T,U). The average current density of VGSCs evoked at ‐20 mV was ‐25.8±7.1 pA/pF in the scrambled siRNA group, ‐23.5±4.8 pA/pF in the MEIS3 siRNA group (*p* = 0.9907, vs. scrambled siRNA), and ‐24.6±6.2 pA/pF in the TNNT1 siRNA group (*p* = 0.9980, vs. scrambled siRNA) (Figure S8U). Moreover, knockdown of either MEIS3 or TNNT1 in hiPSCs did not significantly affect the action potential firing capability of the differentiated neurons (Figure S8V,W). The proportion of action potential firing cells was ∼84.62% (11/13) in the scrambled siRNA group (n = 13), compared to ∼76.92% (10/13) in the MEIS3 siRNA group (n = 13), and ∼84.62% (11/13) in the TNNT1 siRNA group (n = 13) (Figure S8W). These results reveal a fundamental distinction between the roles of these regulators: unlike ECEL1, whose knockdown severely disrupts neuronal maturation, knockdown of either MEIS3 or TNNT1 does not impair this process under our experimental conditions. These findings suggest that while MEIS3 and TNNT1 show transcriptional correlation with neuronal maturation, they likely play modulatory or secondary roles, and are probably not primary drivers of neuronal functional development. Their expression patterns may thus represent correlates rather than determinants of neuronal electrophysiological maturation.

Finally, we identified the downstream target molecule mediating the regulatory effects of ECEL1 on the functional assembly and expression of ion channels like VGSCs, which are involved in the neuronal functional developmental maturation in the early stage of SMP‐induced hiPSCs differentiation. Protein‐protein interaction (PPI) network analysis revealed that ECEL1 may regulate calmodulin 3 (CALM3), which in turn forming connections with *SCN2A*, *SCN3A*, and *SCN9A* that encodes Nav1.2, Nav1.3, and Nav1.7, respectively (Figure 7I). *CALM3* encodes calmodulin (CaM), a multifunctional calcium ion sensor that mediates numerous calcium‐dependent neural development processes [139, 140]. Indeed, the expression of *CALM3* mRNA level was significantly increased in hiPSCs‐differentiated cells at 3 DIV of SMP induction (∼3.8±0.03‐fold of control, *p*<0.0001, vs. SMP_d0, Figure 7J). In addition, knockdown (KD) of ECEL1 in hiPSCs by LV‐shECEL1, substantially downregulated the expression of *CALM3* mRNA level in hiPSCs‐differentiated cells at 3 DIV of SMP induction (decreased by ∼22.3±1.5%, *p*<0.0001, vs. LV‐mCherry, Figure 7K), whereas overexpression (OE) of ECEL1 in hiPSCs by LV‐OE‐ECEL1, statistically upregulated the expression of *CALM3* mRNA level in hiPSCs‐differentiated cells at 3 DIV of SMP induction (∼1.8±0.1‐fold, *p* = 0.0017, vs. LV‐gcGFP, Figure 7L). Although knockdown and overexpression of ECEL1 in hiPSCs bidirectionally regulated *CALM3* mRNA expression, co‐immunoprecipitation (Co‐IP) analysis demonstrated the absence of a direct interaction between ECEL1 and CALM3 (Figure 7M), suggesting that CALM3 may be a downstream target of ECELl, which is probably indirectly regulated by ECEL1 through other molecules. To test whether CALM3 is involved in ECEL1‐mediated functional assembly and expression of VGSCs, and in the neuronal functional developmental maturation in the early stage of SMP‐induced hiPSCs differentiation, we examined effects of knocking down *CALM3* in hiPSCs on ECEL1‐mediated upregulation of *SCN2A*, *SCN3A*, and *SCN9A* mRNA levels, as well as on the enhancement of VGSCs currents and action potential firing rate, in hiPSCs‐differentiated cells at 3 DIV of SMP induction. Three small interfering RNA (siRNA) targeting *CALM3*, including CALM3 siRNA‐1#, CALM3 siRNA‐2#, and CALM3 siRNA‐3#, were designed to infect hiPSCs and knock down CALM3 expression in these cells, respectively (Figure 7N). The knockdown efficiency of CALM3 siRNAs was validated by RT‐qPCR analysis, and the results showed that the CALM3 siRNA‐2# displayed the most significant knockdown efficiency, and reduced the CALM3 mRNA level to 25.6±1.1% of the control (*p*<0.0001, vs. scrambled siRNA, Figure 7O). Thus, the CALM3 siRNA‐2# was selected for the followed CALM3 knockdown experiments. As our expectation, knockdown of CALM3 in hiPSCs robustly downregulated the expression of *SCN2A* (decreased to ∼58.2±2.9%, versus scrambled siRNA, *p* = 0.0023), *SCN3A* (decreased to ∼61.5±0.8%, vs. scrambled siRNA, *p* = 0.0022), and *SCN9A* (decreased to ∼56.7±1.6%, vs. scrambled siRNA, *p* = 0.0017) mRNA levels in hiPSCs‐differentiated cells at 3 DIV of SMP induction (Figure 7P‐R). Consistently, knocking down CALM3 in hiPSCs also reduced the inward VGSCs currents in hiPSCs‐differentiated cells at 3 DIV of SMP induction (Figure 7S,T). The average current density of VGSCs evoked at ‐20 mV was decreased, from ‐48.1±8.3 pA/pF in the scrambled siRNA group to ‐16.9±5.3 pA/pF in the CALM3 siRNA group (*p* = 0.0041, Figure 7T). Also, knockdown of CALM3 in hiPSCs robustly inhibited the action potential firing of hiPSCs‐differentiated cells, with the proportion of action potential firing cells decreased, from ∼84.62% (11/13) of total recorded cells in the scrambled siRNA group (n = 13) to ∼69.23% (9/13) of total recorded cells in the CALM3 siRNA group (n = 13) (Figure 7U,V). These results indicate that knockdown of CALM3 in hiPSCs disrupts the neuronal functional developmental maturation in the early stage of SMP‐induced hiPSCs differentiation, by inhibiting the functional assembly and expression of multiple ion channels such as VGSCs in hiPSCs‐derived neurons.

**FIGURE 8:**
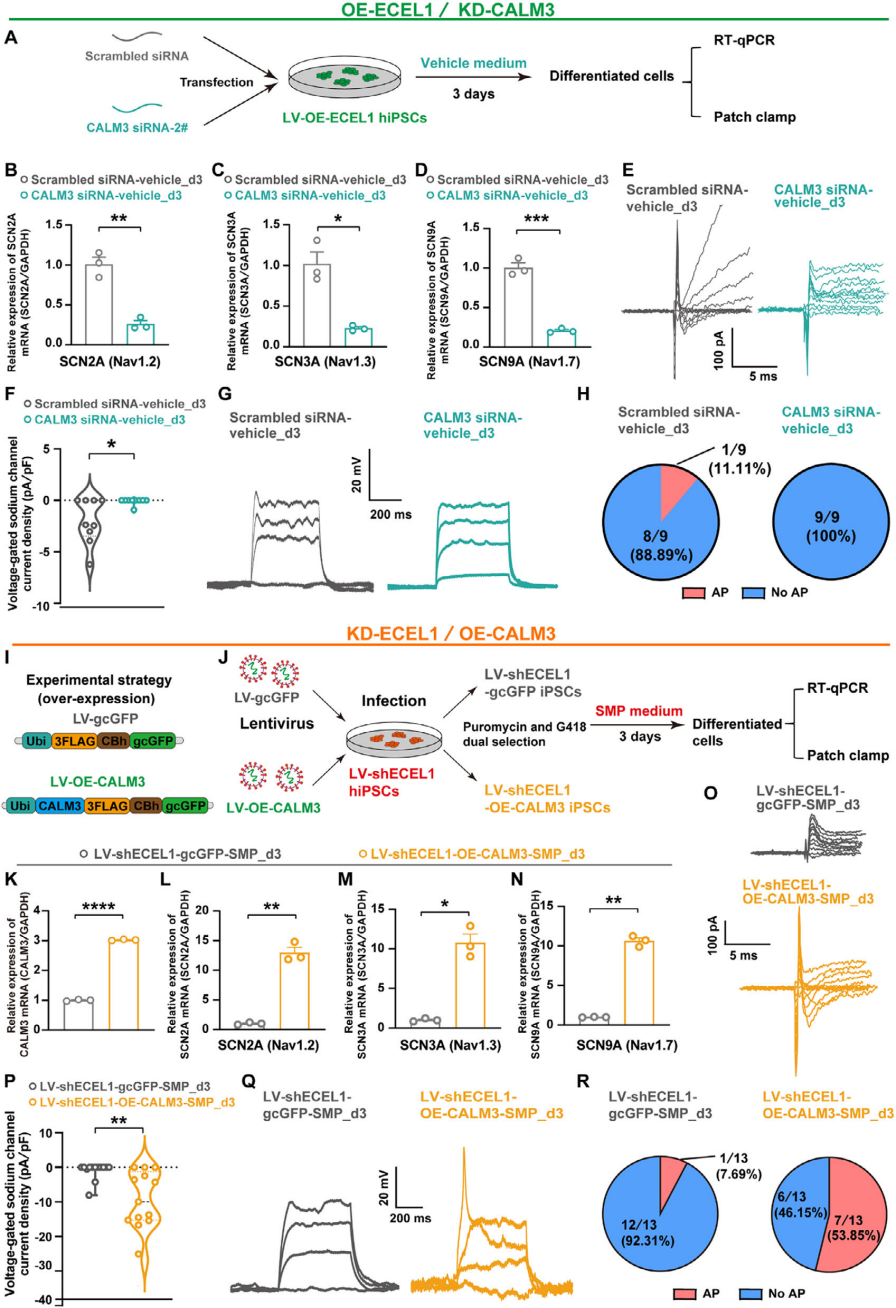
Involvement of ECEL1 and its downstream target molecule CALM3 in the functional developmental maturation of SMP‐induced, hiPSCs‐differentiated neurons. (A) Schematic diagram depicting the experimental procedure of knocking down CALM3 in LV‐OE‐ECEL1 hiPSCs using CALM3 siRNA, for investigating the effects of knocking down CALM3 gene in LV‐OE‐ECEL1 hiPSCs (OE‐ECEL1/KD‐CALM3), on VGSCs genes expression (by RT‐qPCR) and electrophysiological changes (by patch clamp) of hiPSCs‐differentiated neurons, at 3 days in vitro (DIV) culture using the vehicle medium (neurobasal/B‐27 media containing 0.1% DMSO). (B‐D) Bar graphs showing the mRNA expression of SCN2A (B), SCN3A (C), and SCN9A (D) in LV‐OE‐ECEL1‐hiPSCs‐differentiated cells (3 DIV vehicle medium culture) between the CALM3 siRNA group and the scramble siRNA group (n = 3 biological replicates per group). (E) Representative traces showing the VGSCs currents of LV‐OE‐ECEL1‐hiPSCs‐differentiated cells (3 DIV vehicle medium culture) between the CALM3 siRNA group and the scramble siRNA group. The VGSCs currents were recorded in the voltage‐clamp configuration by delivering voltage steps ranging from −80 to +60 mV for 100 ms, in 10 mV increments. (F) Violin plots showing the averaged current density (pA/pF) of VGSCs of LV‐OE‐ECEL1‐hiPSCs‐differentiated cells (3 DIV vehicle medium culture) between the CALM3 siRNA group and the scramble siRNA group (n = 9 cells per group). The VGSCs currents were measured at ‐20 mV holding voltage. (G) Representative traces showing the evoked action potentials of LV‐OE‐ECEL1‐hiPSCs‐differentiated cells (3 DIV vehicle medium culture) between CALM3 siRNA group and the scramble siRNA group. Action potentials were evoked by a series of depolarizing current pulses from 0 to +15 pA for 600 ms, in 5 pA increments. (H) Pie chart showing the proportion of action potential firing cells in the vehicle‐culture (3 DIV), LV‐OE‐ECEL1‐hiPSCs‐differentiated cells between the CALM3 siRNA group and the scramble siRNA group (n = 9 cells per group). (I) Schematic diagram depicting the construction of recombinant lentivirus (LV) expressing CALM3 plasmid linked with gcGFP (LV‐OE‐CALM3). (J) Lentivirus infection of LV‐shECEL1 hiPSCs using LV‐OE‐CALM3, for investigating the effects of overexpressing CALM3 gene in LV‐shECEL1 hiPSCs (KD‐ECEL1/OE‐CALM3), on VGSCs genes expression (by RT‐qPCR) and electrophysiological changes (by patch clamp) of hiPSCs‐differentiated neurons, at 3 days in vitro (DIV) culture using the SMP medium. (K) Bar graph showing the expression of CALM3 mRNA between the LV‐OE‐CALM3‐ and the LV‐gcGFP infected LV‐shECEL1 hiPSCs, at 3 days in vitro (DIV) culture using the SMP medium. (n = 3 biological replicates per group). (L‐N) Bar graphs showing the mRNA expression of SCN2A (L), SCN3A (M), and SCN9A (N) in LV‐shECEL1‐hiPSCs‐differentiated cells (3 DIV SMP medium culture), between the LV‐OE‐CALM3‐ and the LV‐gcGFP infected group (n = 3 biological replicates per group). (O) Representative traces showing the VGSCs currents of LV‐shECEL1‐hiPSCs‐differentiated cells (3 DIV SMP medium culture), between the LV‐OE‐CALM3‐ and the LV‐gcGFP infected group. The VGSCs currents were recorded in the voltage‐clamp configuration, by delivering voltage steps ranging from −80 to +60 mV for 100 ms, in 10 mV increments. (P) Violin plots showing the averaged current density (pA/pF) of VGSCs in LV‐shECEL1‐hiPSCs‐differentiated cells (3 DIV SMP medium culture), between the LV‐OE‐CALM3‐ and the LV‐gcGFP infected group (n = 13 cells per group). The VGSCs currents were measured at −20 mV holding voltage. (Q) Representative traces showing the evoked action potentials of LV‐shECEL1‐hiPSCs‐differentiated cells (3 DIV SMP medium culture), between the LV‐OE‐CALM3‐ and the LV‐gcGFP infected group. Action potentials were evoked by a series of depolarizing current pulses from 0 pA to +15 pA for 600 ms, in 5 pA increments. (R) Pie chart showing the proportion of action potential firing cells in the SMP‐culture (3 DIV), LV‐shECEL1‐hiPSCs‐differentiated cells, between the LV‐OE‐CALM3‐ and the LV‐gcGFP infected group (n = 13 cells per group). Data are presented as mean ± SEM. ^*^ *p*<0.05; ^**^ *p*<0.01; ^***^ *p*<0.001; ^****^ *p*< 0.0001. Two‐tailed unpaired *t* test for (B), (D), (K); Two‐tailed unpaired *t* test with Welch's correction for (C), (F), (L‐N), and (P).

To further determine the functional relationship between CALM3 and ECEL1, we investigated whether knocking down CALM3 (by CALM3 siRNA) in hiPSCs, affected the aforementioned effects of overexpressing ECEL1 on the neuronal functional developmental maturation (Figure 8A). The results showed that knockdown of CALM3 in LV‐OE‐ECEL1‐iPSCs, robustly downregulated the expression of *SCN2A* (decreased to ∼26.0±3.7%, vs. scrambled siRNA, *p* = 0.0017), *SCN3A* (decreased to ∼22.8±1.7%, vs. scrambled siRNA, *p* = 0.0308), and *SCN9A* (decreased to ∼20.7±1.3%, vs. scrambled siRNA, *p* = 0.0002) mRNA levels in LV‐OE‐ECEL1‐iPSCs‐differentiated cells, at 3 DIV cultured with the vehicle medium (Figure 8B–D). Consistently, knocking down CALM3 in LV‐OE‐ECEL1‐iPSCs also reduced the inward VGSCs currents in LV‐OE‐ECEL1‐iPSCs‐differentiated cells, at 3 DIV cultured with the vehicle medium (Figure 8E,F). The average current density of VGSCs evoked at ‐20 mV was decreased, from ‐2.0±0.7 pA/pF in the scrambled siRNA group to ‐0.1±0.1 pA/pF in the CALM3 siRNA group (*p* = 0.0330, Figure 8F). Also, knockdown of CALM3 in LV‐OE‐ECEL1‐iPSCs inhibited the firing of action potential of LV‐OE‐ECEL1‐iPSCs‐differentiated cells, with the proportion of action potential firing cells decreased from ∼11.11% (1/9) of total recorded cells in the scrambled siRNA group (n = 9), to 0% (0/9) of total recorded cells in the CALM3 siRNA group (n = 9) (Figure 8G,H). These results indicate that knockdown of CALM3 in hiPSCs abrogated the overexpressing ECEL1‐induced upregulation of *SCN2A*, *SCN3A*, and *SCN9A* mRNA expression, as well as the enhancement of VGSCs currents and action potential firing rate.

Besides, we investigated whether overexpression (OE) of CALM3 in hiPSCs following ECEL1 knockdown, could rescue the aforementioned effects of knocking down ECEL1 on the neuronal functional developmental maturation in the early stage of SMP‐induced hiPSCs differentiation. Recombinant lentivirus expressing CALM3 linked with gcGFP (LV‐OE‐CALM3) was applied to infect LV‐shECEL1‐hiPSCs (KD‐ECEL1‐hiPSCs) and overexpress CALM3 (OE‐CALM3) in these cells (Figure 8I,J). The OE efficiency was validated by the increased expression of CALM3 mRNA level in the LV‐shECEL1‐OE‐CALM3 (KD‐ECEL1/OE‐CALM3) group compared to the LV‐shECEL1‐gcGFP controls (∼3.0±0.01‐fold, *p*<0.0001, Figure 8K), in hiPSCs‐differentiated cells at 3 DIV of SMP induction. Expectedly, OE CALM3 in KD‐ECEL1‐hiPSCs, significantly upregulated the expression of *SCN2A* (∼12.9±0.8‐fold, vs. LV‐shECEL1‐gcGFP, *p* = 0.0055), *SCN3A* (∼10.7±1.0‐fold, vs. LV‐shECEL1‐gcGFP, *p* = 0.0125), and *SCN9A* (∼10.6±0.3, vs. LV‐shECEL1‐gcGFP, *p*<0.0001) mRNA levels, in hiPSCs‐differentiated cells at 3 DIV of SMP induction (Figure 8L–N). Correspondingly, OE CALM3 in KD‐ECEL1‐hiPSCs enhanced the inward VGSCs currents in hiPSCs‐differentiated cells at 3 DIV of SMP induction (Figure 8O,P). The average current density of VGSCs evoked at ‐20 mV was increased from ‐0.4±0.3 pA/pF in the LV‐shECEL1‐gcGFP group, to −10.2±2.3 pA/pF in the LV‐shECEL1‐OE‐CALM3 group (*p* = 0.0037, Figure 8P). Moreover, OE CALM3 in KD‐ECEL1‐hiPSCs improved the action potential firing of hiPSCs‐differentiated cells, with the proportion of action potential firing cells increased from ∼7.69% of total recorded cells in the LV‐shECEL1‐gcGFP group (n = 13), to ∼53.85% (7/13) of total recorded cells in the LV‐shECEL1‐OE‐CALM3 (KD‐ECEL1/OE‐CALM3) group (Figure 8Q,R). These data suggest that OE CALM3 in KD‐ECEL1‐hiPSCs rescues knocking down ECEL1‐induced impairment of neuronal functional developmental maturation in the early stage of hiPSCs differentiation, by upregulating the functional assembly and expression of multiple ion channels, such as VGSCs in hiPSCs‐derived neurons.

Collectively, these findings suggest that ECEL1 likely underlies human neuronal functional developmental maturation in the early stage of SMP‐induced hiPSCs differentiation, through CALM3‐mediated functional assembly and expression of multiple ion channels (e.g. VGSCs) in hiPSCs‐derived neurons.

## 3 Discussion

In this study, we established an in vitro human neuronal model derived from hiPSCs using a combined small molecules and proteins (SMP) protocol. While several well‑established protocols exist for differentiating hiPSCs into neurons, such as dual SMAD inhibition (SMADi) [34, 35, 36, 141, 142, 143] or forced expression of neurogenic transcription factors [37, 38, 39], these methods usually require a prolonged period, where the cell typically undergo commitment to neural progenitor cells (NPCs) over 2–3 weeks, followed by differentiation into neurons over 4–12 weeks, thereby failing to capture the rapid, early events of human neuronal morphogenesis and functional maturation [17, 40, 41]. These limitations highlight the necessary to develop a new, extended differentiation protocol that can drive hiPSCs toward a functional neuronal fate rapidly and synchronously, particularly for modeling early neural developmental processes. Here, we applied a stepwise design of the SMP strategy, of which candidate small molecules were selected from the literature on the basis of their established roles in neuronal development and maturation, functioning as agonists or inhibitors of key signaling pathways [66, 67, 68, 69, 70, 71, 72, 73, 74, 75], and some soluble proteins were included, given their critical contributions to neuronal differentiation [80, 81, 82, 83, 84, 85, 86]. Of note, these small molecules were chosen to target distinct signaling pathways critical for neural differentiation and maturation (e.g., ROCK inhibition by Y27632 [42, 104], cAMP elevation by forskolin and db‑cAMP [100, 144, 145, 146], Notch suppression by DAPT [147, 148]), while recombinant proteins provided extracellular support and trophic factors (BDNF, GDNF, laminin) [40, 149, 150] which are essential for neuronal survival and synaptogenesis. Importantly, these SMP components were arranged in a defined ratio and temporal schedule to overcome stage‑specific bottlenecks. Unlike existing methods that focus on either progenitor generation or late‑stage maturation [17, 151, 152], this SMP integration strategy enabled simultaneous acceleration of differentiation, enhancement of survival, and promotion of functional maturation, which cannot be achieved by simply combining individual published recipes. In fact, many components of the SMP molecule‐set have already been used in existing protocols for differentiating human iPSCs into neurons; and also, our investigation into the mechanisms of early protrusion formation indeed revealed that key effectors, such as the ROCK inhibitor Y27632 and cAMP elevators (forskolin, db‐cAMP), are already well‐established components of previously published neuronal differentiation protocols [144, 153, 154]. Nevertheless, the SMP protocol's innovation is defined by the strategic integration of known effectors to co‐activate synergistic signaling pathways. This integrated approach enables accelerated differentiation and maturation beyond what is achievable with single‐component or sequential strategies. Hence, the novelty of the SMP protocol lies not in the inclusion of completely new molecules, but in the strategic integration of known effectors into a defined ratio and temporal schedule. This functionally complementary combination enables synergistic acceleration of differentiation, enhancement of survival, and promotion of functional maturation, which cannot be achieved by simply combining individual published recipes, as evidenced by the comparison with the conventional SMAD inhibition protocol [35, 36].

Importantly, this SMP‐induced, hiPSCs‐differentiated neuronal model exhibits fundamental characteristics of neurons, including neuronal morphology, expression of neural‐specific markers, electrophysiological properties, and transcriptional profile features of neuronal lineage genes. Also, this neuronal model can replicate the characteristics of early neuronal cytoskeletal growth and the timeline of neuronal functional maturation, providing a reliable tool for investigating the developmental mechanisms of human neurons in vitro. Notably, the total time frame required for generating functional neurons by using this SMP protocol is approximately one month, which is considerably shorter than those described in conventional differentiation methods, where the cells typically undergo commitment to neural progenitor cells (NPCs) over 2–3 weeks, followed by differentiation into neurons over 4–12 weeks [36, 141, 142, 143, 153, 154, 155, 156, 157, 158]. Our morphological and electrophysiological data further demonstrate that this SMP protocol induced neurons express mature neuronal markers and are capable of firing repeated action potentials at 15–30 DIV, indicating that the SMP protocol enables rapid, synchronized production of functional neurons, thereby offering practical advantages for applications requiring accelerated cell generation, such as high throughput drug screening or disease modeling, where batch consistency and shortened timelines are critical. Although the SMP protocol shortens the overall timeline, it does not simply bypass the key steps of normal neuronal development. Instead, it accelerates the process so that essential features, such as orderly cytoskeleton growth and progressive functional maturation, occur in a condensed period. Indeed, morphological and electrophysiological data demonstrate that neurite outgrowth and neural network formation follow a sequence similar to that seen in previous reported longer protocols [32, 41, 47, 48], and that action potential firing and spontaneous activity emerge in a developmentally ordered manner [41, 49, 50]. These findings indicate that the SMP‐induced hiPSCs differentiation into neurons retains core aspects of human neuronal development, making it suitable for modeling early developmental processes even with a reduced timescale. Nevertheless, because the process is condensed, some aspects of the precise regulatory mechanisms may differ from those in human neural development, and this model should be validated against in vivo developmental profiles​ in future studies.

Cellular skeletal morphology characterized by axons and dendrites is a key feature distinguishing neurons from other cell types. In the in vitro neuronal differentiation protocols, changes in morphology and the expression of cytoskeletal proteins are commonly used as indicators of successful neuronal conversion. In this study, we unexpectedly observed that cells rapidly formed multiple protrusive structures with a certain degree of complexity in the early stage (1–4 h) of SMP‐induced hiPSCs differentiation. Using a strategy of sequential exclusion method, including “SMP minus‐1 factor” and “SMP minus multifactor”, we demonstrate that the ROCK inhibitor Y27632 and other four compounds of SMP, including forskolin, ISX‐9, db‐cAMP, and GABA, which are related to the elevation of intracellular calcium ion (Ca^2+^) concentration, are required for rapid cellular cytoskeleton development in the process of SMP‐induced hiPSCs differentiation. Notably, regarding early protrusion formation, or more precisely the very initial steps of neurite elongation, several components of SMP cocktail do not appear to contribute to this process, while those elements that do play a role are already well‐established components of previously published neuronal differentiation protocols (e.g., ROCK inhibitor, cAMP) [78, 159, 160]. Similar results were obtained analyzing the relationship of ECEL1 and cAMP. These findings raise the possibility that the novelty of the SMP protocol lies in its timing, combination, or context of application rather than its individual composition.

The Rho/ROCK signaling pathway has a negative regulatory role in the initial growth of neuronal dendrites, and inhibition of the ROCK signaling pathway by Y27632 can induce neuronal cells to grow dendrites within one day [42]. In addition, the increase of intracellular Ca^2+^ concentration is also closely related to the growth of neuronal dendrites [103, 161]. However, the use of conventional reagents such as ionomycin or thapsigargin, to induce a rapid increase in intracellular Ca^2+^ concentration, was not able to elicit cellular protrusions growth and neuron conversion of hiPSCs. It is reported that ISX 9 can activate neurogenic lineage‐related genes in cells by mediating calcium ion influx through voltage‐gated calcium channels (e.g., Ca_v_1.2 and Cav1.3) and NMDA receptors (NMDARs) [102]. Using D‐AP5 (an NMDARs antagonist) or Cd^2+^ (cadmium ions, a broad‐spectrum VGCCs inhibitor) to inhibit the activity of NMDARs and VGCCs, respectively, we identified that it is VGCCs rather than NMDARs, play a key role in rapid neuronal morphological development in the process of SMP‐induced hiPSCs differentiation. Furthermore, using multiple‐technique approaches, including various specific channel blockers, RNA‐seq, and gene editing techniques, we demonstrate that both of Cav1.2 (encoded by *CACNA1C*) and Cav1.3 (encoded by *CACNA1D*), the two subtypes of L‐type voltage‐gated calcium channels, play crucial roles in early neuronal morphogenesis in the process of SMP‐induced hiPSCs differentiation. Consistently, both of Cav1.2 and Cav1.3 are reported playing key roles in neuronal differentiation and maturation, thus inhibiting these channels by the channels’ blocker nimodipine abrogates the Ca_v_1.2/1.3 channels action for neuronal differentiation [43, 162]. L‐type calcium channels are also involved in the regulation of multiple neurotrophic factors, neurotransmitters, and cell adhesion molecules, by which to affect the growth cone structure, induce the release of actin from the membrane skeleton of growth cones, and promote neuronal protrusions growth [163]. In particular, Ca_v_1.2 has been reported to be closely related to neuronal maturation in the process of in vitro neuronal differentiation in other types of stem cells, such as dental pulp stem cells [164]. Also, studies using CACNA1C and CACNA1D knockout mice models have reported reductions in dendritic complexity and the number and length of mossy fiber bouton (MFB) filopodia [110, 165, 166], indicating a role of Cav1.2 and Cav1.3 channels in neurite development. Consistently, we observed that knockout of CACNA1C or CACNA1D significantly impairs the initial stages of neuronal polarization and process outgrowth, verifying their conserved function in cytoskeletal remodeling while highlighting their specific impact in a human developmental context. Our findings in this human iPSC‐derived neuronal model not only extend these observations to human neurons but also refine the understanding of their roles in early morphogenesis. Moreover, there is existing evidence linking these channels to neuronal fate commitment [109, 167], as well as associations with psychiatric disorders [168]. Although our present study focuses on cellular morphogenesis, the established association of *CACNA1C* and *CACNA1D* genetic variations with neurodevelopmental disorders [168, 169, 170] underscores the broader significance of these channels, in particular their crucial roles in human neuronal development, which can be effectively investigated using our human iPSC‐derived neuronal model. Of note, despite NMDARs also mediating calcium influx of neurons and contributing to the increase of intracellular calcium levels, however, we rule out the involvement of NMDARs in early neuronal morphogenesis in the process of SMP‐induced hiPSCs differentiation by the use of NMDARs antagonist D‐AP5. We cannot explain this puzzlement, and further studies are needed. Collectively, our current findings suggest that both Cav1.2 and Cav1.3, the two subtypes of L‐type voltage‐gated calcium channels, are essential for early morphogenesis of human neuronal development.

In view of the rapid functional maturation of hiPSCs‐differentiated neurons, we also explored the potential mechanisms underlying human neuronal functional developmental maturation by using the SMP‐induced, hiPSCs‐differentiated neuronal model. We first identified 14 differentially expressed genes (DEGs) that served as potential candidate regulators for human neuronal functional developmental maturation, by RNA‐seq analysis from the SMP_d3 group and the SMP_d0 group. Then, using a Patch‐seq technique to determine the key genes related to human neuronal functional developmental maturation [32, 171], we revealed that among the 14 DEGs, *ECEL1* exhibited the strongest correlation with neuronal functional maturation, indicating that ECEL1 may serve as a key regulator of human neuronal functional developmental maturation in the early stage of SMP‐induced hiPSCs differentiation. *ECEL1*, a gene encoding endothelin‐converting enzyme‐like 1 (ECEL1) protein, is also called the damage‐induced neuronal endopeptidase (DINE) gene in the rodent genome [115, 116]. ECEL1 is a kind of membrane‐bound metalloproteinase (a member of the neprilysin family) [118], and has been identified as the gene responsible for distal arthrogryposis syndromes [115]. As a neuronal peptide endopeptidase, ECEL1 plays a pivotal role in nervous system development [117, 118], and promotes neuronal and axonal regeneration in response to neuronal damage or nerve injury [119, 120]. In the early stages of nervous system development, ECEL1 is crucial for the proper distribution of motor nerve terminals [117]. It has been reported that the expression of ECEL1 is significantly upregulated in motor neurons following spinal cord injury [116], and the elevated ECEL1 promotes axonal regeneration of damaged neurons [119]. Moreover, using LV‐shRNA targeting ECEL1, we found that knockdown of *ECEL1* in the hiPSCs significantly reduces the mRNA expression levels of voltage‐gated sodium ion channels (VGSCs), including Nav1.2, Nav1.3, and Nav1.7, in hiPSCs‐differentiated cells at 3 DIV SMP induction. Consistently, knockdown of *ECEL1* in the hiPSCs substantially decreases the inward VGSCs currents, and inhibits the action potentials firing in t SMP‐induced (3 DIV), hiPSCs‐differentiated cells. By contrast, overexpression of ECEL1 in the hiPSCs not only upregulates the mRNA levels of Nav1.2, Nav1.3, and Nav1.7, but also enhances the inward VGSCs currents and increases the action potential firing in the 3 DIV of the SMP‐induced (3 DIV), hiPSCs‐differentiated cells. These findings indicate that ECEL1 likely mediates human neuronal functional developmental maturation through promoting rapid functional assembly and expression of VGSCs such as Nav1.2, Nav1.3, and Nav1.7, in hiPSCs‐derived neurons in the early stage of SMP‐induced hiPSCs differentiation. Studies have revealed that ECEL1 expression in the developing mouse brain is predominantly characteristic of the telencephalon, diencephalon, midbrain, hindbrain, hippocampus, and spinal cord [124, 125, 126, 127]. In line with these findings, our RNA‐seq data also revealed a significant upregulation of these region‐specific markers, including telencephalon (POU3F2, CUX1, CUX2), diencephalon (SIX3, RAX), midbrain (OTX2, LMX1B, NR4A2), hindbrain (EGR2, GBX2), hippocampal (PROX1), and spinal cord (HOXC9, NKX6‐1) markers, with increased expression of ECEL1 in these regions, at 15 DIV of the SMP‐induced, hiPSCs‐differentiation. Consistently, correlation analysis validated a positive association between the expression levels of ECEL1 and these region‐specific markers. These findings raise the possibility that, the regulatory mechanisms of ECEL1‐mediated human neuronal functional developmental maturation are probably specific to this particular cell population. Of course, whether these ECEL1‐mediated regulatory mechanisms may represent a general human neuronal developmental process need be further studied.

Based on the protein‐protein interaction (PPI) network analysis, we also identified a downstream target molecule of ECEL1, named CALM3, which encodes calmodulin, a multifunctional calcium ion sensor that mediates numerous calcium‐dependent neural development processes [139, 140]. Moreover, we found an increased expression of *CALM3* mRNA level in SMP‐induced (3 DIV), hiPSCs‐differentiated cells; and also, the *CALM3* mRNA level can be bidirectionally modulated by knocking down or overexpressing ECEL1 in SMP‐induced, hiPSCs‐differentiated cells. Besides, knockdown of *CALM3* in hiPSCs abrogates the ECEL1‐mediated upregulation of *SCN2A*, *SCN3A*, and *SCN9A* mRNA levels, as well as the enhancement of VGSCs currents and action potential firing rate, in SMP‐induced (3 DIV), hiPSCs‐differentiated cells, whereas overexpressing *CALM3* following ECELl knockdown, rescues the impaired effects of knocking down ECELl on neuronal functional developmental maturation in the early stage of hiPSCs differentiation, by upregulating the functional assembly and expression of multiple ion channels such as VGSCs in hiPSCs‐derived neurons. These findings strengthen the mechanistic link between ECELl, CALM3, and VGSCs regulation. Of note, although *CALM3* mRNA expression can be regulated bidirectionally by ECEL1 knockdown or overexpression, the absence of a direct interaction between ECEL1 and CALM3 (demonstrated by Co‐IP analysis) suggests that CALM3 may be a downstream target of ECELl, which is probably indirectly regulated by ECEL1 through other molecules. Altogether, these data provide several lines of evidence demonstrating that, ECEL1 likely underlies human neuronal functional developmental maturation in the early stage of SMP‐induced hiPSCs differentiation, through CALM3‐mediated functional assembly and expression of multiple ion channels (e.g. VGSCs) in hiPSCs‐derived neurons.

It is noteworthy that while excitatory synaptic function is well established in this hiPSCs‐differentiated neuronal model, inhibitory synaptic activity is limited, as evidenced by the absence of detectable inhibitory postsynaptic currents (IPSCs), which may be attributed to the extremely low proportion of GABAergic inhibitory interneurons (approximately 2.0%) within the differentiated cell populations, thereby limiting the formation of functional inhibitory synapses in our current model. Nevertheless, the apparent spontaneous firing activity of the differentiated cell populations, associated with rhythmic neural oscillatory activity in theta, alpha, beta, and gamma frequency bands, suggests the formation of functional neural networks in the late stage (60 DIV) of the SMP‐induced hiPSCs differentiation.

**FIGURE 9:**
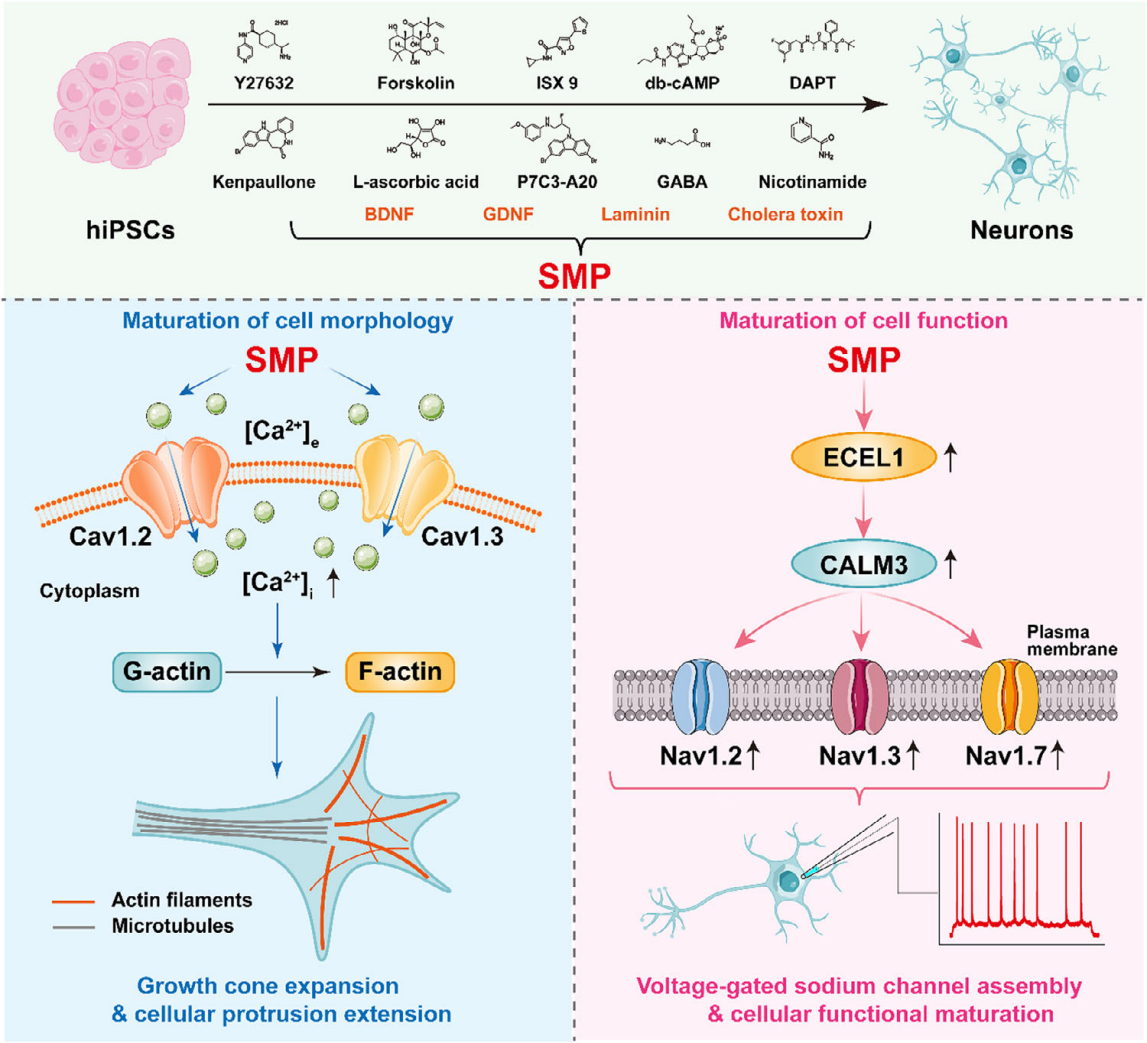
Schematic summary illustrating the morphological and functional maturation mechanisms underlying human neural development using the SMP‐induced, iPSCs‐differentiated neurons. This study developed an in vitro human neuronal model derived from hiPSCs with rapid morphological and functional maturity, by using combined small molecules and proteins (SMP) protocol. Both of Cav1.2 and Cav1.3, the two subtypes of L‐type voltage‐gated calcium channels, and their mediated calcium ion influx, are essential for early morphogenesis of human neuronal development, which coordinates actin and microtubule dynamics to promote growth cone expansion and protrusion extension. Moreover, this study identified a novel gene, ECEL1 (endothelin converting enzyme‐like 1), which underlies human neuronal functional developmental maturation in the early stage of SMP‐induced hiPSCs differentiation, through calmodulin 3 (CALM3)‐mediated functional assembly and expression of multiple ion channels (e.g. voltage‐gated sodium ion channels) in neuronal functional development maturation.

In conclusion, our data suggest that the SMP‐induced, hiPSCs‐differentiated neurons can be used as an in vitro human neuronal model for the study of human neural development, as inferred by neuronal cytoskeleton development, electrophysiological functional maturation, and neural network formation. Moreover, we discovered that both of Cav1.2 and Cav1.3, the two subtypes of L‐type voltage‐gated calcium channels, are essential for early morphogenesis of human neuronal development. In addition, we identified a novel gene *ECEL1*, which underlies human neuronal functional developmental maturation in the early stage of SMP‐induced hiPSCs differentiation, through CALM3‐mediated functional assembly and expression of multiple ion channels (e.g. VGSCs) in hiPSCs‐derived neurons (Figure 9). Our findings uncover novel mechanisms underlying the morphogenesis and functional maturation of human neurons that are involved in human brain development. This hiPSCs‐differentiated human neuronal model successfully captures key neural developmental processes, including the formation of axon initial segments, the emergence of network‐level rhythmic oscillations, and the maturation of excitatory synaptic function, although it currently has limitations in inhibitory circuitry representation due to the predominantly glutamatergic neuronal population.

## 4 Experimental Section

### 4.1 Animals

Immunocompromised male BALB/c nude mice aged 3 weeks were provided by the Department of Experimental Animal Sciences, Peking University Health Science Center. All mice were housed in separate cages, and the room was kept at 22 ± 2°C and 50 to 60% humidity under a 12‐h light/12‐h dark cycle with ad libitum access to food and water. All animal experimental procedures were approved by the Animal Care and Use Committee of Peking University (LA2021177).

### 4.2 Urinary‐Derived Epithelial Cells Collection and Expansion

The urine samples were non‐invasively collected from three healthy volunteers, including a 27‐year‐old male, a 30‐year‐old male, and a 27‐year‐old female. All of them provided informed written consent, with approval granted by the Biomedical Ethics Committee of Peking University (IRB00001052‐22142). Urinary‐derived epithelial cells were isolated and cultured from the urine as described previously with minor modifications. [59, 172] Briefly, approximately 50 mL of urine was collected into sterile 50 mL tubes and centrifuged at 400g for 10 min at room temperature. The supernatant was gently removed, leaving approximately 1 mL of liquid in each tube. Then, 24 mL of phosphate‐buffered solution (PBS) containing penicillin (100 U/mL) and streptomycin (100 µg/ml) antibiotic was added and centrifuged again at 200 g for 10 min at room temperature. The supernatant was carefully removed, leaving behind only 0.5 mL of liquid in each tube. Then, 1 mL of the urinary cell culture medium (UCM) 1 was added to re‐suspend the cell pellet, and then transferred the volume into a 1% gelatin‐coated dish. The cells were incubated at 37°C for 6 days. The UCM1 medium contained Dulbecco modified Eagle medium (DMEM)/F12 (1×), fetal bovine serum (FBS) (5%), primocin antibiotic solution (0.2%), holo‐transferrin (10 µg/mL), sodium selenite (10 ng/mL), insulin (10 µg/mL), hydrocortisone (0.5 µg/mL), L‐epinephrine (0.5 µg/mL), L‐thyroxin (6.5 ng/mL), and recombinant human epidermal‐growth‐factor (rhEGF) (50 ng/mL). On the 6th day after plating, small colonies (approximately twenty cells) could be observed under an inverted microscope. Next, 1.5 mL of UCM1 medium was removed and replaced with 1.5 mL of urinary cell culture medium (UCM) 2, and the cells were incubated at 37°C for an additional 6 days. Apart from FBS (0.5%), other compositions in the UCM2 medium are same as in the UCM1 medium. From the 12th day after plating, half of the UCM2 medium was changed daily until the cell density reached 80%. Finally, the obtained urinary cells were separated at a ratio of 1:3 into new dishes to expand quickly. All chemicals and reagents used in the present study are listed in Table S1.

### 4.3 Human Pluripotent Stem Cells Derivation and Maintenance

The hiPSCs were acquired by reprogramming the urinary‐derived epithelial cells as described in the previous reports. [60, 61, 173, 174] The urinary‐derived epithelial cells were digested by TrypLE Express Enzyme, and harvested by centrifuged (200 g, 3 min), resuspended in 100 µL of Opti‐MEM medium with episomal plasmid mixtures (pCXLE‐hOCT3/4‐shp53‐F, pCXLE‐hSK, pCXLE‐hUL, and pCXWB‐EBNA1, 2 µg each), transfected by using the electroporator (NEPA21) according to the manufacturer's instructions. Subsequently, resuspended mixtures with 2 mL of the UCM2 medium were transferred to Matrigel coated dishes. At 48 h post‐transduction, fresh UCM2 medium containing 100 ng/mL of rhFGF‐2 was added. On day 4, half of the medium was replaced with reprogramming medium, and on day 6, the medium was completely replaced with reprogramming medium. The reprogramming medium contained DMEM/F12 medium (1×), N2 supplement (100×), B27 supplement (50×), L‐glutamine (1 mM), non‐essential amino acid (NEAA, 1%), 2‐mercaptoethanol (0.1 mM), sodium pyruvate (1 mM), recombinant human fibroblast growth factor (rhFGF)‐2 (100 ng/mL), L‐ascorbic acid (300 µM), PD0325901 (0.5 µM), CHIR99021 (3 µM), A‐83‐01 (0.5 µM), HA‐100 (10 µM), and recombinant human leukemia inhibitory factor (rhLIF) (10 ng/mL). Half of the media was changed every day until 14 days after transfection, and on day 15, media were switched into mTeSR1 medium until hiPSC colonies appeared. From day 25 to day 30 of transfection, hiPSC colonies were picked and transferred to Matrigel‐coated dishes for expansion. Human iPSCs were passaged routinely with ethylenediaminetetraacetic acid (EDTA) as described previously. [175] Briefly, passages every 5–6 days using PBS‐EDTA medium (0.5 mM EDTA in PBS, 340 mOsm), splitting in the ratio of 1:5. In this study, three iPSC lines derived from three healthy volunteers’ urinary‐derived epithelial cells were named iPSC‐1#, iPSCs‐2# and iPSCs‐3#, respectively. Human embryonic stem cell lines H1 (RRID: CVCL_9771) and H9 (RRID: CVCL_9773) were originally obtained from the WiCell Research Institute (Madison, WI) with no contamination. All cells were cultured under 5% CO_2_ at 37°C with no contamination.

### 4.4 Generation of Induced Neurons from hiPSCs

For SMP‐induced neural differentiation, hiPSCs were disassociated with TrypLE Express enzyme, resuspended in the SMP1 medium, containing neurobasal medium (1×), B27 supplement (50×), Y27632 (10 µM), forskolin (10 µM), ISX 9 (20 µM), db‐cAMP (500 µM), DAPT (10 µM), kenpaullone (5 µM), L‐ascorbic acid (200 µM), P7C3‐A20 (3 µM), GABA (300 µM), nicotinamide (1 mM), BDNF (5 ng/mL), GDNF (20 ng/mL), laminin (1.25 µg/mL), and cholera toxin (10 ng/mL), and plated at the density of 100 000 cells/cm^2^ on Matrigel‐coated plates for 3 days. Three days later, the medium was changed to the SMP2 medium, including neurobasal medium (1×), B27 supplement (50×), db‐cAMP (500 µM), DAPT (10 µM), L‐ascorbic acid (200 µM), GABA (300 µM), BDNF (5 ng/mL), GDNF (20 ng/mL), and laminin (1.25 µg/mL) for another 2 days. The culture medium was changed by alternating between the SMP1 medium (for 3 days) and the SMP2 medium (for 3 days) until day 15. At day 15, medium was changed to maturation medium, containing neurobasal medium (1×), B27 supplement (50×), DAPT (10 µM), L‐ascorbic acid (200 µM), BDNF (5 ng/mL), GDNF (20 ng/mL), and laminin (1.25 µg/mL). Half of the maturation medium was refreshed every 3 days.

For neural differentiation using the dual SMAD inhibition (SMADi) protocol [34, 35, 36], hiPSCs were cultured in the neural induction medium, containing DMEM/F12 medium (2×), neurobasal medium (2×), N2 supplement (100×), B27 supplement (50×), L‐glutamine (1 mM), LDN193189 (100 ng/mL), and SB431542 (10 µM), and plated at the density of 100 000 cells/cm^2^ on Matrigel‐coated plates. The culture medium was changed every day from day 0 to day 12. After the appearance of neural rosettes, the neural progenitor cells (NPCs) were enzymatically dissociated using Accutase, and then passaged onto laminin‐coated plates. The culture medium was subsequently replaced with neural maintenance medium, containing DMEM/F12 medium (2×), neurobasal medium (2×), N2 supplement (100×), B27 supplement (50×), L‐glutamine (1 mM), and rhFGF‐2 (20 ng/mL). The culture medium was changed every other day from day 12 to day 17. For neuron maturation, NPCs were dissociated with Accutase and cultured in the neural maturation medium, containing neurobasal medium (2×), B27 supplement (50×), L‐glutamine (1 mM), BDNF (10 ng/mL), GDNF (10 ng/mL), and plated at a density of 100 000 cells/cm^2^ on laminin‐coated plates. Half of the maturation medium was refreshed every 2 days.

### 4.5 Alkaline Phosphatase Staining

For alkaline phosphatase staining, cells were fixed with PBS containing 4% paraformaldehyde (PFA) for 5 min at room temperature, following the established protocol from the manufacturer (Beyotime Biotechnology) for the BCIP/NBT Alkaline Phosphatase Color Development Kit. Alkaline phosphatase‐stained (purple) pluripotent colonies were observed by a light microscope.

### 4.6 Immunofluorescence Staining

To prepare cell samples for immunofluorescence analysis, the cells were gently washed twice with PBS, fixed with 4% paraformaldehyde (PFA) for 10 min, and permeabilized with PBST (phosphate‐buffered saline with 0.1% Triton X‐100) for another 10 min. After blocking with 1% bovine serum albumin (BSA)/PBS for 1 h at room temperature, cells were incubated with primary antibodies for overnight at 4°C. Then, after three washes in PBS, the cells were incubated with secondary antibodies for 1 h at room temperature. In some experiments, the cells were counterstained with the nuclear marker DAPI (4',6‐Diamidino‐2‐phenylindole, 1 µM)/ Hoechst 33342 (20 µM) carrying blue fluorescence for 10 min at room temperature. After three washes in PBS, the cells were covered with glycerol and stored at 4°C until visualization. Visualization of fluorescence signal was performed by confocal microscopy at excitation wavelengths of 405 nm (blue), 488 nm (green), 561 nm (red), and 647 nm, respectively. At least three fields per section were analyzed to establish reproducibility. Detailed information on the antibodies used in this study are shown in Table S1.

### 4.7 RNA Extraction, Reverse Transcription‐PCR and Real‐Time Quantitative PCR

Total RNA was extracted from the cells with TRIzol reagent (Life Technologies). One microgram of total RNA was used for reverse transcription reaction with PrimeScript 1st Strand cDNA Synthesis Kit (Takara), according to the manufacturer's instructions. Polymerase Chain Reaction (PCR) was performed with TaKaRa Taq Kit (Takara). The reactions were set up on the basis of the manufacturer's protocol. Real‐time quantitative PCR (RT‐qPCR) was performed with GoTaq qPCR Master Mix (Promega) and analyzed with the 7500 real‐time PCR system (Applied Biosystems). Briefly, a 20‐µL PCR reaction that included 1 µL of complementary DNA, 10 µL of GoTaq qPCR Master Mix, and 0.2 mM of each primer was used and adjusted to the final volume with nuclease‐free water. *GAPDH* in parallel for each run was used as an internal control. Quantitative PCR conditions were incubation at 95°C for 2 min followed by 40 cycles of thermal cycling (15 s at 95°C, and 60 s at 60°C). The relative expression ratio of mRNA was quantified via the 2^−ΔΔCT^ method. Primer sequences are shown in Table S2.

### 4.8 Karyotype Analysis

The karyotype (chromosomal G‐band) analyses were carried out at Beijing KINGMED Clinical Laboratory, using standard protocols for high‐resolution G‐banding (400G–550G) and analyzed by an automatic karyotyping analysis system (Leica GSL120). For each analysis, at least 20 metaphases were examined.

### 4.9 Bisulfite Sequencing

Genomic DNA was extracted from cells using the TIANamp Genomic DNA Kit (TIANGEN) following the manufacturer's instructions. One milligram of genomic DNA was treated with DNA Bisulfite Conversion Kit (TIANGEN), according to the manufacturer's recommendations. The promoter regions of the human *OCT3/4* and *NANOG* genes were amplified by PCR using LA Taq Hot Start Version (Takara). The PCR products were subcoloniesed into pMD18‐T Vector. Ten clones of each sample were verified by sequencing. Primer sequences [21] used for PCR amplification are shown in Table S2.

### 4.10 In Vitro hiPSCs Differentiation and Embryoid Body (EB) Formation

For embryoid body (EB) formation [21], hiPSCs were harvested by treating with TrypLE Express enzyme. The clumps of the cells were transferred to ultra‐low attachment 96‐well plates (Corning) in EB medium containing DMEM/F12 medium (1×), KnockOut serum replacement (20%), NEAA (1%), and 2‐mercaptoethanol (0.1mM). The medium was changed every other day. After 8 days as a floating culture, EBs were transferred to 0.1% gelatin‐coated plate and cultured in the same medium for another 8 days.

### 4.11 In Vivo Teratoma Formation

To examine the developmental potentiality of hiPSCs in vivo, the hiPSCs (2.0×10^6^ cells) were harvested by TrypLE Express enzyme treatment, and injected into the hind limb muscles of 3‐week‐old immunocompromised nude mice (approximately 2.0×10^6^ cells confluency per mouse) provided by the Department of Experimental Animal Sciences, Peking University Health Science Center. After 8 weeks, teratomas were dissected and fixed in 4% paraformaldehyde. Samples were embedded in paraffin and processed with hematoxylin and eosin staining to examine the differentiation capacity of the transplanted hiPSCs.

### 4.12 Electrophysiology

Whole‐cell patch‐clamp recordings on cultured cells were performed at room temperature using an EPC‐10 amplifier and Patch‐Master software (HEKA, Freiburg, Germany), which was performed according to previously described methods with some modifications. [176, 177, 178] The recording pipette had a resistance of 4 to 8 megohms (MΩ) when filled with an internal solution containing K‐gluconate (114 mM), HEPES (10 mM), KCl (6 mM), EGTA (5 mM), Na_2_GTP (0.3 mM), and MgATP (4 mM), pH was adjusted to 7.3 with KOH. The external solution contained NaCl (140 mM), KCl (2.5 mM), CaCl_2_·2H_2_O (1.3 mM), MgSO_4_·7H_2_O (1 mM), NaH_2_PO4 (1.2 mM), HEPES (10 mM), and D‐glucose (10 mM), pH was adjusted to 7.4 with NaOH. Membrane currents and action potentials were measured with both pipette and membrane capacitance cancellation, filtered at 2 kHz, and digitized at 10 kHz. Resting membrane potential (RMP) was measured immediately after rupture of the cell membrane in whole‐cell patch mode.

Under voltage‐clamp recording, cells were clamped at −70 mV, and series resistance was compensated to 75∼90%. Na^+^ currents and K^+^ currents were recorded in the voltage‐clamp configuration by delivering voltage steps ranging from ‐80 mV to +60 mV for 100 ms, in 10 mV increments. For the recording of spontaneous excitatory postsynaptic currents (sEPSCs), the cells were held at ‐70 mV with the addition of bicuculline (20 µM) in the external solution for blocking inhibitory postsynaptic currents. Spontaneous inhibitory postsynaptic currents (sIPSCs) were recorded using a Cs^+^‐based internal solution​ containing Cs‐methanesulfonate (130 mM), HEPES (10 mM), Na‐methanesulfonate (8.3 mM), NaCl (1.7 mM), CaCl_2_·2H_2_O (1 mM), EGTA (10 mM), MgATP (2 mM), Na_2_GTP (0.3 mM), pH was adjusted to 7.4 with CsOH. Meanwhile, the cells were held at 0 mV with the addition of 6‐cyano‐7‐nitroquinoxaline‐2,3‐dione (CNQX; 10 µM) and DL‐2‐amino‐5‐phosphonopentanoic acid (D‐APV; 50 µM) in the external solution for blocking excitatory postsynaptic currents [179, 180].

Under current‐clamp recording, the cells were held at 0 pA to detect spontaneous action potentials. To further examine the firing properties of neurons, a series of depolarizing current pulses from ‐10 pA to +20 pA for 600∼2000 ms, in 2∼5 pA increments, were injected to elicit action potentials. Parameters of the action potential, such as frequency, threshold, peak, amplitude, duration, maximum rise slope, half‐width duration, inter‐spike intervals (ISI), and amplitude of after‐hyperpolarization (AHP) were then recorded and analyzed. The properties of action potentials were analyzed as described in previous reports. [32, 89] Origin software 2024 (OriginLab) was used for the electrophysiological data analysis.

### 4.13 RNA Sequencing

RNA sequencing was performed using the DNBSEQ platform provided by BGI Corporation to examine the global gene expression of the SMP_d0 group cells (hiPSCs), the SMP_d3 group cells (day 3 after the SMP‐induced, hiPSCs differentiated cells), and the SMP_d15 group cells (day 15 after the SMP‐induced, hiPSCs differentiated cells). Each group comprised three independent samples, resulting in a total of nine samples. Reference genome version was GCF_000001405.39_GRCh38.p13. The standardized gene expression measurement known as TPM (transcript per kilobase per million mapped reads) was used to identify differentially expressed genes (DEGs). DEGs with expression level changes≥2‐fold were applied for Gene Ontology (GO) analysis. Data analysis was performed using the BGI software analysis platform and R language software (RStudio).

### 4.14 Dynamic Monitoring of Live Cell Imaging

For dynamic monitoring of live cell imaging, hiPSCs were dissociated by using TrypLE Express enzyme, resuspended in appropriate medium media according to experimental conditions and plated on the dishes, followed by a 30‐min incubation for cell adhesion. Live cell imaging was performed using a live cell workstation, that is a fully automated microscopic imaging system (YEESPEC). Images of live cells were captured every 2 min over a 5‐h period under 5% CO_2_ at 37°C. The identification of process‐bearing cells was based on the presence of cellular protrusions visible in images captured with the 10× objective lens.

### 4.15 Scanning Electron Microscopy (SEM) Analysis

For scanning electron microscopy (SEM) analysis, [181] the cells were fixed with 2.5% glutaraldehyde solution in PBS for 30 min at room temperature, and then the samples were washed three times with PBS, dehydrated using an alcohol gradient (30%, 50%, 70%, 80%, 90%, 95%, 98%, and 100%), and were lyophilized at −60°C for 12 h. Finally, the samples were sprayed with gold (Au) at a current of 20 µA and observed under the scanning electron microscope (JSM‐7900F).

### 4.16 Transmission Electron Microscopy (TEM) Analysis

For transmission electron microscopy (TEM) analysis, [114] the cells were fixed in 2.5% glutaraldehyde overnight at 4°C. The samples were sent to the Analysis Center of Peking University Health Science Center for further processing. Ultrathin sections (∼50 nm) were cut on a ultramicrotome (Leica, EM UC6), and picked up onto copper grids stained with lead citrate and examined using a transmission electron microscope (JEM1400PLUS).

### 4.17 Determination of the F‐/G‐Actin Ratio

The changes in the F‐/G‐actin ratio were examined using fluorescence staining and Western blotting methods as previously described. [96, 182] The hiPSCs were dissociated using TrypLE Express enzyme, resuspended in the SMP1 medium for the experimental group and the vehicle medium (Neurobasal/B‐27 media containing 0.1% DMSO) was used for the control group.

For fluorescence staining, F‐actin was labeled with Phalloidin‐iFluor 647 Reagent (1:1000 dilution; ab176759, Abcam), and G‐actin was labeled with Alexa Fluor 488 deoxyribonuclease I (300 nM) for 1 h at room temperature. Images were taken using a fluorescence confocal microscope under identical imaging conditions, and the fluorescence intensity was analyzed with ImageJ software for statistical analysis.

For Western blotting, radioimmunoprecipitation assay (RIPA) lysis buffer was employed to lyse cells from the vehicle and experimental groups on ice for 10 min, followed by centrifugation at 100 000×g for 1 h at 4°C. Soluble actin (G‐actin) was collected in the supernatant. The insoluble F‐actin in the pellet was resuspended in lysis buffer with an equal volume of buffer 2 solution containing guanidine hydrochloride (1.5 mM), sodium acetate (1 mM), CaCl_2_·2H_2_O (1 mM), MgATP (1 mM), and Tris‐HCl (20 mM), pH 7.5, and incubated on ice for 1 h, with gentle shaking every 15 min, to convert F‐actin into soluble G‐actin. The samples were then centrifuged at 15 000×g for 30 min, and F‐actin was collected in the supernatant. Samples from the supernatant (G‐actin) and pellet (F‐actin) fractions were analyzed by Western blotting using an antibody specific for actin (1:5000 dilution, ab179467, Abcam).

### 4.18 Western Blotting

For Western blotting analysis [183], cultured cells were immediately homogenized in ice‐cold lysis buffer containing RIPA lysis buffer, and 2% protease and phosphatase inhibitor cocktail. After being rotated at 4°C for 1 h, the homogenates were centrifuged at 12 000 rpm for 10 min to yield the total protein extract in the supernatant, and the supernatant was analyzed. Then, the samples containing 30 µg were denatured and separated through SDS‐PAGE using 6%–12% separating gels and transferred to a PVDF membrane (0.45 µm, Millipore). The membranes were blocked with 5% nonfat milk in Tris‐buffered saline with Tween‐20 [TBST, containing tris‐HCl (20 mM), NaCl (150 mM), and Tween 20 (0.05%), pH 7.5] for 60 min at room temperature, and then incubated with the primary antibody at 4°C overnight. The blots were washed in TBST and then incubated in horseradish peroxidase (HRP)‐conjugated secondary antibody (1:5000 dilution). The protein band was visualized using ECL (enhanced chemiluminescence) reagents (Tanon) and quantified using imaging analysis software (ImageJ). Detailed information on the antibodies used in this study is shown in Table S1.

### 4.19 Calcium Imaging Analysis

Calcium imaging was performed as previously described. [184] For calcium fluorescence imaging of cells using Fura 2‐acetoxymethyl (AM) ester (Fura‐2 AM), the cell culture medium was removed, and the cells were incubated with Fura‐2 AM (2 µM) in vehicle medium (Neurobasal/B‐27 media containing 0.1% DMSO) for 30 min under 5% CO_2_ at 37°C. After incubation, the cells were washed three times with the vehicle medium to remove residual Fura‐2 AM, and then incubated for another 30 min to allow complete de‐esterification of intracellular AM esters. Changes in Fura‐2 fluorescence intensity are presented as the ratio of F340/F380 after background subtraction. The normalized F340/F380 ratio was calculated as either F_before_/F_baseline_ or F_after_/F_baseline_, in which F_baseline_ indicates the mean basal fluorescence intensity of cells before experimental treatment with ionomycin (1 µM), thapsigargin (2 µM), or the SMP1 medium, F_before_ represents the fluorescence intensity of cells before experimental treatment, and F_after_ indicates the fluorescence of cells after experimental treatment. The images were analyzed using commercial software (MetaFluor v7).

For calcium fluorescence imaging of cells using Fluo‐4 AM, the cell culture medium was removed and the cells were incubated with Fluo‐4 AM (2 µM) in BrainPhys neuronal medium (STEMCELL) for 30 min under 5% CO_2_ at 37°C. After incubation, the cells were washed three times with BrainPhys neuronal medium to remove residual Fluo‐4 AM, and then incubated for a further 30 min. Calcium imaging (×40 magnification) were acquired at 3 Hz using a spinning disk microscope (Nikon) with a 488 nm (FITC) filter. Changes in Fluo‐4 fluorescence intensity are depicted as ΔF/F0 (%), that is, the change in fluorescence intensity (ΔF) relative to the initial fluorescence intensity (F0), where ΔF = F‐F0, F represents the fluorescence intensity of cells. The images were analyzed using commercial software (NIS‐Elements).

### 4.20 Multi‐Electrode Array (MEA) Recordings

For multi‐electrode array (MEA) recordings [34, 185, 186, 187], cells were dissociated and plated in a dense monolayer (∼10 000 cells/µL) on MEA chips (60MEA200/30iR‐ITO‐gr, Multichannel Systems, Germany). There are 59 TiN electrodes in an 8×8 grid with 200 µm distance between the electrodes. Multi‐unit neuronal activity and broadband local field potential (LFP) signals were recorded simultaneously using an MEA60 preamplifier (Multichannel Systems). The outputs of the preamplifier were filtered (0.5 and 5 kHz) and sent to NeuroExplorer 5 software for spike‐sorting and signal processing. Spikes were collected at a sampling frequency of 50 kHz, and band‐pass filtered (0.3–3 kHz). LFPs were collected at a sampling frequency of 10 kHz, amplified (300×), and band‐pass filtered (0.5–200 Hz). LFP signals were filtered into five frequency bands: broadband (4–100 Hz), theta (4–8 Hz), alpha (9–12 Hz), beta (13–30 Hz), and gamma (31–100 Hz) for subsequent analysis.

### 4.21 Coomassie Brilliant Blue Staining and Neuronal Morphology Analysis

For Coomassie Brilliant Blue staining, the cells were fixed with 2.5% glutaraldehyde for 30 min and washed three times with distilled water. The cells were then stained with Coomassie Brilliant Blue staining solution, containing Coomassie Brilliant Blue R‐250 (0.1%), methanol (46.5%), acetic acid (7%), and water (46.5%), for 1 h. After three washes in distilled water, the cells were covered with glycerol and stored at 4°C until visualization by inverted microscope (Olympus). For neuronal morphology analysis [11, 32], the ImageJ software were applied on background subtraction to reconstruct cell morphology. Sholl analysis was used for assessing the morphologic structure of cellular protrusions, in which the radius interval of each section was set to 2.5 µm, starting from 10 µm and ending at farthest protrusion ends.

### 4.22 Cell Counting

Human iPSCs were dissociated into single cells and plated at an identical density of 100 000 cells/cm^2^ on Matrigel‐coated plates for differentiation experiments. After 4 h, the medium was gently aspirated, and cells were gently washed once with PBS, fixed with 4% PFA for 10 min, and permeabilized with PBST for another 10 min. Subsequently, cells were stained with the nuclear dye DAPI (1 µM) for 10 min at room temperature. After three washes with PBS, samples were mounted with glycerol. Visualization of fluorescence signal was performed by confocal microscopy at excitation wavelengths of 405 nm (blue). At least three random fields per sample were imaged for quantitative analysis. The number of DAPI‐positive nuclei in each field was counted. The relative cell number for each experimental condition was calculated as: (cell count of the experimental group / cell count of the control group (SMP‐treated group)) × 100%. Data are presented as mean ± SEM from three independent experiments.

### 4.23 Lactate Dehydrogenase (LDH) Assay

Lactate dehydrogenase (LDH) activity was determined by a LDH Release Assay Kit (Beyotime, C0017). The assay was performed according to the manufacturer's instructions. Briefly, LDH converts pyruvate into lactate that reduces the developer to a colored product with absorbance at 490 nm as measured by a microplate reader. LDH release (% of total release) = ((absorbance of experiment well)‐(absorbance of blank well))/((absorbance of cell maximum enzyme activity well)‐(absorbance of blank well)) × 100%.

### 4.24 MTT Assay

Cell cytotoxicity was assessed using a MTT Cell Proliferation and Cytotoxicity Assay Kit (Beyotime, C0009M). The assay was performed according to the manufacturer's instructions. In brief, metabolically active cells reduce the yellow tetrazolium salt MTT to purple formazan crystals that are solubilized in solvent and quantified by measuring the absorbance at 570 nm using a microplate reader. Cell viability (%) was calculated as ((absorbance of experiment well)‐(absorbance of blank well))/((absorbance of control group)‐(absorbance of blank well)) ×100%.

### 4.25 TUNEL Staining Assay

TUNEL staining was performed using a One Step TUNEL Apoptosis Assay Kit (Beyotime, C1088). For measurement of apoptosis, samples were prepared according to the manufacturer's manual. Briefly, cells were washed with PBS and fixed with 4% paraformaldehyde for 30 min, and then were treated with 0.1% Triton X‐100 for 5 min, followed by incubation with TdT enzyme solution for 1 h at 37°C. TUNEL‐positive cells were imaged with a confocal microscopy. The cells were counterstained with Hoechst 33342 dye (20 µM) to visualize cell nuclei, and the cells with green fluorescence (488 nm) were considered as apoptotic cells. Relative apoptosis rate (%) was calculated as (TUNEL‐positive rate of experiment group)/(TUNEL‐positive rate of control group (SMP‐treated group)) ×100%.

### 4.26 Lentivirus Production and Infection

Construction and production of recombinant lentivirus expressing small hairpin RNA (shRNA) targeting *CACNA1C*, *CACNA1D*, or *ECEL1*, as well as those overexpressing *ECEL1* or *CALM3*, were completed by Shanghai Genechem Co., Ltd. Validation of the transfection efficiency of lentivirus infection was performed on hiPSCs. The hiPSCs were exposed to lentivirus at a multiplicity of infection (MOI) of approximately 50 for 16 h. After 16 h, the medium was removed and replaced with fresh culture medium to continue the culture.

### 4.27 Fluorescence‐Activated Cell Sorting (FACS)

The lentivirus‐infected iPSCs were dissociated by TrypLE Express enzyme and filtered through 40 mm cell strainers for ensuring single‐cell suspensions. Fluorescence‐activated cell sorting (FACS) was performed using the FACS Aria II Workstation (BD Biosciences). Fluorescent (mCherry or gcGFP) single cells were sorted and seeded in 96‐well plates.

### 4.28 Generation of CACNA1C‐ and CACNA1D‐KO hiPSCs

Guide RNAs (gRNA) were designed and cloned into YKO‐RP003 plasmid (Guangzhou Ubigene Co., Ltd), targeting exon 2 of human *CACNA1C* or *CACNA1D* gene. Five micrograms (5 µg) of CRISPR‐Cas9‐gRNA expressing respective plasmids were applied to transfect into hiPSCs (1×10^6^ cells), by using Lipofectamine 3000 reagent (Invitrogen) following the manufacturer's instructions. Next day, single cells expressing EGFP were sorted using FACS and seeded into a well of a 96‐well plate. Single hiPSC clone was picked and split into 2 batches (one for genotyping and the other for maintenance). Targeted *CACNA1C* or *CACNA1D* locus were amplified using Taq DNA Polymerase (Takara). PCR products were analyzed by Sanger sequencing. Sequence of gRNAs and primer sequences used for PCR amplification are presented in Table S3.

### 4.29 Patch‐Seq Analysis

Patch‐seq was performed as previously described. [32, 171, 188, 189] Briefly, before single‐cell patch‐clamp electrophysiological recordings, all equipment and items should be cleaned with DNA‐off and RNaseZap to eliminate DNA and ribonuclease (RNase) contamination. The intracellular solution used for Patch‐seq contained K‐gluconate (114 mM), HEPES (10 mM), KCl (6 mM), EGTA (5 mM), Na_2_GTP (0.3 mM), MgATP (4 mM), glycogen (20 µg/mL), and recombinant RNase inhibitor (0.2 U/µL), pH was adjusted to 7.3 with KOH. At the end of the electrophysiological recording, the cells were held in whole‐cell patch‐clamp mode, and a moderate negative pressure was applied for 2 to 3 min. After the cytosol was obtained, the intrapipette solution was pressure‐ejected into 100 µl of lysis buffer (provided by BGI Corporation). Single‐cell sequencing was performed by commercial company (BGI Corporation) using Smart‐seq2 technology for reverse transcription of the single‐cell transcriptome to generate full‐length cDNA libraries. Single‐cell amplified products were assessed using the Qsep400 capillary electrophoresis bio‐fragment analyzer. Cells with cDNA concentration lower than 200 pg/µL or distinct peaks smaller than 600 bp were discarded. The final sequencing libraries were analyzed on a DNBSEQ platform (BGI Corporation). The reference genome version was GCF_000001405.39_GRCh38.p13. Data analysis was performed using BGI software analysis platform and R language software (RStudio).

### 4.30 Protein–Protein Interaction (PPI) Network Analysis

The PPI network was performed using STRING software (version 12.0). The list of differentially expressed genes (DEGs) in RNA‐seq data were analyzed by STRING to predict potential interactions between the corresponding proteins. STRING provided data on both known and predicted protein‐protein associations. The resulting network was analyzed to identify and visualize the interactions among the proteins encoded by the differentially expressed genes.

### 4.31 Small Interfering RNA (siRNA) Preparation and Screening

Small interfering RNAs (siRNAs) targeting *MEIS3*, *TNNT1*, and *CALM3* gene were applied to knock down each respective gene separately in hiPSCs. Briefly, siRNAs were designed and synthesized by Xianghongbio Co., Ltd. The hiPSCs were transfected with siRNA (50 nM) using INTERFERin Reagent (Polyplus), according to the manufacturer's instructions. After 72 h, total RNA was extracted from the cells and RT‐qPCR was performed to study gene expression in these cells. Sequences of siRNAs are listed in Table S3.

### 4.32 Co‐Immunoprecipitation (Co‐IP) Analysis

Co‐immunoprecipitation was performed using the Pierce Co‐Immunoprecipitation Kit according to manufacturer's instructions (Thermo Fisher Scientific) [190, 191]. Briefly, cell pellets were lysed in the IP Lyses/Wash Buffer included in the kit, supplemented with 2% protease and phosphatase inhibitor cocktail. The whole cell extract was separated by centrifugation (13 000 × g, 10 min, 4°C) to obtain the soluble fraction. First, 10 µg of anti‑CALM3 antibody was immobilized onto the coupling resin provided in the kit according to the manufacturer's protocol. Then, 2 mg of the cleared lysate was incubated with the antibody‐coupled resin at 4°C overnight. After incubation, the resin was washed extensively with the provided IP Lysis/Wash Buffer to remove non‑specifically bound proteins. The bound proteins were then eluted using the kit's elution buffer, mixed with 5× SDS‑PAGE loading buffer, and boiled at 95 °C for 5 min. The eluates, along with corresponding input controls​ (5% of the lysate used for IP), were subjected to Western blot analysis for the detection of CALM3 and ECEL1.

### 4.33 Quantification and Statistical Analysis

Statistical analyses were performed with GraphPad Prism (version 9.0), Origin (version 2024) and R language software (RStudio version 4.3.3). All quantitative biochemical data and staining are representative of at least three independent biological replicates. Two‐tailed unpaired *t* test or two‐tailed unpaired *t* test with Welch's correction was used for the comparison of the mean values between two groups. One‐way analysis of variance (ANOVA) with Tukey's or Dunnett's *post‐hoc* test as indicated in the text, or two‐way ANOVA (treatment and time factors) with Sidak's *post‐hoc* test was used for multiple comparison. Quantitative data are presented as means ± SEM (standard error of mean) of at least three independent experiments, and differences with *p*<0.05 were considered statistically significant. ^*^ *p*<0.05; ^**^ *p*<0.01; ^***^ *p*< 0.001; ^****^ *p*< 0.0001; ns., not significant. All statistical data are presented in Table S4, and all source data are presented in Table S5.

## Author Contributions

Y. T. and G.‐G. X. conceived and designed the project. Y. T. performed most experiments with the help of Y.‐C. O., Z.‐X. Z. and S‐Q. C. J.C. assisted with data interpretation. G.G.X. and Y.T. drafted the manuscript. All authors discussed the results and approved the manuscript.

## Conflicts of Interest

The authors declare no conflicts of interest.

## Supporting information




**Supporting File 1**: advs73749‐sup‐0001‐SuppMat.docx.

**Supplemental File 2**: advs73749‐sup‐0002‐VideoS1‐1.mp4.

**Supplemental File 3**: advs73749‐sup‐0004‐VideoS1‐2.mp4.

**Supplemental File 4**: advs73749‐sup‐0005‐VideoS2‐1.mp4.

**Supplemental File 5**: advs73749‐sup‐0006‐VideoS2‐2.mp4.

**Supplemental File 6**: aadvs73749‐sup‐0006‐SuppTableS5.rar.

## Data Availability

The data that support the findings of this study are available from the corresponding author upon reasonable request.
