# Supplementary material for: Unraveling the Morphological and Functional Maturation Mechanisms Underlying Human Neural Development Using iPSCs‐Derived Neuronal Model
Source: Adv Sci (Weinh). 2026 Jan 11;13(18):e12891. doi: 10.1002/advs.202512891 (PMC13042389; doi:10.1002/advs.202512891)
Supplement: Supplementary file 1 — Supporting File 1: advs73749‐sup‐0001‐SuppMat.docx. [file ADVS-13-e12891-s004.docx]

**Supporting Information**

**Unraveling the Morphological and Functional Maturation Mechanisms Underlying Human Neural Development Using iPSCs-derived Neuronal Model**

Yue Tian, Yi-Chun Ou, Zi-Xian Zhang, Jie Cai, Si-Qing Cai, and Guo-Gang Xing

Corresponding author: Guo-Gang Xing, ggxing@bjmu.edu.cn

**Supplemental Figures and Figure Legends**

**Table S1**: Key Resource Table.

**Table S2**: PCR primer sequences.

**Table S3**: Short hairpin RNA (shRNA), guide RNA (gRNA) and small interfering RNA (siRNA) sequences.

**Table S4**: Statistical analyses, related to Figures 1-8, and Figures S1-S8.

**Table S5**: Source data, related to Figures 1-8, Figures S1-S8, and Table S4.

**Video S1-1**: Title and Legend, related to Figure 2.

**Video S1-2**: Title and Legend, related to Figure 2.

**Video S2-1**: Title and Legend, related to Figure S4.

**Video S2-2**: Title and Legend, related to Figure S4.

**Supplemental Figures and Figure Legends**


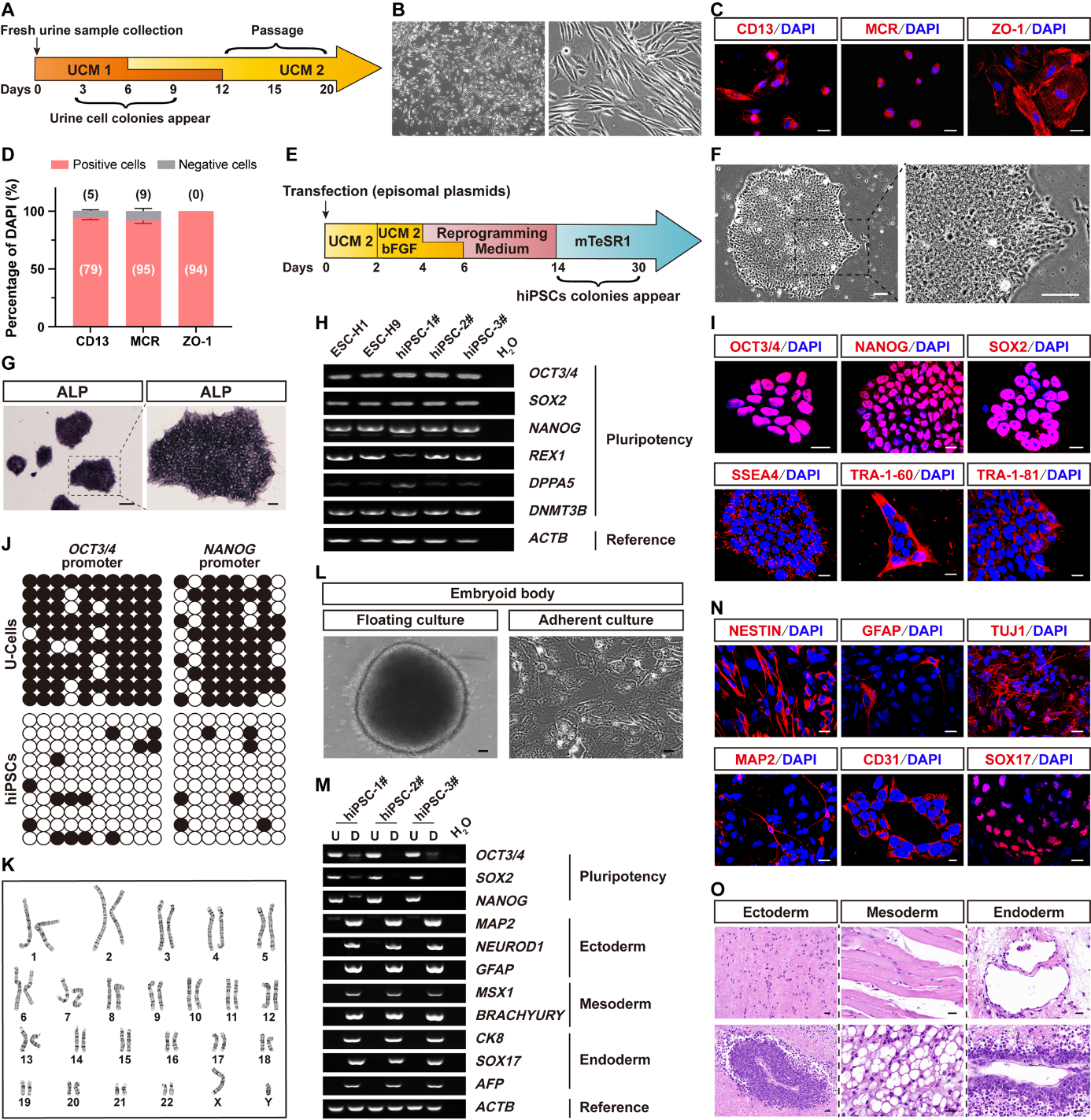


**Figure S1. Generation of human induced pluripotent stem cells (hiPSCs) by reprogramming** **urine-derived epithelial cells under feeder-free conditions. Related to Figure 1.**

(A) Schematic diagram illustrating the experimental procedure for the isolation and expansion of urine-derived epithelial cells. UCM1, urinary cell culture medium 1; UCM2, urinary cell culture medium 2.

(B) Representative phase-contrast images showing the cultured urine-derived epithelial cells at passage one. Scale bar=100 μm. Right: the higher magnification of urine-derived epithelial cells, Scale bar=100 μm.

(C) Representative images showing the immunofluorescence staining with CD13, MCR, and ZO-1, respectively, in cultured urine-derived epithelial cells at passage one. Cell nuclei were counterstained with DAPI (blue). CD13, MCR (mineralocorticoid receptor): renal tubular epithelial cell markers; ZO-1 (Zonula occludes-protein 1): a tight junction marker. Scale bar=20 μm.

(D) Bar graph shows percentage of CD13^+^, MCR^+^, or ZO-1^+^ cells to DAPI^+^ cells. Data are presented as mean ± SEM.

(E) Schematic diagram shows the experimental procedure for generating urine-derived hiPSCs.

(F) Representative phase-contrast images showing a typical urine-derived hiPSCs clone. Scale bar=100 μm. Right: the higher magnification of hiPSCs clone within the white dashed box in (left) are shown. Scale bar=100 μm.

(G) Representative images showing the alkaline phosphatase (ALP) staining with hiPSCs. Scale bar= 200 μm (left), and 50 μm (right), respectively.

(H) RT-PCR analysis of hiPSC-marker genes, including OCT3/4, SOX2, NANOG, REX1, DPPA5, and DNMT3B, in two embryonic stem cells lines (H1, H9), and three urine-derived hiPSCs lines (hiPSC-1#, hiPSC-2#, hiPSC-3#). ACTB was used as a loading control.

(I) Representative images showing the immunofluorescence staining with OCT3/4, NANOG, SOX2, SSEA-4, TRA-1-60 and TRA-1-81 in urine-derived hiPSCs. Cell nuclei were counterstained with DAPI (blue). Scale bar=20 μm.

(J) Bisulfite genomic sequencing of the promoter regions of OCT-3/4 (left) and NANOG (right) in urine-derived epithelial cells (U-Cells) and hiPSCs (hiPSCs-2#) obtained from the same donor. Open circles indicate unmethylated, and filled circles indicate methylated CpG dinucleotides (n=10 per group).

(K) Representative images showing the G-banding chromosome analysis of hiPSCs (hiPSCs-2#) (n=3 biological replicates per group).

(L) Representative images showing a floating cultured embryoid body (EB) (left, 8 days in vitro culture), and the EB-mediated differentiation of hiPSCs (right, the adherent monolayer differentiated cells, 16 days in vitro culture) (n=3 biological replicates per group). Scale bar= 50 μm (left), and 50 μm (right), respectively.

(M) RT-PCR analysis of various differentiation markers of the three germ layers (ectoderm, mesoderm, endoderm) across three hiPSCs lines (hiPSCs-1#, hiPSCs-2#, hiPSCs-3#). ACTB is used as a reference gene. U, undifferentiated cells; D, differentiated cells;

(N) Representative images showing the immunofluorescence staining with various differentiated cell markers, including NESTIN, GFAP, TUJ1, and MAP2 (ectodermal cell markers), CD31 (mesodermal cell marker), and SOX17 (endoderm cell marker), in EB-differentiated hiPSCs (n=3 biological replicates per group). Cell nuclei were counterstained with DAPI (blue). Scale bar=20 μm.

(O) Representative images show the hematoxylin-eosin (HE) staining for hiPSCs-derived teratomas. Left: ectoderm. Up, neural tissue; bottom, neural epithelium. Middle: mesoderm. Up, muscle; bottom, adipose tissue. Right: endoderm. Up, epithelium tissue; bottom, epithelium tissue. Scale bar=20 μm.


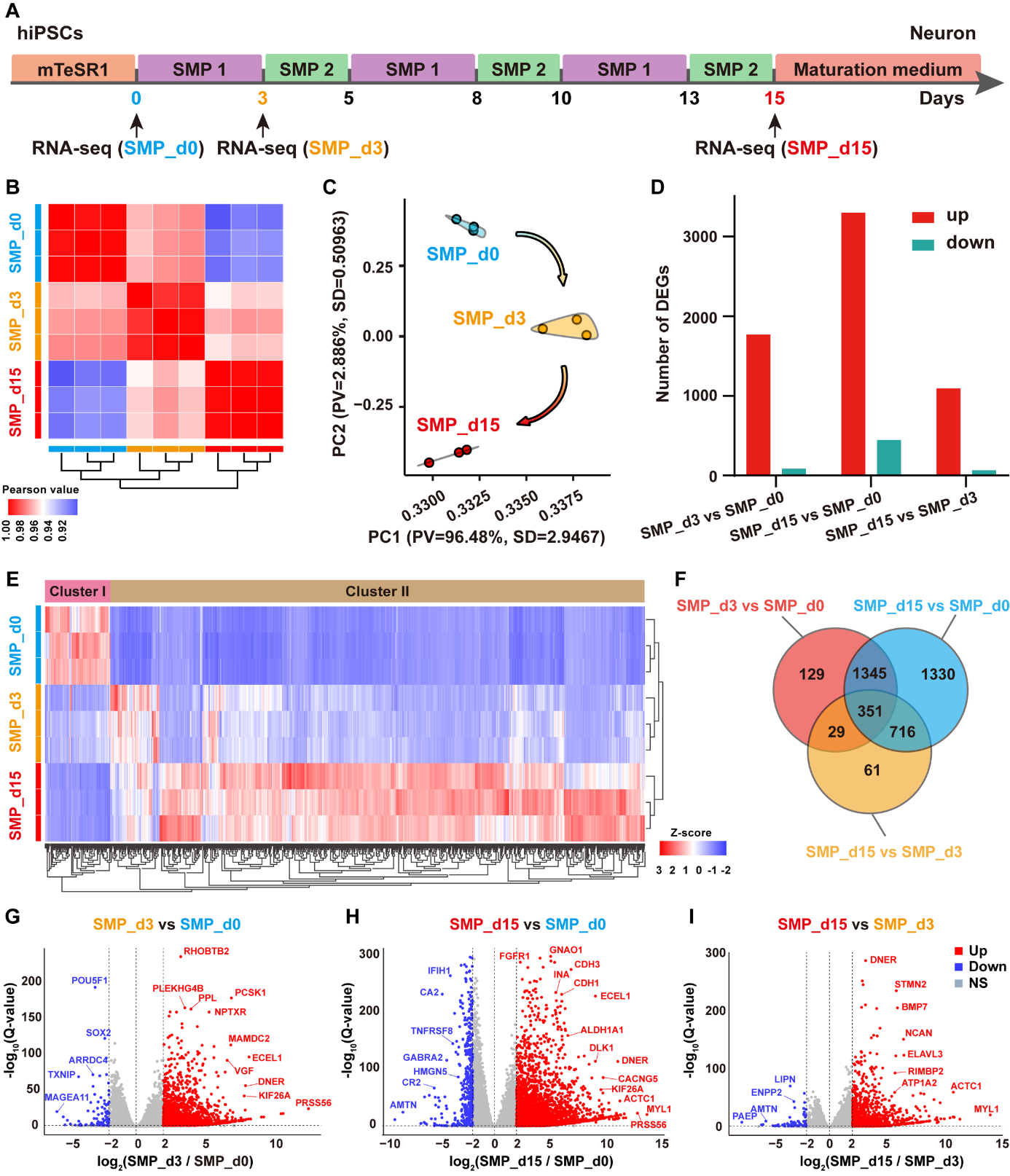


**Figure S2. Changes in the transcriptome profiles of differentiated cells in the process of SMP-induced hiPSCs differentiation (from day 0 to day 3, and to day 15 in vitro culture). Related to Figure 1.**

(A) Schematic diagram showing the experimental procedure for transcriptome sequencing sampling (n=3 biological replicates per group). SMP_d0, day 0 in vitro culture (DIV) of hiPSCs using SMP medium (hiPSCs); SMP_d3, 3 DIV of hiPSC-differentiated cells; SMP_d5, 5 DIV of hiPSC-differentiated cell.

(B) Heatmap showing the Pearson's correlation analysis for the transcriptional profiles of hiPSC-differentiated cells across the SMP_d0, SMP_d3, and SMP_d15 groups (n=3 biological replicates per group).

(C) Principal component analysis (PCA) of RNA-seq datasets showing the transcriptomic differences across the three groups, illustrating a pathway of hiPSCs differentiation from the SMP_d0 group to the SMP_d3 group, and then to the SMP_d15 group (n=3 biological replicates per group). PV, proportion of variance. SD, standard deviation.

(D) Comparison of the differentially expressed genes (DEGs) in the hiPSC-differentiated cells across the three groups (SMP_d0, SMP_d3, and SMP_d15) (n=3 biological replicates per group). Up, up-regulated genes (red); down, down-regulated genes (blue-green). |log2(fold change)| ≥ 2 was considered as the screening criteria to select DEGs.

(E) Heatmap showing the cluster analysis of DEGs in the hiPSC-differentiated cells across the three groups (SMP_d0, SMP_d3, and SMP_d15) (n=3 biological replicates per group). The z-score (row direction) normalization method is adopted and arranged according to the clustering order.

(F) Venn diagram showing the overlapped DEGs among pairwise comparisons of the three groups (SMP_d0, SMP_d3, and SMP_d15) (n=3 biological replicates per group).

(G-I) Volcano plot showing the DEGs compared between the SMP_d3 and the SMP_d0 (G), the SMP_d15 and the SMP_d0 (H), and the SMP_d15 and the SMP_d3 (I) groups, respectively (n=3 biological replicates per group). Up-regulated genes, red dots; down-regulated genes, blue dots; no significant changed genes, gray dots. Vertical dotted lines (black) represent log_2_(fold change) = ±2, and the horizontal dotted line (black) represents Q=0.05.


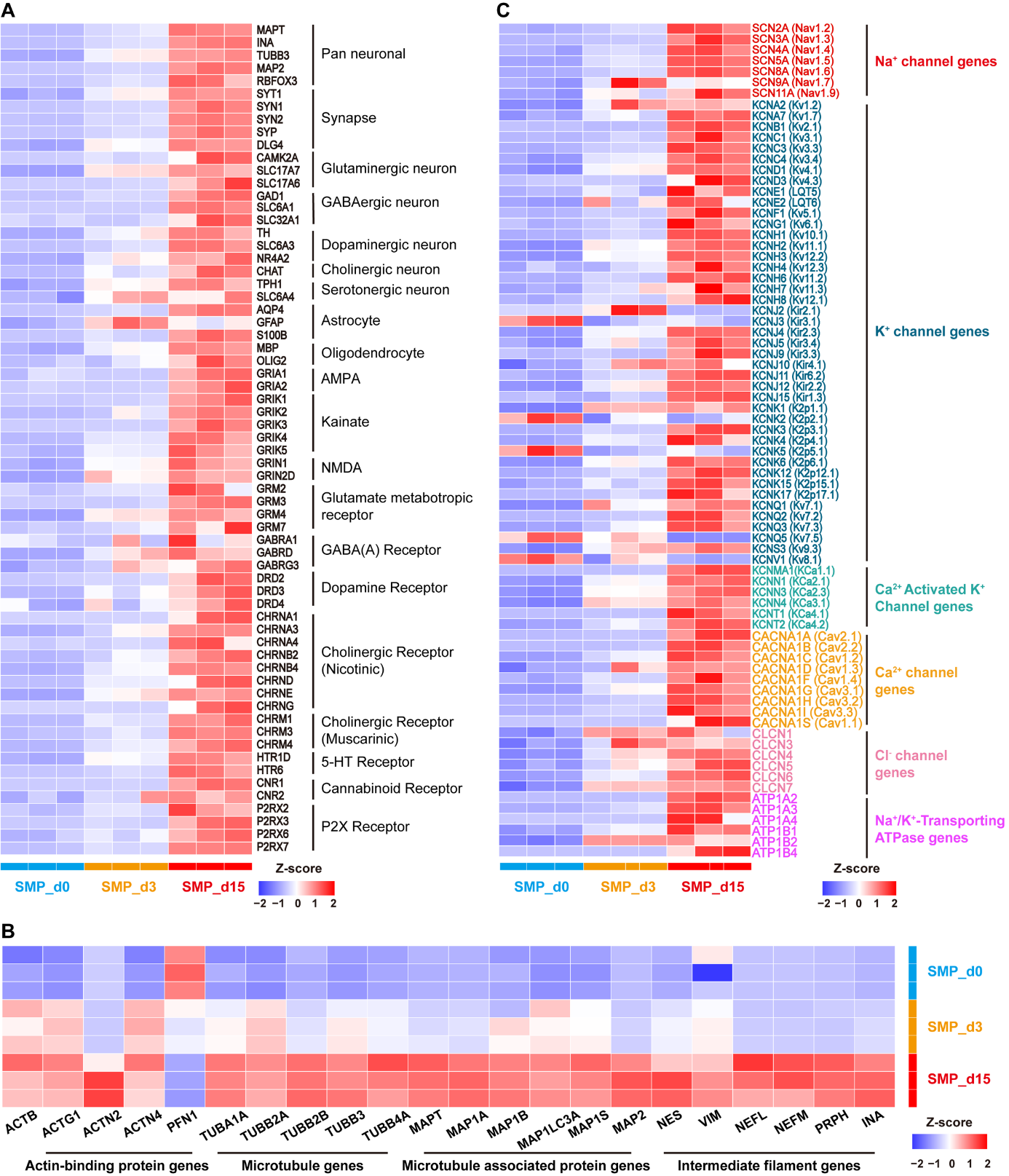


**Figure S3. Multiple genes associated with neuronal differentiation and maturation are involved in the SMP-induced hiPSCs differentiation into neurons. Related to Figure 1.**

(A) Heatmap showing the normalized gene expression levels of selected transcripts associated with neuronal differentiation and functional activity in the process of SMP-induced hiPSCs differentiation (n=3 biological replicates per group). Z-score normalization was performed along the rows.

(B) Heatmap showing the normalized gene expression levels of selected transcripts associated with cellular cytoskeleton, cytoskeleton-associated proteins in the process of SMP-induced hiPSCs differentiation (n=3 biological replicates per group). Z-score normalization was performed along the columns.

(C) Heatmap showing the normalized gene expression levels of selected transcripts associated with ion channels assembly in the process of SMP-induced hiPSCs differentiation (n=3 biological replicates per group). Z-score normalization was performed along the rows.


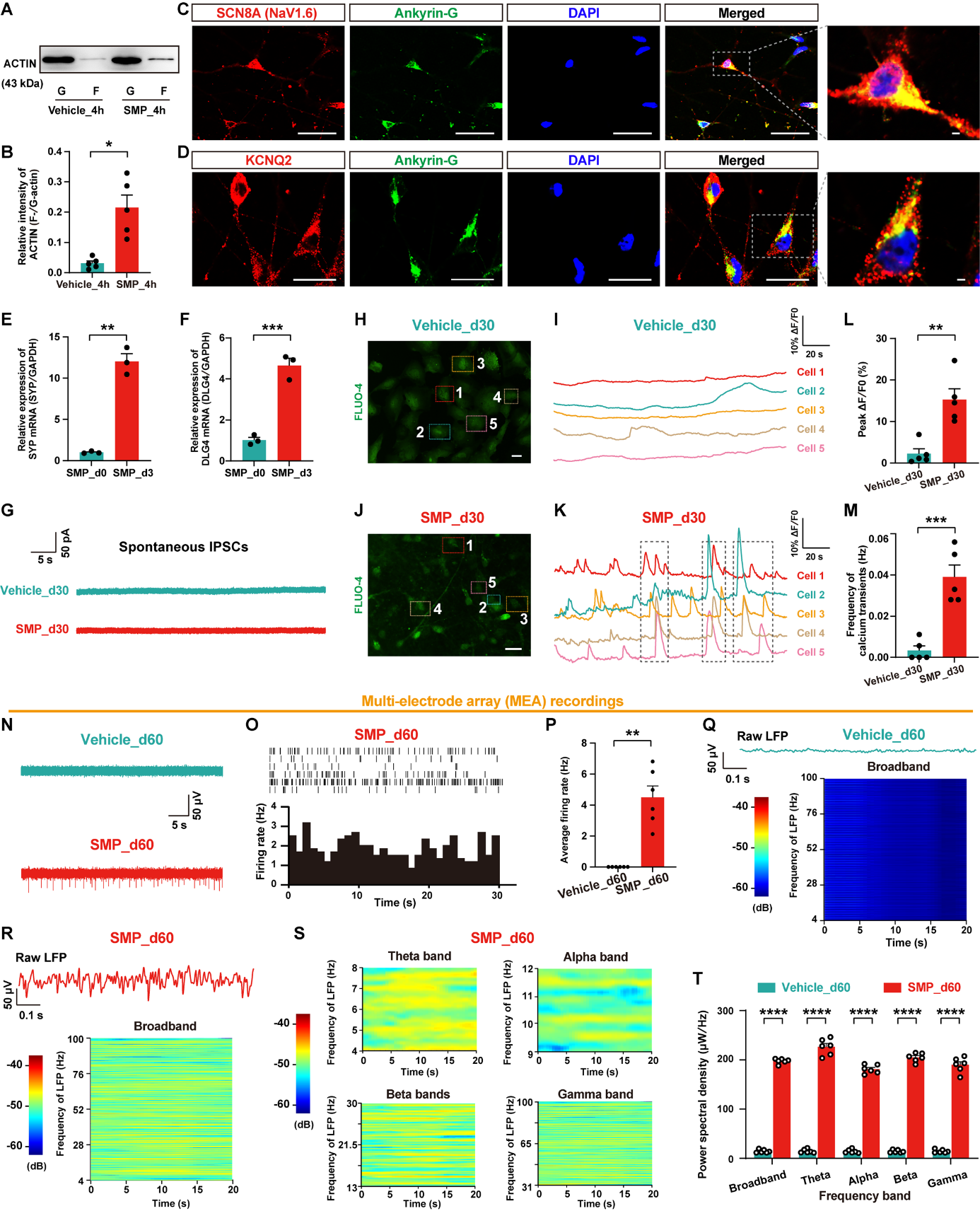


**Figure S4. Identification of the actin polymerization and the formation of synapse-like structures and neural networks of hiPSCs-derived neurons in the process of SMP-induced** **hiPSCs** **differentiation. Related to Figure 2.**

(A and B) Alteration of F-/G-actin ratio in SMP (4 hours)-induced, hiPSCs-differentiated cells using Western blot analysis. (A) Representative Western blotting bands. G, G-actin; F, F-actin. (B) Statistical analysis of F-/G-actin ratio between the ehicle_4h group and the SMP_4h group (n=5 biological replicates per group).

(C) Representative images showing the immunofluorescence staining with SCN8A (Nav 1.6) and ankyrin-G, in hiPSCs-differentiated cells cultured with the SMP for 3 days. Cell nuclei were counterstained with DAPI (blue). Scale bar=20 μm.

(D) Representative images showing the immunofluorescence staining with KCNQ2 and ankyrin-G, in hiPSCs-differentiated cells cultured with the SMP for 3 days. Cell nuclei were counterstained with DAPI (blue). Scale bar=20 μm.

(E and F) Bar graphs showing the expression of SYP (E) and DLG4 (F) mRNA levels in SMP (3 days)-induced, hiPSCs-differentiated cells using RT-qPCR analysis (n=3 biological replicates per group).

(G) Representative traces showing the spontaneous inhibitory postsynaptic currents (sIPSCs) recorded from hiPSCs-differentiated cells at 30 DIV cultured with the SMP or vehicle (n=15 cells per group).

(H) Representative images showing the dynamic calcium imaging of vehicle (30 days)-cultured, hiPSCs-differentiated cells during 80 seconds of Fluo-4 fluorescence probes treatment. The dashed boxes indicate the analyzed cells. Scale bar=20 μm.

(I) Representative traces showing the dynamic changes of relative calcium fluorescence intensity (ΔF/F0) of five selective cells in (H) during 3 minutes of Fluo-4 fluorescence probes treatment.

(J) Representative images showing the dynamic calcium imaging of SMP (30 days)-induced, hiPSCs-differentiated cells during 80 seconds of Fluo-4 fluorescence probes treatment. The dashed boxes indicate the activated cells. Scale bar=20 μm.

(K) Representative traces showing the dynamic changes of relative calcium fluorescence intensity (ΔF/F0) of five selective cells in (J) during 3 minutes of Fluo-4 fluorescence probes treatment.

(L) Bar graphs showing the peak calcium fluorescence intensity (ΔF/F0) of hiPSCs-differentiated cells at 30 DIV cultured with the SMP or vehicle (n=5 cells per group).

(M) Bar graphs showing the frequency of calcium transient in hiPSCs-differentiated cells at 30 DIV cultured with the SMP or vehicle (n=5 cells per group).

(N) Representative traces showing the spontaneous firing activity recorded from hiPSCs-differentiated cells at 60 DIV cultured with the SMP or vehicle. Scale bar: 50 μV, 5 s.

(O) Scatter diagram showing the spikes distribution of SMP-induced, hiPSCs-differentiated neurons at 60 DIV (up). Histogram showing the firing rate (Hz) of SMP-induced, hiPSCs-differentiated neurons during a time-window from the 30 s (bottom). Bin width=1 second.

(P) Bar graphs showing the average firing rate of hiPSCs-differentiated cells at 60 DIV cultured with the SMP or vehicle medium (n=6 per group).

(Q) Representative traces of the raw LFP waveform (top) and the time-varying power spectra (bottom) in the broadband (4~100 Hz) frequency range LFP of hiPSCs-differentiated cells at 60 DIV cultured with the vehicle medium. Scale bar: 50 μV, 0.1 s. Warm and cool colors indicate the increase and decrease in field potential power (FPP) activity, respectively.

(R) Representative traces of the raw LFP waveform (top) and the time-varying power spectra (bottom) in the broadband (4~100 Hz) frequency range LFP of hiPSCs-differentiated cells at 60 DIV cultured with the SMP medium. Scale bar: 50 μV, 0.1 s. Warm and cool colors indicate the increase and decrease in field potential power (FPP) activity, respectively.

(S) Representative traces of the time-varying power spectra in the theta (4~8 Hz), alpha (9~12 Hz), beta (13~30 Hz), and gamma (31~100 Hz) frequency range LFP of hiPSCs-differentiated cells at 60 DIV cultured with the SMP medium. Warm and cool colors indicate the increase and decrease in field potential power (FPP) activity, respectively.

(T) Bar graphs showing the power spectral density in the theta (4~8 Hz), alpha (9~12 Hz), beta (13~30 Hz), and gamma (31~100 Hz) frequency range LFP of hiPSCs-differentiated cells at 60 DIV cultured with the SMP or vehicle.

Data are presented as mean ± SEM. ^*^*p*<0.05; ^**^*p*<0.01; ^***^*p*< 0.001; ^****^*p*< 0.0001. Two-tailed unpaired *t* test with Welch's correction for (B), (E), (P); Two-tailed unpaired *t* test for (F), (L), (M); Repeated-measures two-way ANOVA with Sidak’s *post-hoc* test for (T).


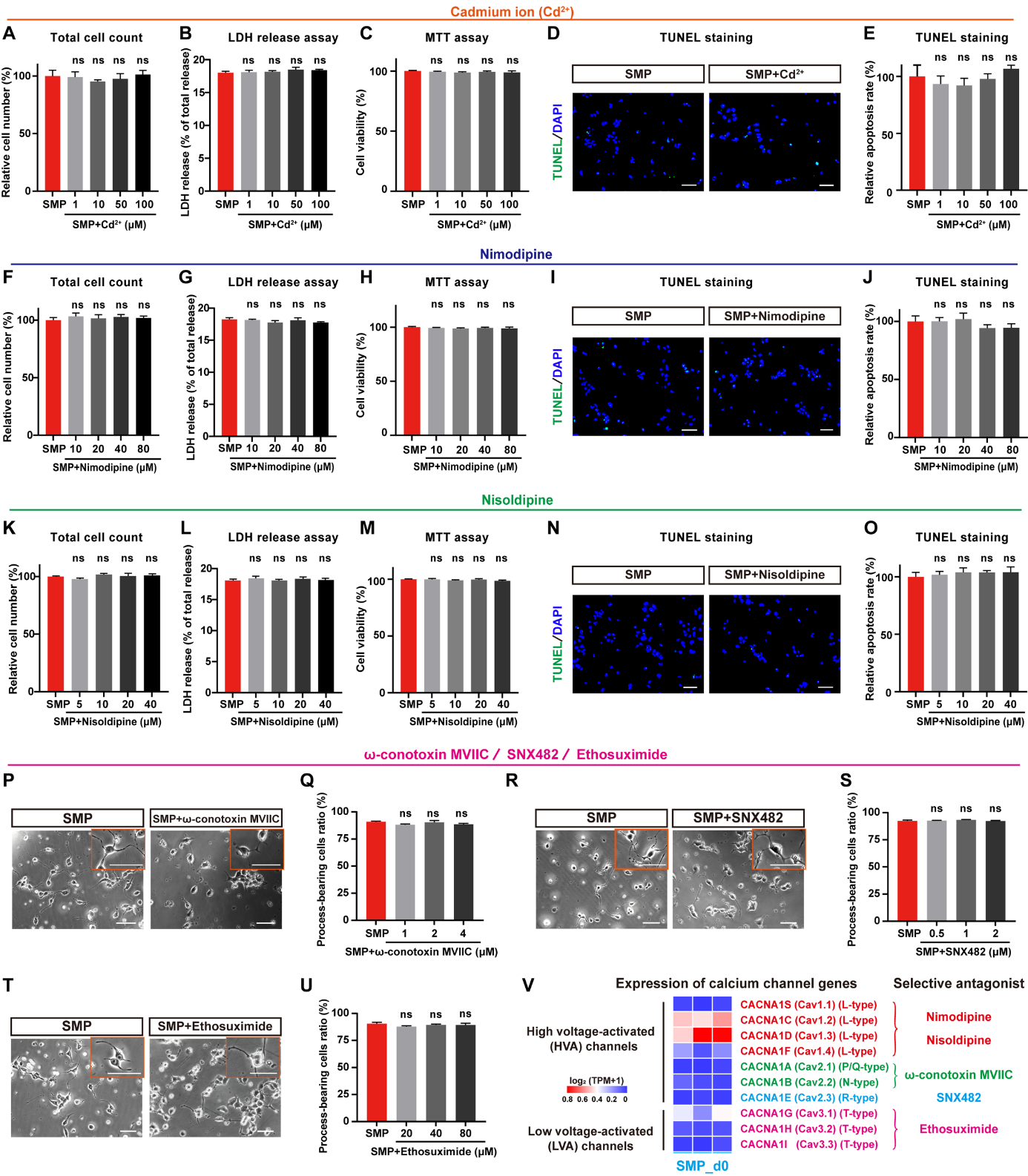


**Figure S5. Identification of L-type voltage-gated calcium channels as key players in early neuronal morphogenesis in the process of SMP-induced hiPSCs differentiation. Related to Figure 4.**

(A) Bar graph showing the statistical analysis of relative cell number after 4 hours differentiating from hiPSCs with the SMP medium containing various concentrations of Cd^2+^ (n=3 biological replicates per group).

(B) Bar graph showing the statistical analysis of LDH (lactate dehydrogenase) release (% of total release) after 4 hours differentiating from hiPSCs with the SMP medium containing various concentrations of Cd^2+^ (n=3 biological replicates per group).

(C) Bar graph showing the statistical analysis of cell viability (%) by MTT assays after 4 hours differentiating from hiPSCs with the SMP medium containing various concentrations of Cd^2+^ (n=3 biological replicates per group).

(D) Representative images showing the fluorescence staining with TUNEL (green) in hiPSCs-differentiated cells (for 4 hours), cultured with the SMP medium or SMP medium containing Cd^2+^ (100 μM) (n=3 biological replicates per group). Cell nuclei were counterstained with DAPI (blue). Scale bars=50 μm.

(E) Bar graph showing the statistical analysis of relative apoptosis rate (%) by TUNEL staining after 4 hours differentiating from hiPSCs with the SMP medium containing various concentrations of Cd^2+^ (n=3 biological replicates per group).

(F) Bar graph showing the statistical analysis of relative cell number after 4 hours differentiating from hiPSCs with the SMP medium containing various concentrations of nimodipine (n=3 biological replicates per group).

(G) Bar graph showing the statistical analysis of LDH (lactate dehydrogenase) release (% of total release) after 4 hours differentiating from hiPSCs with the SMP medium containing various concentrations of nimodipine (n=3 biological replicates per group).

(H) Bar graph showing the statistical analysis of cell viability (%) by MTT assays after 4 hours differentiating from hiPSCs with the SMP medium containing various concentrations of nimodipine (n=3 biological replicates per group).

(I) Representative images showing the fluorescence staining with TUNEL (green) in hiPSCs-differentiated cells (for 4 hours), cultured with the SMP medium or SMP medium containing nimodipine (80 μM) (n=3 biological replicates per group). Cell nuclei were counterstained with DAPI (blue). Scale bars=50 μm.

(J) Bar graph showing the statistical analysis of relative apoptosis rate (%) by TUNEL staining after 4 hours differentiating from hiPSCs with the SMP medium containing various concentrations of nimodipine (n=3 biological replicates per group).

(K) Bar graph showing the statistical analysis of relative cell number after 4 hours differentiating from hiPSCs with the SMP medium containing various concentrations of nisoldipine (n=3 biological replicates per group).

(L) Bar graph showing the statistical analysis of LDH (lactate dehydrogenase) release (% of total release) after 4 hours differentiating from hiPSCs with the SMP medium containing various concentrations of nisoldipine (n=3 biological replicates per group).

(M) Bar graph showing the statistical analysis of cell viability (%) by MTT assays after 4 hours differentiating from hiPSCs with the SMP medium containing various concentrations of nisoldipine (n=3 biological replicates per group).

(N) Representative images showing the fluorescence staining with TUNEL (green) in hiPSCs-differentiated cells (for 4 hours), cultured with the SMP medium or SMP medium containing nisoldipine (40 μM) (n=3 biological replicates per group). Cell nuclei were counterstained with DAPI (blue). Scale bars=50 μm.

(O) Bar graph showing the statistical analysis of relative apoptosis rate (%) by TUNEL staining after 4 hours differentiating from hiPSCs with the SMP medium containing various concentrations of nisoldipine (n=3 biological replicates per group).

(P) Representative phase-contrast images showing changes of cell morphology in hiPSCs-differentiated cells cultured with the SMP medium containing ω-Conotoxin MVIIC (4 μM) for 4 hours. Scale bar=50 μm.

(Q) Bar graph showing the statistical analysis of the percentage of process-bearing after 4 hours differentiating from hiPSCs with the SMP medium containing various concentration of ω-Conotoxin MVIIC (n=3 biological replicates per group).

(R) Representative phase-contrast images showing changes of cell morphology in hiPSCs-differentiated cells cultured with the SMP medium containing SNX482 (2 μM) for 4 hours. Scale bar=50 μm.

(S) Bar graph showing the statistical analysis of the percentage of process-bearing after 4 hours differentiating from hiPSCs with the SMP medium containing various concentration of SNX482 (n=3 biological replicates per group).

(T) Representative phase-contrast images showing changes of cell morphology in hiPSCs-differentiated cells cultured with the SMP medium containing ethosuximide (80 μM) for 4 hours. Scale bar=50 μm.

(U) Bar graph showing the statistical analysis of the percentage of process-bearing after 4 hours differentiating from hiPSCs with the SMP medium containing various concentration of ethosuximide (n=3 biological replicates per group).

(V) Heatmap showing the normalized gene expression levels of various voltage-gated calcium channels (VGCCs)-associated genes of hiPSCs using RNA-seq analysis. Right: the selective antagonists for different VGCC subtypes are shown. Log_2_(TPM+1) transformation was applied.

Data are presented as mean ± SEM. ns., not significant. One-way ANOVA with Dunnett’s *post-hoc* test for (A-C), (E-H), (J-M), (O), (Q), (S), (U).


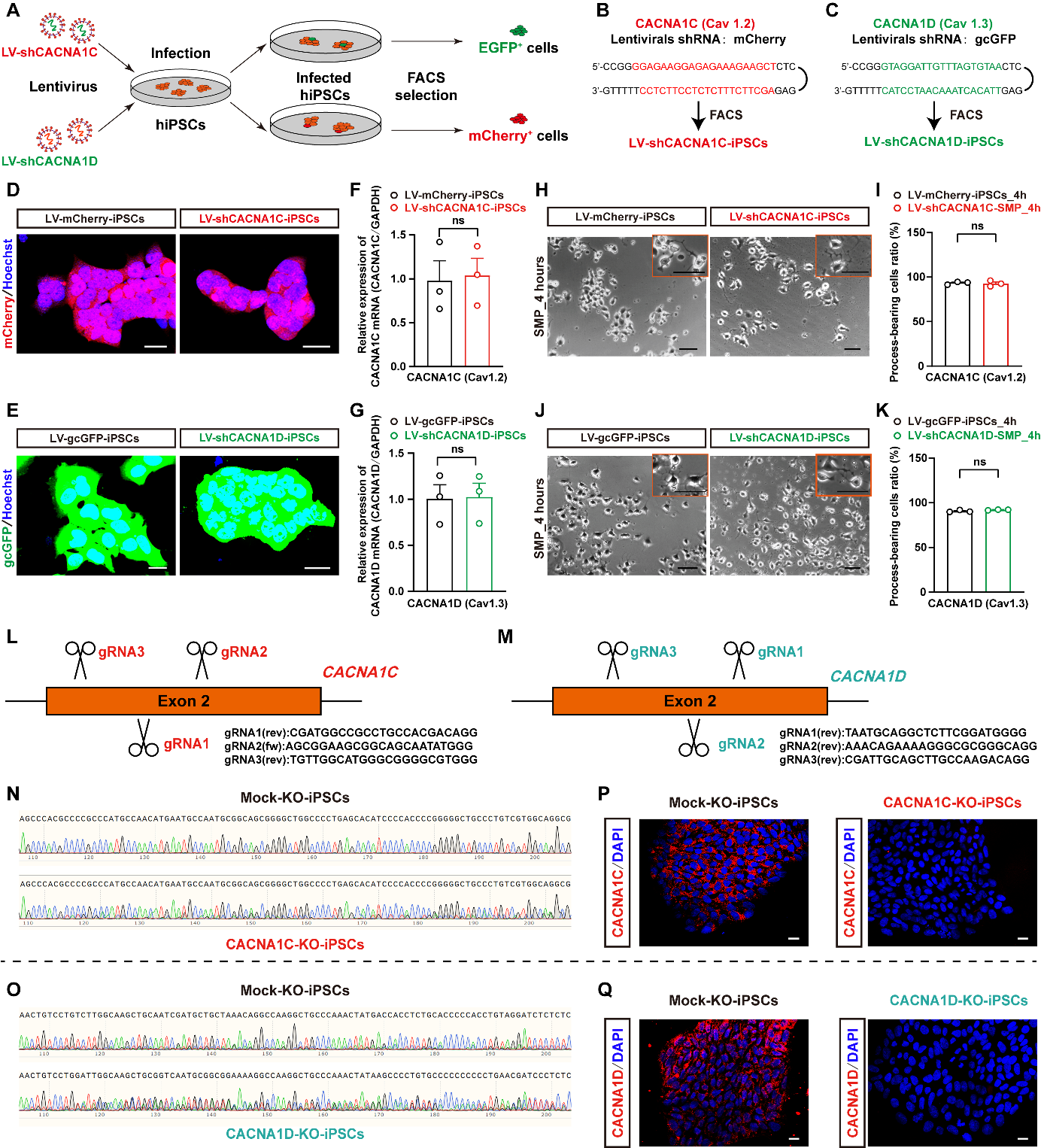


**Figure S6. Identification of Cav1.2 and Cav1.3 as key players in early neuronal morphogenesis in the process of SMP-induced hiPSCs differentiation. Related to Figure 4.**

(A) Schematic diagram depicting the experimental procedure for constructing CACNA1C or CACNA1D knockdown hiPSCs cell lines using lentivirus-expressing shRNA (LV-shRNA).

(B and C) Lentivirus infection of hiPSCs using LV-CACNA1C shRNA (B) and LV-CACNA1D shRNA (C), respectively.

(D-E) Representative images showing the expression of mCherry (D) or gcGFP (E) fluorescence protein in the live cells of lentivirus-infected hiPSCs. Cell nuclei were stained by Hoechst33342 (blue). Scale bar=20 μm.

(F-G) Bar graphs showing the expression of CACNA1C (F) or CACNA1D (G) mRNA levels between the LV-shRNA and the LV-mCherry/LV-gcGFP infected hiPSCs. (n=3 biological replicates per group).

(H) Representative phase-contrast images showing changes of cell morphology in LV-mCherry (left) and LV-shCACNA1C (right) infected hiPSCs, respectively, at 4 hours of differentiation in the SMP medium. Scale bar=50 μm.

(I) Bar graph showing the percentage of process-bearing after 4 hours differentiating from LV-shCACNA1C or LV-mCherry infected hiPSCs with the SMP medium (n=3 biological replicates per group).

(J) Representative phase-contrast images showing changes of cell morphology in LV-gcGFP (left) and LV-shCACNA1D (right) infected hiPSCs, respectively, at 4 hours of differentiation in the SMP medium. Scale bar=50 μm.

(K) Bar graph showing the percentage of process-bearing after 4 hours differentiating from LV-shCACNA1D or LV-gcGFP infected hiPSCs with the SMP medium (n=3 biological replicates per group).

(L-M) Schematic diagram illustrating the guide RNA designed for CACNA1C gene (L) and the CACNA1D gene (M), respectively.

(N) Sanger sequencing for exon 2 of the CACNA1C gene in the Mock-KO-iPSCs and the CACNA1C-KO-iPSCs.

(O) Sanger sequencing for exon 2 of the CACNA1D gene in the Mock-KO-iPSCs and the CACNA1D-KO-iPSCs.

(P) Representative images showing the immunofluorescence staining with CACNA1C in the Mock-KO-iPSCs and the CACNA1C-KO-iPSCs.

(Q) Representative images showing the immunofluorescence staining with CACNA1D in the Mock-KO-iPSCs and the CACNA1D-KO-iPSCs.

Data are presented as mean ± SEM. ns., not significant. Two-tailed unpaired *t* test for (F), (G), (I), (K).


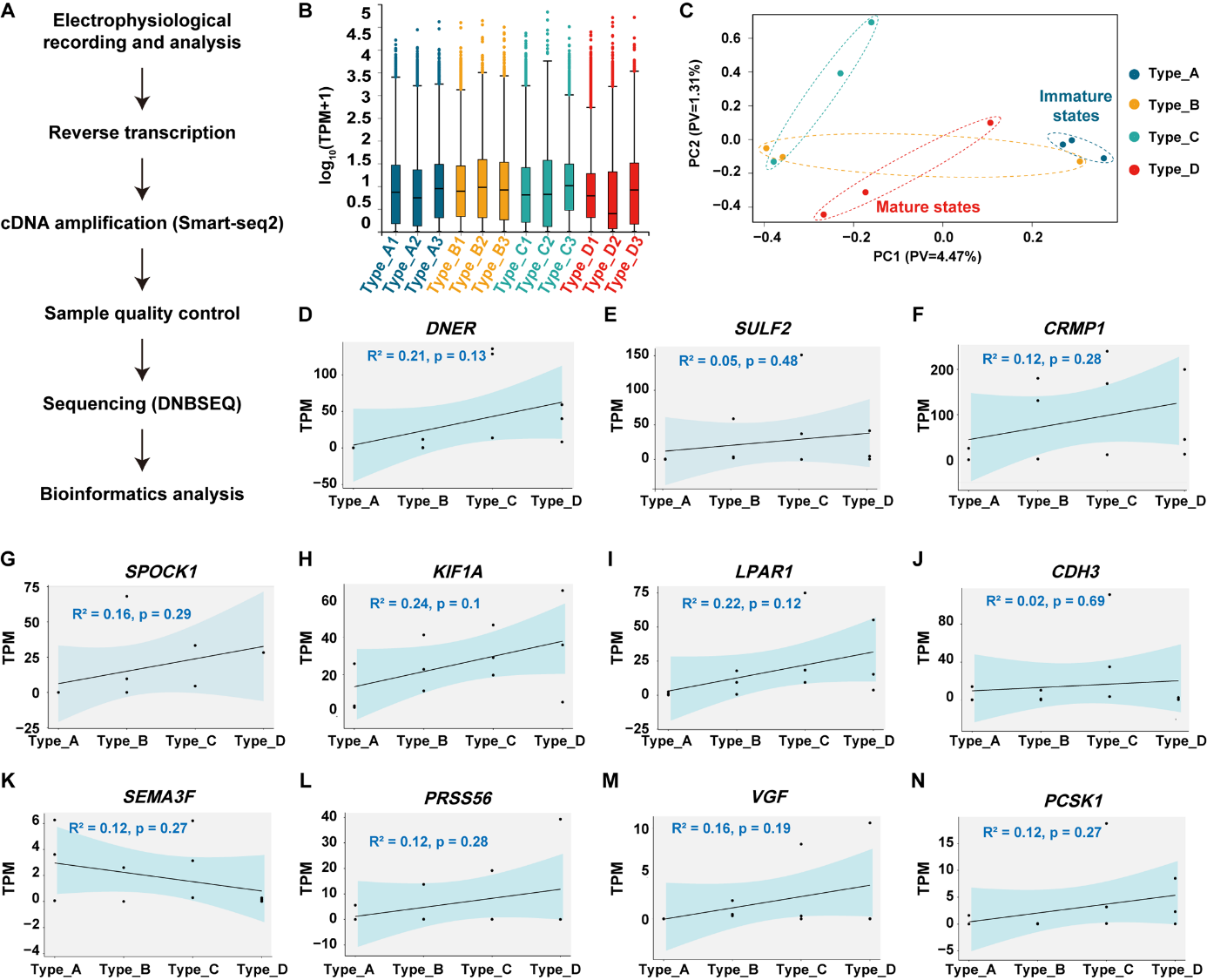


**Figure S7. Identification of key genes related to the functional developmental maturation of SMP-induced, hiPSCs-differentiated neurons using Patch-seq analysis. Related to Figure 6.**

(A) Schematic diagram of the Patch-seq experimental workflow.

(B) Boxplots showing the distribution of gene expression levels in each sample (n=3 biological replicates per group). The X-axis represents the sample names and the Y-axis represents log_10_(TPM+1). Each boxplot displays five statistics for the corresponding area: maximum, upper quartile, median, lower quartile, and minimum, excluding outliers from the upper and lower limits.

(C) Principal component analysis (PCA) of Patch-seq datasets showing the distribution of samples according to their functional maturity (n=3 biological replicates per group). PV, proportion of variance.

(D-N) Correlation analysis between the gene expression level of *DNER* (D), *SULF2* (E), *CRMP1* (F), *SPOCK1* (G), *KIF1A* (H), *LPAR1* (I), *CDH3* (J), *SEMA3F* (K), *PRSS56* (L), *VGF* (M), and *PCSK1* (N) and the neuronal functional maturity (based on AP patterns) by Patch-seq analysis (n=3 biological replicates per group). Based on the scatter plot, a regression line was plotted, and the regression model was computed to extract R-squared and *p*-values. The regression line is colored black, and the confidence interval is filled with light blue.


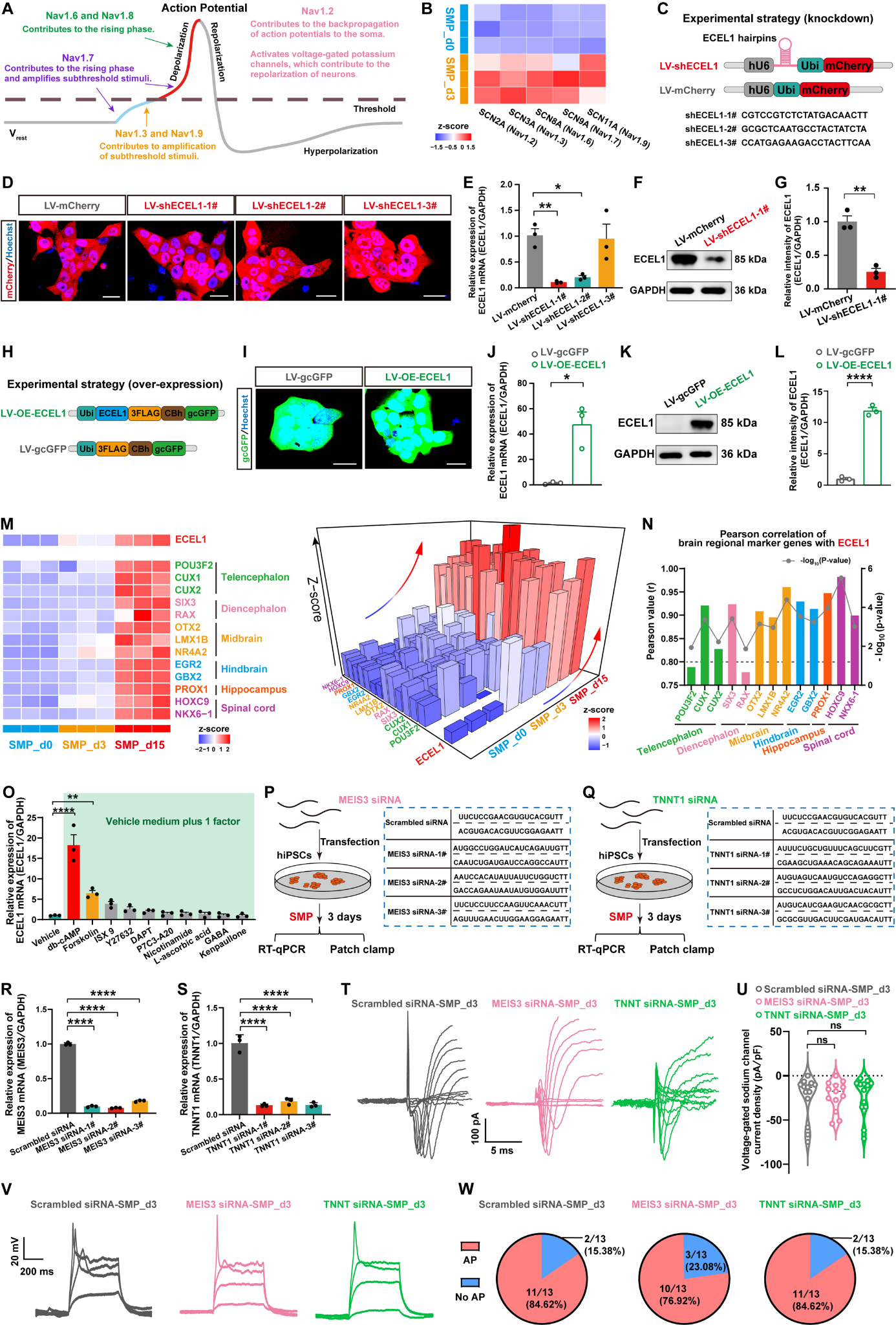


**Figure S8.** **Identification of the roles for** **ECEL1 in the functional developmental maturation of SMP-induced, hiPSCs-differentiated neurons. Related to Figure 7.**

(A) Schematic diagram depicting the involvement of multiple voltage-gated sodium ion channels (VGSCs) in various phases of action potential (AP). Referenced from David L. Bennett et al. in Physiological Reviews (2019) with minor modification.^[121]^

(B) Heatmap showing the gene expression levels of VGSCs-associated genes, including SCN2A, SCN3A, SCN8A, SCN9A, and SCN11A, between the SMP_d0 and the SMP_d3 group by RNA-seq analysis (n=3 biological replicates per group). Z-score normalization was performed along the columns.

(C) Schematic diagram illustrating the construction of recombinant lentivirus (LV) expressing three ECEL1 short hairpin RNAs (shRNAs) linked with mCherry (shRNA #1, shRNA #2, and shRNA #3).

(D) Representative images showing the expression of mCherry fluorescence protein in the live cells of LV-mCherry, LV-shRNA #1, LV-shRNA #2, and LV-shRNA #3-infected hiPSCs, respectively. Cell nuclei were stained by Hoechst33342 (blue). Scale bar=20 μm.

(E) Bar graph showing the expression of ECEL1 mRNA in the LV-shRNA (shRNA 1#, shRNA 2#, and shRNA 3#)- and the LV-mCherry infected hiPSCs (n=3 biological replicates per group).

(F-G) Western blot analyses of ECEL1 abundance between the LV-shECEL1-1#- and the LV-mCherry infected hiPSCs. (F) Representative Western blotting bands are shown. (G)Summary plot for the relative intensity of ECEL1 immunoblot (n=3 biological replicates per group).

(H) Schematic diagram depicting the construction of recombinant lentivirus (LV) expressing ECEL1 plasmid linked with gcGFP (LV-OE-ECEL1).

(I) Representative images showing the expression of gcGFP fluorescence protein in the live cells of LV-gcGFP and LV-OE-ECEL1-infected hiPSCs, respectively. Cell nuclei were stained by Hoechst33342 (blue). Scale bar=20 μm.

(J) Bar graph showing the expression of ECEL1 mRNA between the LV-OE-ECEL1- and the LV-gcGFP infected hiPSCs (n=3 biological replicates per group).

(K-L) Western blot analyses of ECEL1 abundance between the LV-OE-ECEL1- and the LV-gcGFP infected hiPSCs. (K) Representative Western blotting bands are shown. (L)Summary plot for the relative intensity of ECEL1 immunoblot (n=3 biological replicates per group).

(M) Heatmap showing the expression levels of ECEL1 and the corresponding regional markers in neural domains where ECEL1 is highly expressed, in the process of SMP-induced hiPSCs differentiation by RNA-seq analysis (n=3 biological replicates per group). Brain regional markers are classified into the following major neural domains: telencephalon (*POU3F2*, *CUX1*, *CUX2*), diencephalon​ (*SIX3*, *RAX*), midbrain (*OTX2*, *LMX1B*, *NR4A2*), hindbrain (*EGR2*, *GBX2*), hippocampal (*PROX1*), and spinal cord (*HOXC9*, *NKX6-1*). Z-score normalization was performed along the rows. The 2D heatmap is shown on the left, and the 3D heatmap is shown on the right.

(N) Histogram showing Pearson correlation analysis between ECEL1 and brain regional marker genes. The horizontal axis lists the marker genes. The left and right vertical axes display the Pearson correlation coefficient (r) and the corresponding -log_10_(*p*-value), respectively. A strong positive correlation is defined as r> 0.8. Statistical significance is indicated by -log_10_(*p*-value) > 1.3, which corresponds to *p* < 0.05.

(O) Bar graph showing the expression level of ECEL1 mRNA in hiPSCs-differentiated cells at 3 DIV cultured in “vehicle plus one factor” medium (n=3 biological replicates per group).

(P) Schematic diagram illustrating the experimental procedure for knocking down MEIS3 in hiPSCs using MEIS3 siRNA and diagram of three small interfering RNA (siRNA) sequences designed for targeting the MEIS3 gene.

(Q) Schematic diagram illustrating the experimental procedure for knocking down TNNT1 in hiPSCs using TNNT1 siRNA and diagram of three small interfering RNA (siRNA) sequences designed for targeting the TNNT1 gene.

(R) Bar graph showing the expression of MEIS3 mRNA in MEIS3 siRNA (MEIS3 siRNA-1#, MEIS3 siRNA-2#, and MEIS3 siRNA-3#)- and scramble siRNA transfected hiPSCs at day 3 culture with the SMP medium (n=3 biological replicates per group).

(S) Bar graph showing the expression of TNNT1 mRNA in TNNT1 siRNA (TNNT1 siRNA-1#, TNNT1 siRNA-2#, and TNNT1 siRNA-3#)- and scramble siRNA transfected hiPSCs at day 3 culture with the SMP medium (n=3 biological replicates per group).

(T) Representative traces showing the VGSCs currents of SMP-induced (3 DIV), hiPSCs-differentiated cells among the MEIS3 siRNA group, TNNT1 siRNA group, and the scramble siRNA group. The VGSCs currents were recorded in the voltage-clamp configuration by delivering voltage steps ranging from -80 mV to +60 mV for 100 ms, in 10 mV increments.

(U) Violin plots showing the averaged current density (pA/pF) of VGSCs of SMP-induced (3 DIV), hiPSCs-differentiated cells among between the MEIS3 siRNA group, TNNT1 siRNA group, and the scramble siRNA group (n=13 cells per group). The VGSCs currents were measured at -20 mV holding voltage.

(V) Representative traces showing the evoked action potentials of SMP-induced (3 DIV), hiPSCs-differentiated cells among the MEIS3 siRNA group, TNNT1 siRNA group, and the scramble siRNA group. Action potentials were evoked by a series of depolarizing current pulses from 0 pA to +15 pA for 600 ms, in 5 pA increments.

(W) Pie chart showing the proportion of action potential firing cells in the SMP-induced (3 DIV), hiPSCs-differentiated cells among the MEIS3 siRNA group, TNNT1 siRNA group, and the scramble siRNA group. (n=13 cells per group).

Data are presented as mean ± SEM. ^*^*p*<0.05; ^**^*p*<0.01; ^****^*p*< 0.0001. One-way ANOVA with Dunnett’s *post-hoc* test for (E), (O), (R), (S), (U); Two-tailed unpaired *t* test for (G), (L); Two-tailed unpaired *t* test with Welch's correction for (J).

**Table S1**: Key Resource Table

| **REAGENT or RESOURCE** | **SOURCE** | **IDENTIFIER** |
| --- | --- | --- |
| **Antibodies** | | |
| Rabbit monoclonal anti-CD13 | Abcam | Cat# ab108310; RRID: AB_10866195 |
| Mouse monoclonal anti-MCR | Santa Cruz | Cat# sc-53000; RRID: AB_784896 |
| Rat monoclonal anti-ZO-1 | Santa Cruz | Cat# sc-33725; RRID: AB_628459 |
| Mouse monoclonal anti-OCT-3/4 | Santa Cruz | Cat# sc-5279; RRID: AB_628051 |
| Rabbit monoclonal anti-NANOG | Abcam | Cat# ab109250; RRID: AB_10863442 |
| Mouse monoclonal anti-SOX2 | Santa Cruz | Cat# sc-365964; RRID: AB_10843364 |
| Mouse monoclonal anti-SSEA-4 | Santa Cruz | Cat# sc-21704; RRID: AB_628289 |
| Mouse monoclonal anti-TRA-1-60 | Santa Cruz | Cat# sc-21705; RRID: AB_628385 |
| Mouse monoclonal anti-TRA-1-81 | Santa Cruz | Cat# sc-21706; RRID: AB_628386 |
| Mouse monoclonal anti-NESTIN | Santa Cruz | Cat# sc-23927; RRID: AB_627994 |
| Mouse monoclonal anti-GFAP | Cell Signaling Technology | Cat# 3670; RRID: AB_561049 |
| Rabbit monoclonal anti-β3-Tubulin | Cell Signaling Technology | Cat# 5568; RRID: AB_10694505 |
| Mouse monoclonal anti-MAP2 | Invitrogen | Cat# MA5-12826; RRID: AB_10976831 |
| Mouse monoclonal anti-CD31 | Cell Signaling Technology | Cat# 3528; RRID: AB_2160882 |
| Rabbit monoclonal anti-SOX17 | Abcam | Cat# ab224637; RRID: AB_2801385 |
| Mouse monoclonal anti-Neurofilament-H | Cell Signaling Technology | Cat# 2836; RRID: AB_10694081 |
| Mouse monoclonal anti-NeuN | Millipore | Cat# MAB377; RRID: AB_2298772 |
| Rabbit monoclonal anti-VGluT1 | Abcam | Cat# ab227805; RRID: AB_2868428 |
| Mouse monoclonal anti-GAD1 | Millipore | Cat# MAB5406; RRID: AB_2278725 |
| Rabbit monoclonal anti-Anti-Choline Acetyltransferase | Abcam | Cat# ab181023; RRID: AB_2687983 |
| Mouse monoclonal anti-Tyrosine Hydroxylase | Abcam | Cat# ab137869; RRID: AB_2801410 |
| Mouse monoclonal anti-S-100 α/β | Santa Cruz | Cat# sc-58839; RRID: AB_2183338 |
| Mouse monoclonal anti-O4 | Millipore | Cat# MAB345; RRID: AB_94872 |
| Mouse monoclonal anti-Myelin Basic Protein | Abcam | Cat# ab11159; RRID: AB_297797 |
| Rabbit monoclonal anti-Actin | Abcam | Cat# ab179467; RRID: AB_2737344 |
| Mouse monoclonal anti-α-Tubulin | Cell Signaling Technology | Cat# 3873; RRID: AB_1904178 |
| Rabbit polyclonal anti-ANK3 | Elabscience | Cat# E-AB-13058 |
| Rabbit polyclonal anti-SCN8A | Alomone Labs | Cat# asc-009; RRID: AB_2040202 |
| Mouse monoclonal anti-KCNQ2 | Proteintech | Cat# 66774-1-Ig; RRID: AB_2882120 |
| Mouse monoclonal anti-Ankyrin G | Thermo Fisher Scientific | Cat# 33-8800; RRID: AB_2533145 |
| Mouse monoclonal anti-SYP | Santa Cruz | Cat# sc-17750; RRID: AB_628311 |
| Goat polyclonal anti-PSD95 | Abcam | Cat# ab12093; RRID: AB_298846 |
| Mouse monoclonal anti-CACNA1C | Abcam | Cat# ab84814; RRID: AB_1860052 |
| Mouse monoclonal anti-CACNA1D | Abcam | Cat# ab84811; RRID: AB_2259522 |
| Rabbit polyclonal anti-ECEL1 | Abcam | Cat# ab228490 |
| Rabbit polyclonal anti-Calmodulin 3 | Thermo Fisher Scientific | Cat# PA5-88172; RRID: AB_2804714 |
| Mouse monoclonal anti-CALM3 | Thermo Fisher Scientific | Cat# H00000808-M11; RRID: AB_1672296 |
| Rabbit monoclonal anti-GAPDH | Abcam | Cat# ab128915; RRID: AB_11143050 |
| Mouse (G3A1) Monoclonal Antibody IgG1 Isotype Control | Cell Signaling Technology | Cat# 5415S; RRID: AB_10829607 |
| Donkey anti-Mouse Alexa Fluor 488 | Jackson ImmunoResearch | Cat# 715-545-150; RRID: AB_2340846 |
| Donkey anti-Rabbit Alexa Fluor 488 | Jackson ImmunoResearch | Cat# 711-546-152; RRID: AB_2340619 |
| Donkey anti-Mouse Cy3 | Jackson ImmunoResearch | Cat# 715-165-150; RRID: AB_2340813 |
| Donkey anti-Rabbit Cy3 | Jackson ImmunoResearch | Cat# 711-165-152; RRID: AB_2307443 |
| Donkey anti-Goat Alexa Fluor 647 | Jackson ImmunoResearch | Cat# 705-605-147; RRID: AB_2340437 |
| HRP-labeled Goat anti-rabbit IgG (H&L) | ZSGB-Bio | Cat# ZB-2301; RRID: AB_2747412 |
| HRP-labeled Goat anti-mouse IgG (H+L) | ZSGB-Bio | Cat#: ZB-2305; RRID: AB_2747415 |
| Donkey polyclonal anti-Rat Cy3 | Beyotime | Cat# A0507; RRID: AB_3073756 |
| **Bacterial and virus strains** | | |
| Lentivirus-expressing ECEL1 | Genechem | N/A |
| Lentivirus-expressing shECEL1 | Genechem | N/A |
| Lentivirus-expressing shCACNA1C | Genechem | N/A |
| Lentivirus-expressing shCACNA1D | Genechem | N/A |
| Lentivirus-expressing mCherry | Genechem | N/A |
| Lentivirus-expressing gcGFP | Genechem | N/A |
| Lentivirus-expressing CALM3 | Genechem | N/A |
| **Biological samples** | | |
| Primary human urinary-derived epithelial cells | This paper | N/A |
| **Chemicals, peptides, and recombinant proteins** | | |
| Phosphate Buffered saline (PBS) | Gibco | Cat# 10010023 |
| Penicillin/Streptomycin | Gibco | Cat# 10378016 |
| Gelatin | MREDA | Cat# M050253 |
| Dulbecco's Modified Eagle Medium/Nutrient Mixture F-12 (DMEM/F12) | Gibco | Cat# 11330032 |
| Fetal Bovine Serum (FBS) | ScienProCell | Cat# FBS-SPC900A |
| Primocin Antimicrobial Agent For Primary Cells | InvivoGen | Cat# ant-pm-1 |
| Holo-Transferrin | Macgene | Cat# CC110 |
| Sodium Selenite | Macgene | Cat# CC111 |
| Insulin | Wisent | Cat# 511-016-CM |
| Hydrocortisone | Macgene | Cat# CC103 |
| L-Epinephrine | MedChemExpress | Cat# HY-B0447B |
| L-Thyroxin | Maya Reagent | Cat# 61512 |
| Recombinant Human Epidermal Growth Factor (EGF) | Novoprotein | Cat# C029 |
| Matrigel hESC-Qualified Matrix | Corning | Cat# 354277 |
| TrypLE Express Enzyme | Gibco | Cat# 12604013 |
| Opti-MEM Reduced Serum Medium | Gibco | Cat# 11058021 |
| Recombinant Human Fibroblast Growth Factor 2 (FGF-2) | Novoprotein | Cat# C779 |
| N-2 Supplement | Gibco | Cat# 17502048 |
| B-27 Supplement | Gibco | Cat# 17504044 |
| L-Glutamine | Gibco | Cat# 25030149 |
| Non Essential Amino Acids (NEAA) | Millipore | Cat# TMS-001-C |
| 2-Mercaptoethanol | Sigma-Aldrich | Cat# M3148 |
| Sodium Pyruvate | Gibco | Cat# 11360070 |
| L-Ascorbic Acid | Sigma-Aldrich | Cat# A4544 |
| Dimethyl sulfoxide (DMSO) | Sigma-Aldrich | Cat# D4540 |
| PD0325901 | Targetmol | Cat# T6189 |
| CHIR99021 | Abcam | Cat# ab120890 |
| A-83-01 | Targetmol | Cat# T3031 |
| HA-100 | MedChemExpress | Cat# HY-100984 |
| Recombinant Human Leukemia Inhibitory Factor (LIF) | Novoprotein | Cat# C017 |
| mTeSR1 Medium | STEMCELL Technologies | Cat# 85850 |
| Accutase | STEMCELL Technologies | Cat# 07920 |
| PSCeasy Cell Dissociation Solution | Cellapybio | Cat# CA3001500 |
| 4% Paraformaldehyde (PFA) Fix Solution | Beyotime | Cat# P0099 |
| Triton X-100 | Sigma-Aldrich | Cat# X100-500ML |
| Bovine Serum Albumin (BSA) | Sigma-Aldrich | Cat# A9647 |
| 4',6-diamidino-2-phenylindole (DAPI) | Sigma-Aldrich | Cat# P3761 |
| Mounting Medium, antifading | Solarbio | Cat# S2100 |
| TRIzol Reagent | Invitrogen | Cat# 15596026 |
| Agarose | BIOWEST | Cat# 111860 |
| Gel-Red | Beyotime Biotechnology | Cat# D0140 |
| Trans DNA Marker I | TransGen Biotech | Cat# BM401 |
| Knockout™ Serum Replacement | Gibco | Cat# 10828010 |
| Neurobasal Medium | Gibco | Cat# 21103049 |
| Y-27632 | Targetmol | Cat# T1725 |
| Forskolin | APExBIO | Cat# B1421 |
| ISX 9 | APExBIO | Cat# B5596 |
| Dibutyryl-cAMP, sodium salt | APExBIO | Cat# B9001 |
| DAPT | Targetmol | Cat# T6202 |
| Kenpaullone | MedChemExpress | Cat# HY-12302 |
| P7C3-A20 | Targetmol | Cat# T2423 |
| GABA | Leyan | Cat# 1035955 |
| Nicotinamide | Yuanye Bio-Technology | Cat# S13015 |
| Purmorphamine | Stemcell | Cat# 72202 |
| Retinoic acid | Sigma-Aldrich | Cat# R2625 |
| Dorsomorphin | Targetmol | Cat# T6146 |
| LDN-193189 | Targetmol | Cat# T1935 |
| SB431542 | Targetmol | Cat# T1726 |
| Go 6983 | Targetmol | Cat# T6313 |
| Valproic acid | MedChemExpress | Cat# HY-10585 |
| RepSox | Targetmol | Cat# T6337 |
| XAV939 | HARVEYBIO | Cat# X32354 |
| SP600125 | MedChemExpress | Cat# HY-12041 |
| Recombinant Human Brain-Derived Neurotrophic Factor (BDNF) | Novoprotein | Cat# C076 |
| Recombinant Human Glial Cell Line-Derived Neurotrophic Factor (GDNF) | R&D SYSTEM | Cat# 512-GF-01M |
| Cholera toxin | Macgene | Cat# CC104 |
| Recombinant Human Activin A | PeproTech | Cat# 120-14-2 |
| Recombinant Human Noggin | Novoprotein | Cat# 120-10C |
| Laminin | Corning | Cat# 354232 |
| Recombinant Human SHH | Novoprotein | Cat# C100 |
| Recombinant Human CNTF | Novoprotein | Cat# C098 |
| Recombinant Human IGF-I | Novoprotein | Cat# C031 |
| Recombinant Human β-NGF | PeproTech | Cat# 450-01-20 |
| Recombinant Human NT-3 | PeproTech | Cat# 450-03 |
| Potassium Gluconate (K-gluconate) | Sigma-Aldrich | Cat# G4500 |
| 4-(2-Hydroxyethyl)piperazine-1-ethanesulfonic acid (HEPES) | Sigma-Aldrich | Cat# H3375 |
| Potassium Chloride (KCl) | Sigma-Aldrich | Cat# P3911 |
| Ethylene Glycol Tetraacetic Acid (EGTA) | Sigma-Aldrich | Cat# 324626 |
| Guanosine 5’-triphosphate sodium salt hydrate (Na_2_GTP) | Sigma-Aldrich | Cat# 51120 |
| Adenosine 5′-triphosphate magnesium salt (MgATP) | Sigma-Aldrich | Cat# A9187 |
| Potassium Hydroxide (KOH) | TGREAG | Cat# 109029 |
| Sodium Chloride (NaCl) | Sigma-Aldrich | Cat# S9888 |
| Calcium Chloride Dihydrate (CaCl_2_·2H_2_O) | Sigma-Aldrich | Cat# 12022 |
| Magnesium Sulfate Heptahydrate (MgSO_4_·7H_2_O) | Sigma-Aldrich | Cat# PHR2604 |
| Sodium Phosphate monobasic (NaH_2_PO_4_) | Sigma-Aldrich | Cat# S3139 |
| D-glucose | Sigma-Aldrich | Cat# G7021 |
| Sodium Hydroxide (NaOH) | TGREAG | Cat# 109026 |
| Bicuculline | YUANYE | Cat# B20089 |
| Cesium methanesulfonate | Sigma-Aldrich | Cat# C1426 |
| Sodium methanesulfonate | Aladdin | Cat# S161234 |
| Caesium hydroxide | Sigma-Aldrich | Cat# 232041 |
| CNQX | Sigma-Aldrich | Cat# C127 |
| D-AP5 | Tocris | Cat# 0106 |
| SPI-Chem™ Glutaraldehyde 25% EM Grade | Structure Probe, Inc. | Cat# 02607 |
| Phalloidin-iFluor 647 Reagent | Abcam | Cat# ab176759 |
| Fluorescent Deoxyribonuclease I Conjugates | Molecular Probes, Inc. | Cat# D12371 |
| Radioimmunoprecipitation Assay (RIPA) Lysis Buffer | Applygen | Cat# C1053+ |
| Protease and phosphatase inhibitor cocktail for general use | Beyotime | Cat# P1045 |
| Guanidine Hydrochloride | Macklin | Cat# G810478 |
| Sodium Acetate | Macklin | Cat# S818277 |
| Tris-HCl | Macklin | Cat# T917654 |
| Tween-20 | Biobying Biotech | Cat# 0777 |
| ECL (enhanced chemiluminescence) Reagents | Tanon | Cat# 180-5001 |
| Fura-2, AM | Beyotime | Cat# S1052 |
| Ionomycin | Macklin | Cat# I838446 |
| Thapsigargin | Macklin | Cat# T863962 |
| Fluo-4, AM | Thermo Fisher Scientific | Cat# F14217 |
| BrainPhys™ Neuronal Medium | Stemcell | Cat# 05790 |
| Coomassie Brilliant Blue R250 | Leyan | Cat# 1080151 |
| Methanol | TGREAG | Cat# 12393 |
| Acetic acid | TGREAG | Cat# 12002 |
| Arachidonic acid | MedChemExpress | Cat# HY-109590 |
| ML-7 hydrochloride | APExBIO | Cat# A3626 |
| Ionomycin | Maclin | Cat# I838446 |
| Thapsigargin | Maclin | Cat# T863962 |
| Cadmium standard solution | Nacalai Tesque | Cat# L5214 |
| Nimodipine | BIOBYING | Cat# BY-N78560 |
| Nisoldipine | Aladdin | Cat# N125589 |
| ω-Conotoxin MVIIC | Aladdin | Cat# C274837 |
| SNX 482 | Aladdin | Cat# ab120259 |
| Ethosuximide | Macklin | Cat# E838503 |
| Hoechst 33342 Staining Dye Solution | Abcam | Cat# ab228551 |
| Lipofectamine^TM^ 3000 reagent | Invitrogen | Cat# L3000008 |
| DNA-OFF | Takara | Cat# 9036 |
| RNaseZap | Thermo Fisher Scientific | Cat# AM9780 |
| Glycogen | Sigma-Aldrich | Cat# G0885 |
| Recombinant RNase inhibitor | Takara | Cat# 2313A |
| INTERFERin® Reagent | Polyplus | Cat# 101000028 |
| **Critical Commercial Assays** | | |
| BCIP/NBT Alkaline Phosphatase Color Development Kit | Beyotime | Cat# C3206 |
| PrimeScript™ 1st Strand cDNA Synthesis Kit | Takara | Cat# 6110A |
| TaKaRa Taq™ Kit | Takara | Cat# R001A |
| GoTaq® qPCR Master Mix | Promega | Cat# A6001 |
| TIANamp Genomic DNA Kit | TIANGEN | Cat# DP304 |
| DNA Bisulfite Conversion Kit | TIANGEN | Cat# DP215 |
| TaKaRa LA Taq® Hot Start Version | Takara | Cat# RR042A |
| pMD™18-T Vector Cloning Kit | Takara | Cat# 6011 |
| One Step SDS-PAGE Gel Fast Preparation Kit | Biotides | Cat# WB2102 |
| LDH Release Assay Kit | Beyotime | Cat# C0017 |
| MTT Cell Proliferation and Cytotoxicity Assay Kit | Beyotime | Cat# C0009M |
| One Step TUNEL Apoptosis Assay Kit | Beyotime | Cat# C1088 |
| Pierce™ Co-Immunoprecipitation Kit | Thermo Fisher Scientific | Cat# 26149 |
| **Deposited data** | | |
| Raw bulk RNA-Seq data | This paper | Table S5_Source data: Figure S2A |
| Raw Patch-Seq data | This paper | Table S5_Source data: Figure S7B |
| **Experimental Models: Cell Lines** | | |
| Human: H1 embryonic stem cell line | WiCell | Cat# WA01, RRID: CVCL_9771 |
| Human: H9 embryonic stem cell line | WiCell | Cat# WA09, RRID: CVCL_9773 |
| **Experimental Models: Organisms/Strains** | | |
| Mus musculus (mouse): BALB/c Nude­­­ | Charles River | N/A |
| **Oligonucleotides** | | |
| Primers used for PCR | This paper | Table S2 |
| Short hairpin RNA (shRNA) | This paper | Table S3 |
| Guide RNA (gRNA) | This paper | Table S3 |
| Small interfering RNA (siRNA) | This paper | Table S3 |
| **Recombinant DNA** | | |
| pCXLE-hOCT3/4-shp53-F | Addgene | Cat# 27077 |
| pCXLE-hSK | Addgene | Cat# 27078 |
| pCXLE-hUL | Addgene | Cat# 27080 |
| pCXWB-EBNA1 | Addgene | Cat# 37624 |
| CRISPR-Cas9 plasmid vector | Guangzhou Ubigene Co., LTD. | Cat# YKO-RP003 |
| **Software and Algorithms** | | |
| GraphPad Prism 9.0 | GraphPad Software | https://www.graphpad.com/scientific-software/_main.html#physiol_epc10single |
| Leica LAS X 3.0 | Leica | https://www.leica-microsystems.lifescience.com/en/downloads/lifescience.com/en/downloads/oads_main.html#down_patchmaster |
| Patch-Master software | HEKA | http://www.heka.com/downloads/downloads_main.html#down_patchmaster |
| Origin 2024 | OriginLab | https://www.originlab.com/ |
| ImageJ | NIH | https://imagej.nih.gov/ij/ |
| R version 4.3.3 | R-Project | https://www.r-project.org/software/ |
| STRING 12.0 | STRING | https://cn.string-db.org/ |
| Multi-omics interaction system | BGI | https://biosys.bgi.com/ |
| NeuroExplorer 5 | Nex Technologies | https://www.neuroexplorer.com/support/version5/ |

**Table S2**. PCR primer sequences.

| **Genes** | **Direction** | **Sequence** | **Applications** |
| --- | --- | --- | --- |
| *Endo OCT3/4* | Forward | 5'-GAC AGG GGG AGG GGA GGA GCT AGG-3' | Endo *OCT3/4* RT-PCR |
|  | Reverse | 5'-CTT CCC TCC AAC CAG TTG CCC CAA AC-3' |  |
| *Endo SOX2* | Forward | 5'-GGG AAA TGG GAG GGG TGC AAA AGA GG-3' | Endo *SOX2* RT-PCR |
|  | Reverse | 5'-TTG CGT GAG TGT GGA TGG GAT TGG TG-3' |  |
| *NANOG* | Forward | 5'-CAG CCC CGA TTC TTC CAC CAG TCC C-3' | *NANOG* RT-PCR |
|  | Reverse | 5'-CGG AAG ATT CCC AGT CGG GTT CAC C-3' |  |
| *REX1* | Forward | 5'-CAG ATC CTA AAC AGC TCG CAG AAT-3' | *REX1* RT-PCR |
|  | Reverse | 5'-GCG TAC GCA AAT TAA AGT CCA GA-3' |  |
| *DPPA5* | Forward | 5'-ATA TCC CGC CGT GGG TGA AAG TTC-3' | *DPPA5* RT-PCR |
|  | Reverse | 5'-ACT CAG CCA TGG ACT GGA GCA TCC-3' |  |
| *DNMT3B* | Forward | 5'-TGC TGC TCA CAG GGC CCG ATA CTT C-3' | *DNMT3B* RT-PCR |
|  | Reverse | 5'-TCC TTT CGA GCT CAG TGC ACC ACA AAA C-3' |  |
| *ACTB* | Forward | 5'-TGA AGT GTG ACG TGG ACA TC-3' | *ACTB* RT-PCR |
|  | Reverse | 5'-GGA GGA GCA ATG ATC TTG AT-3' |  |
| promoter region of *OCT3/4* | Forward | 5'-GAG GTT GGA GTA GAA GGA TTG TTT TGG TTT-3' | Bisulfite sequencing |
|  | Reverse | 5'-CCC CCC TAA CCC ATC ACC TCC ACC ACC TAA-3' |  |
| promoter region of *NANOG* | Forward | 5'-TGG TTA GGT TGG TTT TAA ATT TTT G-3' | Bisulfite sequencing |
|  | Reverse | 5'-AAC CCA CCC TTA TAA ATT CTC AAT TA-3' |  |
| *MAP2* | Forward | 5'-CAG GTG GCG GAC GTG TGA AAA TTG AGA GTG-3' | *MAP2* RT-PCR |
|  | Reverse | 5'-CAC GCT GGA TCT GCC TGG GGA CTG TG-3' |  |
| *NEUROD1* | Forward | 5'-AAG CCA TGA ACG CAG AGG AGG ACT-3' | *NEUROD1* RT-PCR |
|  | Reverse | 5'-AGC TGT CCA TGG TAC CGT AA-3' |  |
| *CK8* | Forward | 5'-CCT GGA AGG GCT GAC CGA CGA GAT CAA-3' | *CK8* RT-PCR |
|  | Reverse | 5'-CTT CCC AGC CAG GCT CTG CAG CTC C-3' |  |
| *GFAP* | Forward | 5'-GGC CCG CCA CTT GCA GGA GTA CCA GG-3' | *GFAP* RT-PCR |
|  | Reverse | 5'-CTT CTG CTC GGG CCC CTC ATG AGA CG-3' |  |
| *MSX1* | Forward | 5'-CGA GAG GAC CCC GTG GAT GCA GAG-3' | *MSX1* RT-PCR |
|  | Reverse | 5'-GGC GGC CAT CTT CAG CTT CTC CAG-3' |  |
| *BRACHYURY* | Forward | 5'-GCC CTC TCC CTC CCC TCC ACG CAC AG-3' | *BRACHYURY* RT-PCR |
|  | Reverse | 5'-CGG CGC CGT TGC TCA CAG ACC ACA GG-3' |  |
| *SOX17* | Forward | 5'-CGC TTT CAT GGT GTG GGC TAA GGA CG-3' | *SOX17* RT-PCR |
|  | Reverse | 5'-TAG TTG GGG TGG TCC TGC ATG TGC TG-3' |  |
| *AFP* | Forward | 5'-GAA TGC TGC AAA CTG ACC ACG CTG GAA C-3' | *AFP* RT-PCR |
|  | Reverse | 5'-TGG CAT TCA AGA GGG TTT TCA GTC TGG A-3' |  |
| *SYP* | Forward | 5'-TCG GCT TTG TGA AGG TGC TGC A-3' | *SYP* RT-qPCR |
|  | Reverse | 5'-TCA CTC TCG GTC TTG TTG GCA C-3' |  |
| *DLG4* | Forward | 5'-TCC ACT CTG ACA GTG AGA CCG A-3' | *DLG4* RT-qPCR |
|  | Reverse | 5'-CGT CAC TGT CTC GTA GCT CAG A-3' |  |
| *TUBB3* | Forward | 5'-TCA GCG TCT ACT ACA ACG AGG C-3' | *TUBB3* RT-qPCR |
|  | Reverse | 5'-GCC TGA AGA GAT GTC CAA AGG C-3' |  |
| *MAP2* | Forward | 5'-AGG CTG TAG CAG TCC TGA AAG G-3' | *MAP2* RT-qPCR |
|  | Reverse | 5'-CTT CCT CCA CTG TGA CAG TCT G-3' |  |
| *CACNA1C* | Forward | 5'-GCA GGA GTA CAA GAA CTG TGA GC-3' | *CACNA1C* RT-qPCR |
|  | Reverse | 5'-CGA AGT AGG TGG AGT TGA CCA C-3' |  |
| *CACNA1D* | Forward | 5'-CTT CGA CAA CGT CCT CTC TGC T-3' | *CACNA1D* RT-qPCR |
|  | Reverse | 5'-GCC GAT GTT CTC TCC ATT CGA G-3' |  |
| *CACNA1C-*Mutation | Forward | 5'-AAC CGC CTG CAA TAG CTT GA-3' | Target DNA sequencing |
|  | Reverse | 5'-CAG CCC GGT GAA CAA AGG ATA-3' |  |
| *CACNA1D-*Mutation | Forward | 5'-CTC CTA CCC ACT GGA TCC TCA-3' | Target DNA sequencing |
|  | Reverse | 5'-CTC GCA TTC CAG ACT GCT ACA-3' |  |
| *GAPDH* | Forward | 5'-GTC TCC TCT GAC TTC AAC AGC G-3' | *GAPDH* RT-qPCR |
|  | Reverse | 5'-ACC ACC CTG TTG CTG TAG CCA A-3' |  |
| *ECEL1* | Forward | 5'-TCC AGG AAG ACT TCT CGG AGG A-3' | *ECEL1* RT-qPCR |
|  | Reverse | 5'-ACA GGT GCT CAC TCA GAA CCA C-3' |  |
| *SCN2A* | Forward | 5'-CTA GCC TCA CTG TGA CAG TAC C-3' | *SCN2A* RT-qPCR |
|  | Reverse | 5'-TCA ACC GTG CTG CCT TCA GAT G-3' |  |
| *SCN3A* | Forward | 5'-CGT CAC CTA CTG GAC AAC TTC C-3' | *SCN3A* RT-qPCR |
|  | Reverse | 5'-TCA CGG CTC TTT GCC TTC CAG A-3' |  |
| *SCN9A* | Forward | 5'-GTG GAA GGA TTG TCA GTT CTG CG-3' | *SCN9A* RT-qPCR |
|  | Reverse | 5'-GCC AAC ACT AAG GTG AGG TTA CC-3' |  |
| *MEIS3* | Forward | 5'-ATC ATG CGA GCC TGG TTG TTC C-3' | *MEIS3* RT-qPCR |
|  | Reverse | 5'-CAT AGG TTG CAC GAT GCG TCT C-3' |  |
| *TNNT1* | Forward | 5'- AAC GCG AAC GTC AGG CTA AGC T -3' | *TNNT1* RT-qPCR |
|  | Reverse | 5'- CTT GAC CAG GTA GCC GCC AAA A -3' |  |
| *CALM3* | Forward | 5'-GAG AGG CGT TCC GTG TCT TTG A-3' | *CALM3* RT-qPCR |
|  | Reverse | 5'-ACC TCC TCA TCG GTC AGC TTC T-3' |  |

**Table S3.** Short hairpin RNA (shRNA), guide RNA (gRNA) and small interfering RNA (siRNA) sequences.

| **Name** | **Direction** | **Sequence** |
| --- | --- | --- |
| *CACNA1C*-shRNA | Forward | 5'-GGAGAAGGAGAGAAAGAAGCT-3' |
|  | Reverse | 5'-AGCTTCTTTCTCTCCTTCTCC-3' |
| *CACNA1D*-shRNA | Forward | 5'-GTAGGATTGTTTAGTGTAA-3' |
|  | Reverse | 5'-TTACACTAAACAATCCTAC-3' |
| *ECEL1*-shRNA-1# | Forward | 5'-CGTCCGTCTCTATGACAACTT-3' |
|  | Reverse | 5'- AAGTTGTCATAGAGACGGACG-3' |
| *ECEL1*-shRNA-2# | Forward | 5'-GCGCTCAATGCCTACTATCTA-3' |
|  | Reverse | 5'- TAGATAGTAGGCATTGAGCGC-3' |
| *ECEL1*-shRNA-3# | Forward | 5'-CCATGAGAAGACCTACTTCAA-3' |
|  | Reverse | 5'- TTGAAGTAGGTCTTCTCATGG-3' |
| *CACNA1C*-gRNA-1 | Reverse | 5'-CGATGGCCGCCTGCCACGACAGG-3' |
| *CACNA1C*-gRNA-2 | Forward | 5'-AGCGGAAGCGGCAGCAATATGGG-3' |
| *CACNA1C*-gRNA-3 | Reverse | 5'-TGTTGGCATGGGCGGGGCGTGGG-3' |
| *CACNA1D*-gRNA-1 | Reverse | 5'-TAATGCAGGCTCTTCGGATGGGG-3' |
| *CACNA1D*-gRNA-2 | Reverse | 5'-AAACAGAAAAGGGCGCGGGCAGG-3' |
| *CACNA1D*-gRNA-3 | Reverse | 5'-CGATTGCAGCTTGCCAAGACAGG-3' |
| Scrambled siRNA | Forward | 5'-UUCUCCGAACGUGUCACGUTT-3' |
|  | Reverse | 5'-ACGUGACACGUUCGGAGAATT-3' |
| *MEIS3* siRNA-1# | Sense strand | 5'-AUGGCCUGGAUCAUCAGAUUGTT-3' |
|  | Antisense strand | 5'-CAAUCUGAUGAUCCAGGCCAUTT-3' |
| *MEIS3* siRNA-2# | Sense strand | 5'-AAUCCACAUAUUAUUCUGGUCTT-3' |
|  | Antisense strand | 5'-GACCAGAAUAAUAUGUGGAUUTT-3' |
| *MEIS3* siRNA-3# | Sense strand | 5'-UUCUCCUUCCAAGUUCAAACUTT-3' |
|  | Antisense strand | 5'-AGUUUGAACUUGGAAGGAGAATT-3' |
| *TNNT1* siRNA-1# | Sense strand | 5'-AUUUCUGCUGUUUCAGCUUCGTT-3' |
|  | Antisense strand | 5'-CGAAGCUGAAACAGCAGAAAUTT-3' |
| *TNNT1* siRNA-2# | Sense strand | 5'-AUGUAGUCAAUGUCCAGAGGCTT-3' |
|  | Antisense strand | 5'-GCCUCUGGACAUUGACUACAUTT-3' |
| *TNNT1* siRNA-3# | Sense strand | 5'-AUGUCAUCGAAGUCAACGCGCTT-3' |
|  | Antisense strand | 5'-GCGCGUUGACUUCGAUGACAUTT-3' |
| *CALM3* siRNA-1# | Sense strand | 5'-CGUCACGUAAUGACGAACCUGTT-3' |
|  | Antisense strand | 5'-CAGGUUCGUCAUUACGUGACGTT-3' |
| *CALM3* siRNA-2# | Sense strand | 5'-CCAGGUCAAUUAUGAAGAGUUTT-3' |
|  | Antisense strand | 5'-AACUCUUCAUAAUUGACCUGGTT-3' |
| *CALM3* siRNA-3# | Sense strand | 5'-ACAAGGAUGGAGAUGGCACUATT-3' |
|  | Antisense strand | 5'-UAGUGCCAUCUCCAUCCUUGUTT-3' |

**Table S4.** Statistical analyses, related to Figures 1-8, and Figures S1-S8.

| **Figure** | **Conditions** | **Analysis** | **P value** | **t or F value** |
| --- | --- | --- | --- | --- |
| 1J | SMP-Nav *vs.* Vehicle-Nav | Repeated-measures two-way ANOVA with Sidak’s *post-hoc* test | *p*=0.0006 | F _(1, 14)_ = 19.12 |
|  | SMP-Kv *vs.* Vehicle-Kv | Repeated-measures two-way ANOVA with Sidak’s *post-hoc* test | *p*=0.0016 | F _(1, 14)_ = 15.21 |
|  | SMP-Nav *vs.* SMADi-NaV | Repeated-measures two-way ANOVA with Sidak’s *post-hoc* test | *p*=0.0025 | F _(1, 14)_ = 13.45 |
|  | SMP-Kv *vs.* SMADi-Kv | Repeated-measures two-way ANOVA with Sidak’s *post-hoc* test | *p*=0.0384 | F _(1, 14)_ = 5.221 |
| 1L | SMP *vs.* Vehicle | Repeated-measures two-way ANOVA with Sidak’s *post-hoc* test | *p*<0.0001 | F _(1, 16)_ = 568.9 |
|  | SMP *vs.* SMADi | Repeated-measures two-way ANOVA with Sidak’s *post-hoc* test | *p*<0.0001 | F _(1, 16)_ = 275.6 |
| 2B | SMP_1h *vs.* Vehicle_1h | Repeated-measures two-way ANOVA with Sidak’s *post-hoc* test | *p*=0.0083 | F _(1, 4)_ = 4087 |
|  | SMP_2h *vs.* Vehicle_2h | Repeated-measures two-way ANOVA with Sidak’s *post-hoc* test | *p*=0.0079 |  |
|  | SMP_3h *vs.* Vehicle_3h | Repeated-measures two-way ANOVA with Sidak’s *post-hoc* test | *p*=0.0003 |  |
|  | SMP_4h *vs.* Vehicle_4h | Repeated-measures two-way ANOVA with Sidak’s *post-hoc* test | *p*<0.0001 |  |
| 2E | SMP_4h *vs.* Vehicle_4h | Two-tailed unpaired *t* test with Welch's correction | *p*<0.0001 | t _(18.42)_ = 5.850 |
| 2P | SMP *vs.* Vehicle | Two-tailed unpaired *t* test with Welch's correction | *p*=0.0002 | t _(5)_ = 9.733 |
| 2Q | SMP *vs.* Vehicle | Two-tailed unpaired *t* test with Welch's correction | *p*=0.0005 | t _(5)_ =7.992 |
| 3C | SMP *vs.* Vehicle (Longest protrusion length) | Two-tailed unpaired *t* test with Welch's correction | *p*<0.0001 | t _(8.055)_ =7.405 |
|  | SMP *vs.* Vehicle (Shortest protrusion length) | Two-tailed unpaired *t* test with Welch's correction | *p*=0.0152 | t _(8.023)_ =3.075 |
|  | SMP *vs.* Vehicle (Average protrusion length) | Two-tailed unpaired *t* test with Welch's correction | *p*=0.0004 | t _(8.032)_ =5.748 |
|  | SMP *vs.* Vehicle (Total protrusion length) | Two-tailed unpaired *t* test with Welch's correction | *p*<0.0001 | t _(8.211)_ =16.42 |
| 3D | SMP *vs.* Vehicle (Protrusions number) | Two-tailed unpaired *t* test | *p*<0.0001 | t _(16)_ =6.405 |
|  | SMP *vs.* Vehicle (Nodes number) | Two-tailed unpaired *t* test with Welch's correction | *p*=0.0018 | t _(8)_ =4.6 |
|  | SMP *vs.* Vehicle (Segments number) | Two-tailed unpaired *t* test with Welch's correction | *p<*0.0001 | t _(8)_ =7.588 |
|  | SMP *vs.* Vehicle (Ends number) | Two-tailed unpaired *t* test with Welch's correction | *p*<0.0001 | t _(9.034)_ =6.988 |
| 3E | SMP *vs.* Vehicle (Soma long diameter) | Two-tailed unpaired *t* test | *p*=0.0048 | t _(16)_ =3.272 |
|  | SMP *vs.* Vehicle (Soma short diameter) | Two-tailed unpaired *t* test | *p*=0.1029 | t _(16)_ =1.730 |
|  | SMP *vs.* Vehicle (Soma mean diameter) | Two-tailed unpaired *t* test | *p*=0.0187 | t _(16)_ =2.617 |
|  | SMP *vs.* Vehicle (Soma perimeter) | Two-tailed unpaired *t* test | *p*=0.0292 | t _(16)_ =2.396 |
|  | SMP *vs.* Vehicle (Soma surface) | Two-tailed unpaired *t* test | *p*=0.0357 | t _(16)_ =2.293 |
| 3F | SMP *vs.* Vehicle | Repeated-measures two-way ANOVA with Sidak’s *post-hoc* test | *p*<0.0001 | F _(1,704)_ = 1046 |
| 3H | SMP *vs.* SMP-Y | One-way ANOVA with Dunnett’s *post-hoc* test | *p*<0.0001 | F _(10,22)_ = 46.78 |
|  | SMP *vs.* SMP-F | One-way ANOVA with Dunnett’s *post-hoc* test | *p*<0.0001 |  |
|  | SMP *vs.* SMP-I | One-way ANOVA with Dunnett’s *post-hoc* test | *p*<0.0001 |  |
|  | SMP *vs.* SMP-db | One-way ANOVA with Dunnett’s *post-hoc* test | *p*<0.0001 |  |
|  | SMP *vs.* SMP-G | One-way ANOVA with Dunnett’s *post-hoc* test | *p*=0.0005 |  |
|  | SMP *vs.* SMP-L | One-way ANOVA with Dunnett’s *post-hoc* test | *p*=0.1262 |  |
|  | SMP *vs.* SMP-K | One-way ANOVA with Dunnett’s *post-hoc* test | *p*=0.1450 |  |
|  | SMP *vs.* SMP-N | One-way ANOVA with Dunnett’s *post-hoc* test | *p*=0.5190 |  |
|  | SMP *vs.* SMP-P | One-way ANOVA with Dunnett’s *post-hoc* test | *p*=0.7512 |  |
|  | SMP *vs.* SMP-D | One-way ANOVA with Dunnett’s *post-hoc* test | *p*=0.9973 |  |
| 3J | SMP *vs.* SMP-F/db | One-way ANOVA with Dunnett’s *post-hoc* test | *p*<0.0001 | F _(3,8)_ = 497.0 |
|  | SMP *vs.* SMP-I/G | One-way ANOVA with Dunnett’s *post-hoc* test | *p*<0.0001 |  |
|  | SMP *vs.* SMP-F/db/I/G/Y | One-way ANOVA with Dunnett’s *post-hoc* test | *p*<0.0001 |  |
| 3M | SMP *vs.* SMP+AA (2.5 μM) | One-way ANOVA with Dunnett’s *post-hoc* test | *p*=0.0996 | F _(6,14)_ =173.7 |
|  | SMP *vs.* SMP+AA (5 μM) | One-way ANOVA with Dunnett’s *post-hoc* test | *p*=0.3343 |  |
|  | SMP *vs.* SMP+AA (10 μM) | One-way ANOVA with Dunnett’s *post-hoc* test | *p*=0.0004 |  |
|  | SMP *vs.* SMP+AA (20 μM) | One-way ANOVA with Dunnett’s *post-hoc* test | *p*<0.0001 |  |
|  | SMP *vs.* SMP+AA (40 μM) | One-way ANOVA with Dunnett’s *post-hoc* test | *p*<0.0001 |  |
|  | SMP *vs.* SMP+AA (80 μM) | One-way ANOVA with Dunnett’s *post-hoc* test | *p*<0.0001 |  |
| 3O | SMP *vs.* SMP+ML-7 (1.25 μM) | One-way ANOVA with Dunnett’s *post-hoc* test | *p*=0.0066 | F _(6,14)_ =808.6 |
|  | SMP *vs.* SMP+ML-7 (2.5 μM) | One-way ANOVA with Dunnett’s *post-hoc* test | *p*<0.0001 |  |
|  | SMP *vs.* SMP+ML-7 (5 μM) | One-way ANOVA with Dunnett’s *post-hoc* test | *p*<0.0001 |  |
|  | SMP *vs.* SMP+ML-7 (10 μM) | One-way ANOVA with Dunnett’s *post-hoc* test | *p*<0.0001 |  |
|  | SMP *vs.* SMP+ML-7 (20 μM) | One-way ANOVA with Dunnett’s *post-hoc* test | *p*<0.0001 |  |
|  | SMP *vs.* SMP+ML-7 (40 μM) | One-way ANOVA with Dunnett’s *post-hoc* test | *p*<0.0001 |  |
| 4B | Ionomycin *vs.* SMP | One-way ANOVA with Dunnett’s *post-hoc* test | *p*<0.0001 | F _(2,6)_ =21935 |
|  | Thapsigargin *vs.* SMP | One-way ANOVA with Dunnett’s *post-hoc* test | *p*<0.0001 |  |
| 4F | SMP *vs.* SMP+D-AP5 (12.5 μM) | One-way ANOVA with Dunnett’s *post-hoc* test | *p*=0.2776 | F _(4,10)_ =2.040 |
|  | SMP *vs.* SMP+D-AP5 (25 μM) | One-way ANOVA with Dunnett’s *post-hoc* test | *p*=0.0988 |  |
|  | SMP *vs.* SMP+D-AP5 (50 μM) | One-way ANOVA with Dunnett’s *post-hoc* test | *p*=0.1123 |  |
|  | SMP *vs.* SMP+D-AP5 (100 μM) | One-way ANOVA with Dunnett’s *post-hoc* test | *p*=0.1927 |  |
| 4H | SMP *vs.* SMP+Cd^2+^ (1 μM) | One-way ANOVA with Dunnett’s *post-hoc* test | *p*=0.0232 | F _(4,10)_ =606.4 |
|  | SMP *vs.* SMP+Cd^2+^ (10 μM) | One-way ANOVA with Dunnett’s *post-hoc* test | *p*<0.0001 |  |
|  | SMP *vs.* SMP+Cd^2+^ (50 μM) | One-way ANOVA with Dunnett’s *post-hoc* test | *p*<0.0001 |  |
|  | SMP *vs.* SMP+Cd^2+^ (100 μM) | One-way ANOVA with Dunnett’s *post-hoc* test | *p*<0.0001 |  |
| 4J | SMP *vs.* SMP+Nimodipine (10 μM) | One-way ANOVA with Dunnett’s *post-hoc* test | *p*=0.1413 | F _(4,10)_ =697.4 |
|  | SMP *vs.* SMP+Nimodipine (20 μM) | One-way ANOVA with Dunnett’s *post-hoc* test | *p*<0.0001 |  |
|  | SMP *vs.* SMP+Nimodipine (40 μM) | One-way ANOVA with Dunnett’s *post-hoc* test | *p*<0.0001 |  |
|  | SMP *vs.* SMP+Nimodipine (80 μM) | One-way ANOVA with Dunnett’s *post-hoc* test | *p*<0.0001 |  |
| 4L | SMP *vs.* SMP+Nisoldipine (5 μM) | One-way ANOVA with Dunnett’s *post-hoc* test | *p*=0.6528 | F _(4,10)_ =1717 |
|  | SMP *vs.* SMP+Nisoldipine (10 μM) | One-way ANOVA with Dunnett’s *post-hoc* test | *p*<0.0001 |  |
|  | SMP *vs.* SMP+Nisoldipine (20 μM) | One-way ANOVA with Dunnett’s *post-hoc* test | *p*<0.0001 |  |
|  | SMP *vs.* SMP+Nisoldipine (40 μM) | One-way ANOVA with Dunnett’s *post-hoc* test | *p*<0.0001 |  |
| 4P | Mock-KO *vs.* CACNA1C-KO | One-way ANOVA with Dunnett’s *post-hoc* test | *p*<0.0001 | F _(2,6)_ =1412 |
|  | Mock-KO *vs.* CACNA1D-KO | One-way ANOVA with Dunnett’s *post-hoc* test | *p*<0.0001 |  |
| 4Q | Mock-KO *vs.* CACNA1C-KO (Protrusions number) | One-way ANOVA with Dunnett’s *post-hoc* test | *p*=0.3124 | F _(2,24)_ =0.9418 |
|  | Mock-KO *vs.* CACNA1D-KO (Protrusions number) | One-way ANOVA with Dunnett’s *post-hoc* test | *p*=0.6012 |  |
|  | Mock-KO *vs.* CACNA1C-KO (Nodes number) | One-way ANOVA with Dunnett’s *post-hoc* test | *p*<0.0001 | F _(2,24)_ =17.14 |
|  | Mock-KO *vs.* CACNA1D-KO (Nodes number) | One-way ANOVA with Dunnett’s *post-hoc* test | *p*<0.0001 |  |
|  | Mock-KO *vs.* CACNA1C-KO (Ends number) | One-way ANOVA with Dunnett’s *post-hoc* test | *p*=0.0005 | F _(2,24)_ =11.13 |
|  | Mock-KO *vs.* CACNA1D-KO (Ends number) | One-way ANOVA with Dunnett’s *post-hoc* test | *p*=0.0014 |  |
| 4R | Mock-KO *vs.* CACNA1C-KO (Total protrusion length) | One-way ANOVA with Dunnett’s *post-hoc* test | *p*<0.0001 | F _(2,24)_ =31.30 |
|  | Mock-KO *vs.* CACNA1D-KO (Total protrusion length) | One-way ANOVA with Dunnett’s *post-hoc* test | *p*<0.0001 |  |
|  | Mock-KO *vs.* CACNA1C-KO (Average protrusion length) | One-way ANOVA with Dunnett’s *post-hoc* test | *p*<0.0001 | F _(2,24)_ =42.41 |
|  | Mock-KO *vs.* CACNA1D-KO (Average protrusion length) | One-way ANOVA with Dunnett’s *post-hoc* test | *p*<0.0001 |  |
|  | Mock-KO *vs.* CACNA1C-KO (Longest protrusion length) | One-way ANOVA with Dunnett’s *post-hoc* test | *p*<0.0001 | F _(2,24)_ =87.38 |
|  | Mock-KO *vs.* CACNA1D-KO  (Longest protrusion length) | One-way ANOVA with Dunnett’s *post-hoc* test | *p*<0.0001 |  |
|  | Mock-KO *vs.* CACNA1C-KO (Shortest protrusion length) | One-way ANOVA with Dunnett’s *post-hoc* test | *p*=0.0062 | F _(2,24)_ =7.369 |
|  | Mock-KO *vs.* CACNA1D-KO (Shortest protrusion length) | One-way ANOVA with Dunnett’s *post-hoc* test | *p*=0.0048 |  |
| 4S | Mock-KO *vs.* CACNA1C-KO | Repeated-measures two-way ANOVA with Sidak’s *post-hoc* test | *p*<0.0001 | F _(1, 16)_ = 28.50 |
|  | Mock-KO *vs.* CACNA1D-KO | Repeated-measures two-way ANOVA with Sidak’s *post-hoc* test | *p*<0.0001 | F _(1, 16)_ = 29.08 |
| 6I | SMP_d3 *vs.* SMP_d0 | One-way ANOVA with Dunnett’s *post-hoc* test | *p*=0.0016 | F _(2,6)_ =84.45 |
|  | SMP_d15 *vs.* SMP_d0 | One-way ANOVA with Dunnett’s *post-hoc* test | *p*<0.0001 |  |
| 6J | SMP_d3 *vs.* SMP_d0 | One-way ANOVA with Dunnett’s *post-hoc* test | *p*=0.0002 | F _(2,6)_ =1268 |
|  | SMP_d15 *vs.* SMP_d0 | One-way ANOVA with Dunnett’s *post-hoc* test | *p*<0.0001 |  |
| 6L | SMP_d3 *vs.* SMP_d0 | One-way ANOVA with Dunnett’s *post-hoc* test | *p*<0.0001 | F _(2,6)_ =195.4 |
|  | SMP_d15 *vs.* SMP_d0 | One-way ANOVA with Dunnett’s *post-hoc* test | *p*<0.0001 |  |
| 6N | LV-shECEL1-SMP-d3 *vs.* LV-mCherry-SMP-d3 | Two-tailed unpaired *t* test with Welch's correction | *p*=0.0014 | t _(2.011)_ =25.97 |
| 6O | LV-shECEL1-SMP-d3 *vs.* LV-mCherry-SMP-d3 | Two-tailed unpaired *t* test with Welch's correction | *p*=0.0046 | t _(2.001)_ =14.67 |
| 6P | LV-shECEL1-SMP-d3 *vs.* LV-mCherry-SMP-d3 | Two-tailed unpaired *t* test with Welch's correction | *p*=0.0035 | t _(2.078)_ =15.60 |
| 6R | LV-shECEL1-SMP-d3 *vs.* LV-mCherry-SMP-d3 | Two-tailed unpaired *t* test with Welch's correction | *p*<0.0001 | t _(19.67)_ =6.621 |
| 7B | LV-OE-ECEL1-vehicle-d3 *vs.* LV-gcGFP-vehicle-d3 | Two-tailed unpaired *t* test with Welch's correction | *p*=0.0012 | t _(3.097)_ =11.52 |
| 7C | LV-OE-ECEL1-vehicle-d3 *vs.* LV-gcGFP-vehicle-d3 | Two-tailed unpaired *t* test with Welch's correction | *p*=0.0070 | t _(3.279)_ =6.058 |
| 7D | LV-OE-ECEL1-vehicle-d3 *vs.* LV-gcGFP-vehicle-d3 | Two-tailed unpaired *t* test | *p*<0.0001 | t _(6)_ =15.83 |
| 7F | LV-OE-ECEL1-vehicle-d3 *vs.* LV-gcGFP-vehicle-d3 | Two-tailed unpaired *t* test with Welch's correction | *p*=0.0071 | t _(23.15)_ =2.951 |
| 7J | SMP_d3 *vs.* SMP_d0 | Two-tailed unpaired *t* test | *p*<0.0001 | t _(4)_ =29.32 |
| 7K | LV-shECEL1-SMP-d3 *vs.* LV-mCherry-SMP-d3 | Two-tailed unpaired *t* test | *p*<0.0001 | t _(4)_ =38.16 |
| 7L | LV-OE-ECEL1-vehicle-d3 *vs.* LV-gcGFP-vehicle-d3 | Two-tailed unpaired *t* test | *p*=0.0017 | t _(4)_ =7.537 |
| 7O | Scrambled siRNA *vs.* CALM3 siRNA-1# | One-way ANOVA with Dunnett’s *post-hoc* test | *p*<0.0001 | F _(3,8)_ =51.45 |
|  | Scrambled siRNA *vs.* CALM3 siRNA-2# | One-way ANOVA with Dunnett’s *post-hoc* test | *p*<0.0001 |  |
|  | Scrambled siRNA *vs.* CALM3 siRNA-3# | One-way ANOVA with Dunnett’s *post-hoc* test | *p*=0.0021 |  |
| 7P | CALM3 siRNA-SMP_d3 *vs.* Scrambled siRNA-SMP_d3 | Two-tailed unpaired *t* test | *p*=0.0023 | t _(4)_ =6.923 |
| 7Q | CALM3 siRNA-SMP_d3 *vs.* Scrambled siRNA-SMP_d3 | Two-tailed unpaired *t* test | *p*=0.0022 | t _(4)_ =6.999 |
| 7R | CALM3 siRNA-SMP_d3 *vs.* Scrambled siRNA-SMP_d3 | Two-tailed unpaired *t* test | *p*=0.0017 | t _(4)_ =7.437 |
| 7T | CALM3 siRNA-SMP_d3 *vs.* Scrambled siRNA-SMP_d3 | Two-tailed unpaired *t* test | *p*=0.0041 | t _(24)_ =3.170 |
| 8B | CALM3 siRNA-vehicle_d3 *vs.* Scrambled siRNA-vehicle_d3 | Two-tailed unpaired *t* test | *p*=0.0017 | t _(4)_ =7.528 |
| 8C | CALM3 siRNA-vehicle_d3 *vs.* Scrambled siRNA-vehicle_d3 | Two-tailed unpaired *t* test with Welch's correction | *p*=0.0308 | t _(2.068)_ =5.360 |
| 8D | CALM3 siRNA-vehicle_d3 *vs.* Scrambled siRNA-vehicle_d3 | Two-tailed unpaired *t* test | *p*=0.0002 | t _(4)_ =12.46 |
| 8F | CALM3 siRNA-vehicle_d3 *vs.* Scrambled siRNA-vehicle_d3 | Two-tailed unpaired *t* test with Welch's correction | *p*=0.0330 | t _(8.307)_ =2.554 |
| 8K | LV-shECEL1-OE-CALM3-SMP_d3 *vs.*  LV-shECEL1-gcGFP-SMP_d3 | Two-tailed unpaired *t* test | *p*<0.0001 | t _(4)_ =100.3 |
| 8L | LV-shECEL1-OE-CALM3-SMP_d3 *vs.*  LV-shECEL1-gcGFP-SMP_d3 | Two-tailed unpaired *t* test with Welch's correction | *p*=0.0055 | t _(2.058)_ =12.73 |
| 8M | LV-shECEL1-OE-CALM3-SMP_d3 *vs.*  LV-shECEL1-gcGFP-SMP_d3 | Two-tailed unpaired *t* test with Welch's correction | *p*=0.0125 | t _(2.042)_ =8.587 |
| 8N | LV-shECEL1-OE-CALM3-SMP_d3 *vs.*  LV-shECEL1-gcGFP-SMP_d3 | Two-tailed unpaired *t* test with Welch's correction | *p*=0.0017 | t _(2.008)_ =24.28 |
| 8P | LV-shECEL1-OE-CALM3-SMP_d3 *vs.*  LV-shECEL1-gcGFP-SMP_d3 | Two-tailed unpaired *t* test with Welch's correction | *p*=0.0037 | t _(13.99)_ =3.481 |
| S4B | SMP_4h *vs.* Vehicle_4h | Two-tailed unpaired *t* test with Welch's correction | *p*=0.0104 | t _(4.295)_ =4.348 |
| S4E | SMP_d3 *vs.* SMP_d0 | Two-tailed unpaired *t* test with Welch's correction | *p*=0.0069 | t _(2.028)_ =11.64 |
| S4F | SMP_d3 *vs.* SMP_d0 | Two-tailed unpaired *t* test | *p*=0.0007 | t _(4)_ =9.606 |
| S4L | SMP_d30 *vs.* Vehicle_d30 | Two-tailed unpaired *t* test | *p*=0.0018 | t _(8)_ =4.597 |
| S4M | SMP_d30 *vs.* Vehicle_d30 | Two-tailed unpaired *t* test | *p*=0.0005 | t _(8)_ =5.685 |
| S4P | SMP_d60 *vs.* Vehicle_d60 | Two-tailed unpaired *t* test with Welch's correction | *p*=0.0017 | t _(5)_ =6.099 |
| S4T | SMP_d60 *vs.* Vehicle_d60 (Broadband) | Repeated-measures two-way ANOVA with Sidak’s *post-hoc* test | *p*<0.0001 | F _(1, 10)_ = 4929 |
|  | SMP_d60 *vs.* Vehicle_d60 (Theta) | Repeated-measures two-way ANOVA with Sidak’s *post-hoc* test | *p*<0.0001 |  |
|  | SMP_d60 *vs.* Vehicle_d60 (Alpha) | Repeated-measures two-way ANOVA with Sidak’s *post-hoc* test | *p*<0.0001 |  |
|  | SMP_d60 *vs.* Vehicle_d60 (Beta) | Repeated-measures two-way ANOVA with Sidak’s *post-hoc* test | *p*<0.0001 |  |
|  | SMP_d60 *vs.* Vehicle_d60 (Gamma) | Repeated-measures two-way ANOVA with Sidak’s *post-hoc* test | *p*<0.0001 |  |
| S5A | SMP *vs.* SMP+Cd^2+^ (1 μM) | One-way ANOVA with Dunnett’s *post-hoc* test | *p*=0.9991 | F _(4, 10)_ =0.3390 |
|  | SMP *vs.* SMP+Cd^2+^ (10 μM) | One-way ANOVA with Dunnett’s *post-hoc* test | *p*=0.8168 |  |
|  | SMP *vs.* SMP+Cd^2+^ (50 μM) | One-way ANOVA with Dunnett’s *post-hoc* test | *p*=0.9737 |  |
|  | SMP *vs.* SMP+Cd^2+^ (100 μM) | One-way ANOVA with Dunnett’s *post-hoc* test | *p*=0.9979 |  |
| S5B | SMP *vs.* SMP+Cd^2+^ (1 μM) | One-way ANOVA with Dunnett’s *post-hoc* test | *p*=0.9947 | F _(4, 10)_ =0.7417 |
|  | SMP *vs.* SMP+Cd^2+^ (10 μM) | One-way ANOVA with Dunnett’s *post-hoc* test | *p*=0.9765 |  |
|  | SMP *vs.* SMP+Cd^2+^ (50 μM) | One-way ANOVA with Dunnett’s *post-hoc* test | *p*=0.4456 |  |
|  | SMP *vs.* SMP+Cd^2+^ (100 μM) | One-way ANOVA with Dunnett’s *post-hoc* test | *p*=0.62 |  |
| S5C | SMP *vs.* SMP+Cd^2+^ (1 μM) | One-way ANOVA with Dunnett’s *post-hoc* test | *p*=0.9825 | F _(4, 10)_ =0.5913 |
|  | SMP *vs.* SMP+Cd^2+^ (10 μM) | One-way ANOVA with Dunnett’s *post-hoc* test | *p*=0.9999 |  |
|  | SMP *vs.* SMP+Cd^2+^ (50 μM) | One-way ANOVA with Dunnett’s *post-hoc* test | *p*=0.5942 |  |
|  | SMP *vs.* SMP+Cd^2+^ (100 μM) | One-way ANOVA with Dunnett’s *post-hoc* test | *p*=0.8397 |  |
| S5E | SMP *vs.* SMP+Cd^2+^ (1 μM) | One-way ANOVA with Dunnett’s *post-hoc* test | *p*=0.8843 | F _(4, 10)_ =0.7780 |
|  | SMP *vs.* SMP+Cd^2+^ (10 μM) | One-way ANOVA with Dunnett’s *post-hoc* test | *p*=0.8082 |  |
|  | SMP *vs.* SMP+Cd^2+^ (50 μM) | One-way ANOVA with Dunnett’s *post-hoc* test | *p*=0.9973 |  |
|  | SMP *vs.* SMP+Cd^2+^ (100 μM) | One-way ANOVA with Dunnett’s *post-hoc* test | *p*=0.8832 |  |
| S5F | SMP *vs.* SMP+Nimodipine (10 μM) | One-way ANOVA with Dunnett’s *post-hoc* test | *p*=0.7507 | F _(4, 10)_ =0.2634 |
|  | SMP *vs.* SMP+Nimodipine (20 μM) | One-way ANOVA with Dunnett’s *post-hoc* test | *p*=0.9768 |  |
|  | SMP *vs.* SMP+Nimodipine (40 μM) | One-way ANOVA with Dunnett’s *post-hoc* test | *p*=0.8506 |  |
|  | SMP *vs.* SMP+Nimodipine (80 μM) | One-way ANOVA with Dunnett’s *post-hoc* test | *p*=0.9567 |  |
| S5G | SMP *vs.* SMP+Nimodipine (10 μM) | One-way ANOVA with Dunnett’s *post-hoc* test | *p*=0.9961 | F _(4, 10)_ =0.7801 |
|  | SMP *vs.* SMP+Nimodipine (20 μM) | One-way ANOVA with Dunnett’s *post-hoc* test | *p*=0.51 |  |
|  | SMP *vs.* SMP+Nimodipine (40 μM) | One-way ANOVA with Dunnett’s *post-hoc* test | *p*=0.9823 |  |
|  | SMP *vs.* SMP+Nimodipine (80 μM) | One-way ANOVA with Dunnett’s *post-hoc* test | *p*=0.51 |  |
| S5H | SMP *vs.* SMP+Nimodipine (10 μM) | One-way ANOVA with Dunnett’s *post-hoc* test | *p*=0.924 | F _(4, 10)_ =0.4608 |
|  | SMP *vs.* SMP+Nimodipine (20 μM) | One-way ANOVA with Dunnett’s *post-hoc* test | *p*=0.5705 |  |
|  | SMP *vs.* SMP+Nimodipine (40 μM) | One-way ANOVA with Dunnett’s *post-hoc* test | *p*=0.8915 |  |
|  | SMP *vs.* SMP+Nimodipine (80 μM) | One-way ANOVA with Dunnett’s *post-hoc* test | *p*=0.6684 |  |
| S5J | SMP *vs.* SMP+Nimodipine (10 μM) | One-way ANOVA with Dunnett’s *post-hoc* test | *p*>0.9999 | F _(4, 10)_ =0.8123 |
|  | SMP *vs.* SMP+Nimodipine (20 μM) | One-way ANOVA with Dunnett’s *post-hoc* test | *p*=0.9879 |  |
|  | SMP *vs.* SMP+Nimodipine (40 μM) | One-way ANOVA with Dunnett’s *post-hoc* test | *p*=0.6936 |  |
|  | SMP *vs.* SMP+Nimodipine (80 μM) | One-way ANOVA with Dunnett’s *post-hoc* test | *p*=0.7395 |  |
| S5K | SMP *vs.* SMP+Nisoldipine (5 μM) | One-way ANOVA with Dunnett’s *post-hoc* test | *p*=0.6422 | F _(4, 10)_ =1.092 |
|  | SMP *vs.* SMP+Nisoldipine (10 μM) | One-way ANOVA with Dunnett’s *post-hoc* test | *p*=0.8156 |  |
|  | SMP *vs.* SMP+Nisoldipine (20 μM) | One-way ANOVA with Dunnett’s *post-hoc* test | *p*=0.999 |  |
|  | SMP *vs.* SMP+Nisoldipine (40 μM) | One-way ANOVA with Dunnett’s *post-hoc* test | *p*=0.9696 |  |
| S5L | SMP *vs.* SMP+Nisoldipine (5 μM) | One-way ANOVA with Dunnett’s *post-hoc* test | *p*=0.7905 | F _(4, 10)_ =0.3280 |
|  | SMP *vs.* SMP+Nisoldipine (10 μM) | One-way ANOVA with Dunnett’s *post-hoc* test | *p*>0.9999 |  |
|  | SMP *vs.* SMP+Nisoldipine (20 μM) | One-way ANOVA with Dunnett’s *post-hoc* test | *p*=0.9183 |  |
|  | SMP *vs.* SMP+Nisoldipine (40 μM) | One-way ANOVA with Dunnett’s *post-hoc* test | *p*=0.9998 |  |
| S5M | SMP *vs.* SMP+Nisoldipine (5 μM) | One-way ANOVA with Dunnett’s *post-hoc* test | *p*=0.9999 | F _(4, 10)_ =0.9765 |
|  | SMP *vs.* SMP+Nisoldipine (10 μM) | One-way ANOVA with Dunnett’s *post-hoc* test | *p*=0.5986 |  |
|  | SMP *vs.* SMP+Nisoldipine (20 μM) | One-way ANOVA with Dunnett’s *post-hoc* test | *p*=0.9828 |  |
|  | SMP *vs.* SMP+Nisoldipine (40 μM) | One-way ANOVA with Dunnett’s *post-hoc* test | *p*=0.3715 |  |
| S5N | SMP *vs.* SMP+Nisoldipine (5 μM) | One-way ANOVA with Dunnett’s *post-hoc* test | *p*=0.9908 | F _(4, 10)_ =0.2418 |
|  | SMP *vs.* SMP+Nisoldipine (10 μM) | One-way ANOVA with Dunnett’s *post-hoc* test | *p*=0.8554 |  |
|  | SMP *vs.* SMP+Nisoldipine (20 μM) | One-way ANOVA with Dunnett’s *post-hoc* test | *p*=0.8686 |  |
|  | SMP *vs.* SMP+Nisoldipine (40 μM) | One-way ANOVA with Dunnett’s *post-hoc* test | *p*=0.8477 |  |
| S5Q | SMP *vs.* SMP+ω-Conotoxin MVIIC (1 μM) | One-way ANOVA with Dunnett’s *post-hoc* test | *p*=0.2492 | F _(3,8)_ =1.509 |
|  | SMP *vs.* SMP+ω-Conotoxin MVIIC (2 μM) | One-way ANOVA with Dunnett’s *post-hoc* test | *p*=0.9736 |  |
|  | SMP *vs.* SMP+ω-Conotoxin MVIIC (4 μM) | One-way ANOVA with Dunnett’s *post-hoc* test | *p*=0.3628 |  |
| S5S | SMP *vs.* SMP+SNX 482 (0.5 μM) | One-way ANOVA with Dunnett’s *post-hoc* test | *p*=0.9730 | F _(3,8)_ =0.7853 |
|  | SMP *vs.* SMP+SNX 482 (1 μM) | One-way ANOVA with Dunnett’s *post-hoc* test | *p*=0.5464 |  |
|  | SMP *vs.* SMP+SNX 482 (2 μM) | One-way ANOVA with Dunnett’s *post-hoc* test | *p*=0.9822 |  |
| S5U | SMP *vs.* SMP+Ethosuximide (20 μM) | One-way ANOVA with Dunnett’s *post-hoc* test | *p*=0.2554 | F _(3,8)_ =1.041 |
|  | SMP *vs.* SMP+Ethosuximide (40 μM) | One-way ANOVA with Dunnett’s *post-hoc* test | *p*=0.7774 |  |
|  | SMP *vs.* SMP+Ethosuximide (80 μM) | One-way ANOVA with Dunnett’s *post-hoc* test | *p*=0.7727 |  |
| S6F | LV-shCACNA1C *vs.* LV-mCherry | Two-tailed unpaired *t* test | *p*=0.8523 | t _(4)_ =0.1985 |
| S6G | LV-shCACNA1D *vs.* LV-gcGFP | Two-tailed unpaired *t* test | *p*=0.9304 | t _(4)_ =0.09294 |
| S6I | LV-shCACNA1C *vs.* LV-mCherry | Two-tailed unpaired *t* test | *p*=0.8015 | t _(4)_ =0.2687 |
| S6K | LV-shCACNA1D *vs.* LV-gcGFP | Two-tailed unpaired *t* test | *p*=0.1056 | t _(4)_ =2.083 |
| S8E | LV-mCherry *vs.* LV-shECEL1-1# | One-way ANOVA with Dunnett’s *post-hoc* test | *p*=0.0085 | F _(3,8)_ =9.492 |
|  | LV-mCherry *vs.* LV-shECEL1-2# | One-way ANOVA with Dunnett’s *post-hoc* test | *p*=0.0156 |  |
|  | LV-mCherry *vs.* LV-shECEL1-3# | One-way ANOVA with Dunnett’s *post-hoc* test | *p*=0.9804 |  |
| S8G | LV-mCherry *vs.* LV-shECEL1-1# | Two-tailed unpaired *t* test | *p*=0.0018 | t _(4)_ =7.356 |
| S8J | LV-OE-ECEL1 *vs.* LV-gcGFP | Two-tailed unpaired *t* test with Welch's correction | *p*=0.0407 | t _(2.013)_ =4.772 |
| S8L | LV-OE-ECEL1 *vs.* LV-gcGFP | Two-tailed unpaired *t* test | *p*<0.0001 | t _(4)_ =21.14 |
| S8O | Vehicle *vs.* db-cAMP | One-way ANOVA with Dunnett’s *post-hoc* test | *p*<0.0001 | F _(10,22)_ =34.87 |
|  | Vehicle *vs.* Forskolin | One-way ANOVA with Dunnett’s *post-hoc* test | *p*=0.0016 |  |
|  | Vehicle *vs.* ISX9 | One-way ANOVA with Dunnett’s *post-hoc* test | *p*=0.1695 |  |
|  | Vehicle *vs.* Y27632 | One-way ANOVA with Dunnett’s *post-hoc* test | *p*=0.7207 |  |
|  | Vehicle *vs.* DAPT | One-way ANOVA with Dunnett’s *post-hoc* test | *p*=0.9635 |  |
|  | Vehicle *vs.* P7C3-A20 | One-way ANOVA with Dunnett’s *post-hoc* test | *p*=0.9994 |  |
|  | Vehicle *vs.* Nicotinamide | One-way ANOVA with Dunnett’s *post-hoc* test | *p*=0.9994 |  |
|  | Vehicle *vs.* L-ascorbic acid | One-way ANOVA with Dunnett’s *post-hoc* test | *p*=0.9996 |  |
|  | Vehicle *vs.* GABA | One-way ANOVA with Dunnett’s *post-hoc* test | *p*>0.9999 |  |
|  | Vehicle *vs.* Kenpaullone | One-way ANOVA with Dunnett’s *post-hoc* test | *p*>0.9999 |  |
| S8R | Scrambled siRNA *vs.* MEIS3 siRNA-1# | One-way ANOVA with Dunnett’s *post-hoc* test | *p*<0.0001 | F _(3,8)_ =3241 |
|  | Scrambled siRNA *vs.* MEIS3 siRNA-2# | One-way ANOVA with Dunnett’s *post-hoc* test | *p*<0.0001 |  |
|  | Scrambled siRNA *vs.* MEIS3 siRNA-3# | One-way ANOVA with Dunnett’s *post-hoc* test | *p*<0.0001 |  |
| S8S | Scrambled siRNA *vs.* TNNT1 siRNA-1# | One-way ANOVA with Dunnett’s *post-hoc* test | *p*<0.0001 | F _(3,8)_ =128.4 |
|  | Scrambled siRNA *vs.* TNNT1 siRNA-2# | One-way ANOVA with Dunnett’s *post-hoc* test | *p*<0.0001 |  |
|  | Scrambled siRNA *vs.* TNNT1 siRNA-3# | One-way ANOVA with Dunnett’s *post-hoc* test | *p*<0.0001 |  |
| S8U | Scrambled siRNA *vs.* MEIS3 siRNA | One-way ANOVA with Dunnett’s *post-hoc* test | *p*=0.9486 | F _(2, 36)_ = 0.03662 |
|  | Scrambled siRNA *vs.* TNNT1 siRNA | One-way ANOVA with Dunnett’s *post-hoc* test | *p*=0.9813 |  |

**Video Legends**

**Video S1-1.** **Dynamical monitor of changes in** **cellular morphology in the early stage (0-4 h) of vehicle-induced hiPSCs differentiation. Related to Figure 2.**

The representative videos showing the dynamical changes of cellular morphology during 4-hour culture with vehicle-induced hiPSCs differentiation. Video images were collected every 3 minutes for 4 hours (5% CO₂, 37 °C). This video demonstrates that the majority of vehicle-treated hiPSCs showed no process-bearing morphological changes within 4 hours. Scale bar=50 μm; time stamp shows hours: minutes: seconds. For more details, see Figure 2.

**Video S1-2. Dynamical monitor of changes in cellular morphology in the early stage (0-4 h) of SMP-induced hiPSCs differentiation. Related to Figure 2.**

The representative videos showing the dynamical changes of cellular morphology during 4-hour culture with SMP-induced hiPSCs differentiation. Video images were collected every 3 minutes for 4 hours (5% CO₂, 37 °C). The white arrow in video indicates a typical process-bearing cell. This video shows that the proportion of differentiated process-bearing of total hiPSCs in SMP treatment increased steadily within the 4 hours. Scale bar=50 μm; time stamp shows hours: minutes: seconds. For more details, see Figure 2.

**Video S2-1. Dynamical calcium fluorescence imaging of vehicle-induced, hiPSCs-differentiated cells (at 30 DIV) after 30 minutes treatment with Fluo-4 AM fluorescence probes. Related to Figure S4.**

The representative videos showing the dynamical calcium fluorescence imaging of vehicle-induced, hiPSCs-differentiated cells (at 30 DIV) after 30 minutes treatment with Fluo-4 AM fluorescence probes. The Fluo-4 fluorescence imaging was captured at 0.4 s intervals for 3 minutes, showing spontaneous calcium transients in hiPSCs-differentiated cells. Scale bar=20 μm; time stamp shows hours: minutes: seconds. For more details, see Figure S4.

**Video S2-2. Dynamical calcium fluorescence imaging of SMP-induced, hiPSCs-differentiated cells (at 30 DIV) after 30 minutes treatment with Fluo-4 AM fluorescence probes. Related to Figure S4.**

The representative videos showing the dynamical calcium fluorescence imaging of SMP-induced, hiPSCs-differentiated cells (at 30 DIV) after 30 minutes treatment with Fluo-4 AM fluorescence probes. The Fluo-4 fluorescence imaging was captured at 0.4 s intervals for 3 minutes, showing spontaneous calcium transients and synchronous activity across developing neural networks. Scale bar=20 μm; time stamp shows hours: minutes: seconds. For more details, see Figure S4.
